# Evolution of a Strategy for the Unified Synthesis of Enteropeptin Sactipeptides

**DOI:** 10.1021/acs.joc.5c03063

**Published:** 2026-02-23

**Authors:** Shuvendu Saha, Yiwei Zhang, Yesen Cheng, Chi P. Ting

**Affiliations:** Department of Chemistry, 8244 Brandeis University, 415 South Street, Waltham, Massachusetts 02453, United States

## Abstract

Sactipeptides are a class of natural product peptides with remarkable antibiotic properties that are defined by the presence of thioaminoketals in their structure. Recently, we reported the first total synthesis of a sactipeptide in our synthesis of enteropeptin A. The key to our synthesis involved the use of a dithiophosphoric acid catalyzed Markovnikov hydrothiolation of dehydroamino acids. With this reaction, thioaminoketals found in sactipeptides can be prepared directly from a dehydroamino acid and a cysteine residue. This article summarizes our initial approach toward enteropeptin synthesis and the evolution of our strategy that ultimately enabled the synthesis of these peptide natural products. Our first strategy involved late-stage Markovnikov hydrothiolation of an 8-mer peptide containing a dehydroamino acid and a cysteine residue that was unsuccessful. The second strategy involved an annulation reaction between a methyl ester of a dehydroamino acid and a cysteine with an unprotected amine that forged the central thiomorpholine ring albeit in low yield. The third strategy involved a divergent synthesis of the enteropeptins by early stage formation of the thiomorpholine ring by Markovnikov hydrothiolation followed by amidative coupling of the N- and C-terminal peptide fragments. This modular strategy enabled the unified synthesis of the enteropeptin sactipeptides.

## Graphical abstract

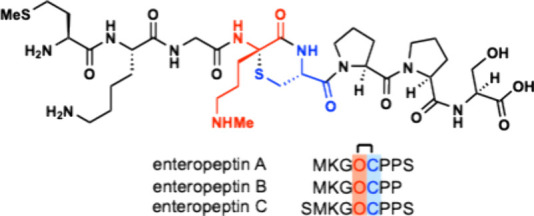

## Introduction

Sactipeptides are a rapidly growing subclass of ribosomally synthesized and post-translationally modified peptides (RiPPs).^1,2^ Sactipeptides are defined by the presence of α-thioethers in peptides which are formed from cysteine cyclization onto the α-carbon of an acceptor amino acid.^3–5^ This process assembles thioaminoketal rings, also known as sactionine linkages, within the peptide backbone.^6,7^ Four classes of sactipeptides have been isolated with each class corresponding to a different ring system found in natural sactipeptides. Type 1 sactipeptides were the first identified sactipeptides with subtilosin A (**1**) being the class-defining member.^8^ Vederas and co-workers characterized the structure of **1** and determined that it was a head-to-tail cyclization peptide with three sactionine linkages.^9^ Nuclear magnetic resonance (NMR) spectral analysis resulted in the stereochemical assignment of the sactionine linkages to be l-Phe22, d-Thr28, and d-Phe31.^8,9^ Since then, new Type 1 sactipeptides have been characterized containing a hairpin structure with highly nested topology containing three or four overlapping sactionine rings.^10^ They can also possess head-to-tail cyclization as is the case with subtilosin A. Streptosactin (**2**) was isolated in 2022 and is a Type 2 sactipeptide containing two nonoverlapping rings.^11^ In 2019, Duarte and co-workers isolated ruminococcin C1 (**3**) which is a Type 3 sactipeptide containing two pairs of overlapping sactionine rings.^12,13^ Finally, Seyedsayamdost and co-workers identified enteropeptins A-C as the first Type 4 sactipeptides containing a single sactionine ring. Enteropeptin A-C (**4a**-**c**) contain an unusual six-membered thiomorpholine ring with a thioaminoketal formed between neighboring cysteine and *N*-methylornithine residues (Figure 1).^14^

**Figure 1:**
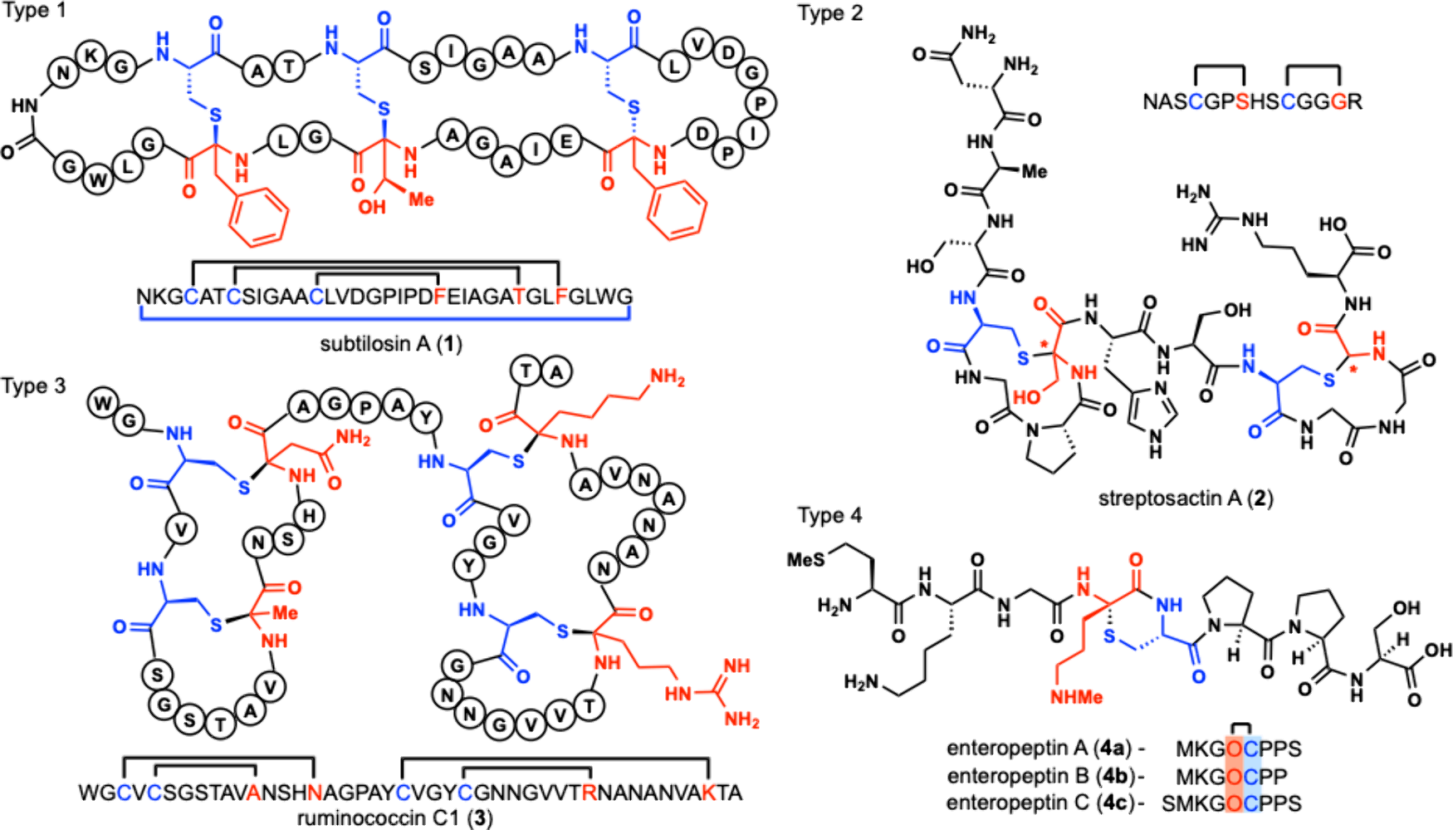
Representative members of the four types of sactipeptides. Single-letter amino acid abbreviations are used to depict the peptide sequence with black brackets to denote the sactionine cross-link and a blue bracket to denote head-to-tail cyclization. Cysteine residues are colored blue with acceptor amino acids colored red.

Sactipeptides are biosynthesized as ribosomal peptides that undergo carbon–sulfur bond formation between cysteine (**5**, C) and the acceptor amino acid (**6**, X) to form the sactionine linkage (**7**, Figure 2). The biosynthesis of sactipeptides involves radical S-adenosyl methionine (SAM) enzymes which forges the carbon–sulfur bond. The reaction is initiated by single electron reduction of the radical SAM enzyme which in turn reduces SAM to produce the deoxyadenosyl radical (dA•). The deoxyadenosyl radical then abstracts the α-proton of the acceptor amino acid resulting in a radical intermediate (**8**). Mechanistic studies by Bandarian group supports the formation of the captodative radical through a radical-clock experiment using a cyclopropylglycine residue.^4^ After radical formation, single electron transfer followed by concerted carbon–sulfur bond formation can occur to form the sactionine linkage (**7**). Alternatively, a stepwise mechanism has also been proposed involving oxidation of the radical to form iminium ion **9** which can undergo thiol addition via a two-electron mechanism (Figure 2).

**Figure 2:**
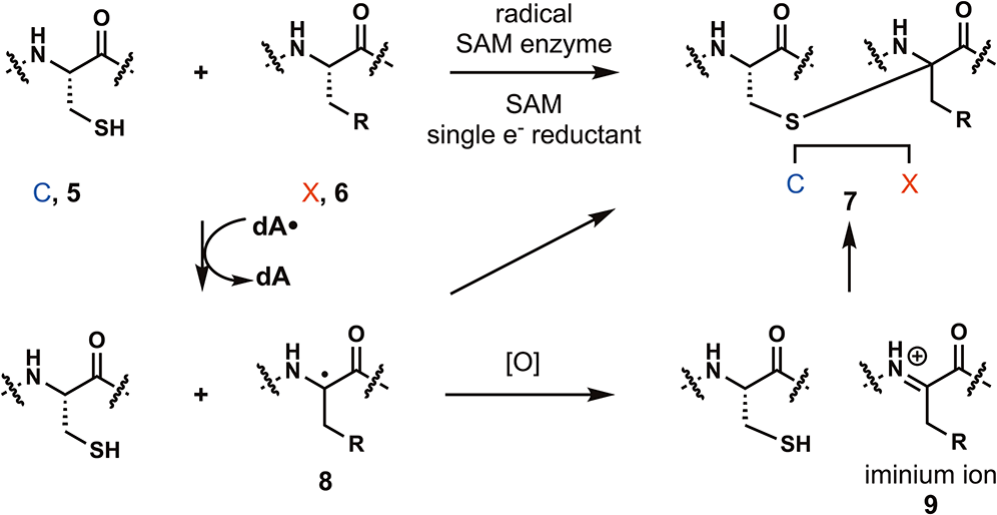
Biosynthesis of sactipeptides.

Inspired by Nature’s biosynthesis, many strategies for sactionine synthesis, including our own, utilized imine formation followed by thiol addition. Pioneering work by Vederas and co-workers showed that aminoketals can be converted to sulfur-containing thioaminoketals using tin­(IV) tetrachloride and benzyl thiol (Figure 3).^9^ The Lewis acid is expected to promote ionization of the alkoxide leaving group to generate the iminium ion which is intercepted by the thiol nucleophile. Antilla and co-workers reported the chiral phosphoric acid (CPA)-catalyzed addition of thiols to imines to make enantioenriched thioaminals.^15^ Recent advances in sactipeptide synthesis have been reported highlighting the interest in the synthesis of these antimicrobial peptides. In 2022, Malins reported the use of electrophilic glycines and their reaction with cysteine (Cys)-containing peptides.^16^ With base labile protecting groups such as fluorenylmethyloxycarbonyl (Fmoc), α-acetoxyglycines can be activated by boron trifluoride diethyl etherate for Cys addition. Alternatively with acid labile functionality such as *tert*-butoxycarbonyl (Boc), triethylamine can be used to generate imines from α-bromoglycine which can react with Cys-containing peptides. In 2024, Otaka and co-worker reported the synthesis of cyclic sactionines from a peptide containing a hydroxyamide and an acetamidomethyl (Acm)-protected Cys residue.^17^ After activation of the hydroxyamide with glycine thioester (H-Gly-SPh) in sodium phosphate buffer (pH 8.0), a Lossen rearrangement occurs to form an isocyanate. Water addition followed by decarboxylation results in an α-amino glycine. The resulting amine was treated with sodium nitrite under acidic conditions in water and was converted to the hemiaminal. Finally, thiol deprotection and imine formation occurs with guanidine hydrochloride and trifluoroacetic acid, and thiol addition to the imine forms the cyclic sactionine as a 1:1 mixture of diastereomers.

**Figure 3:**
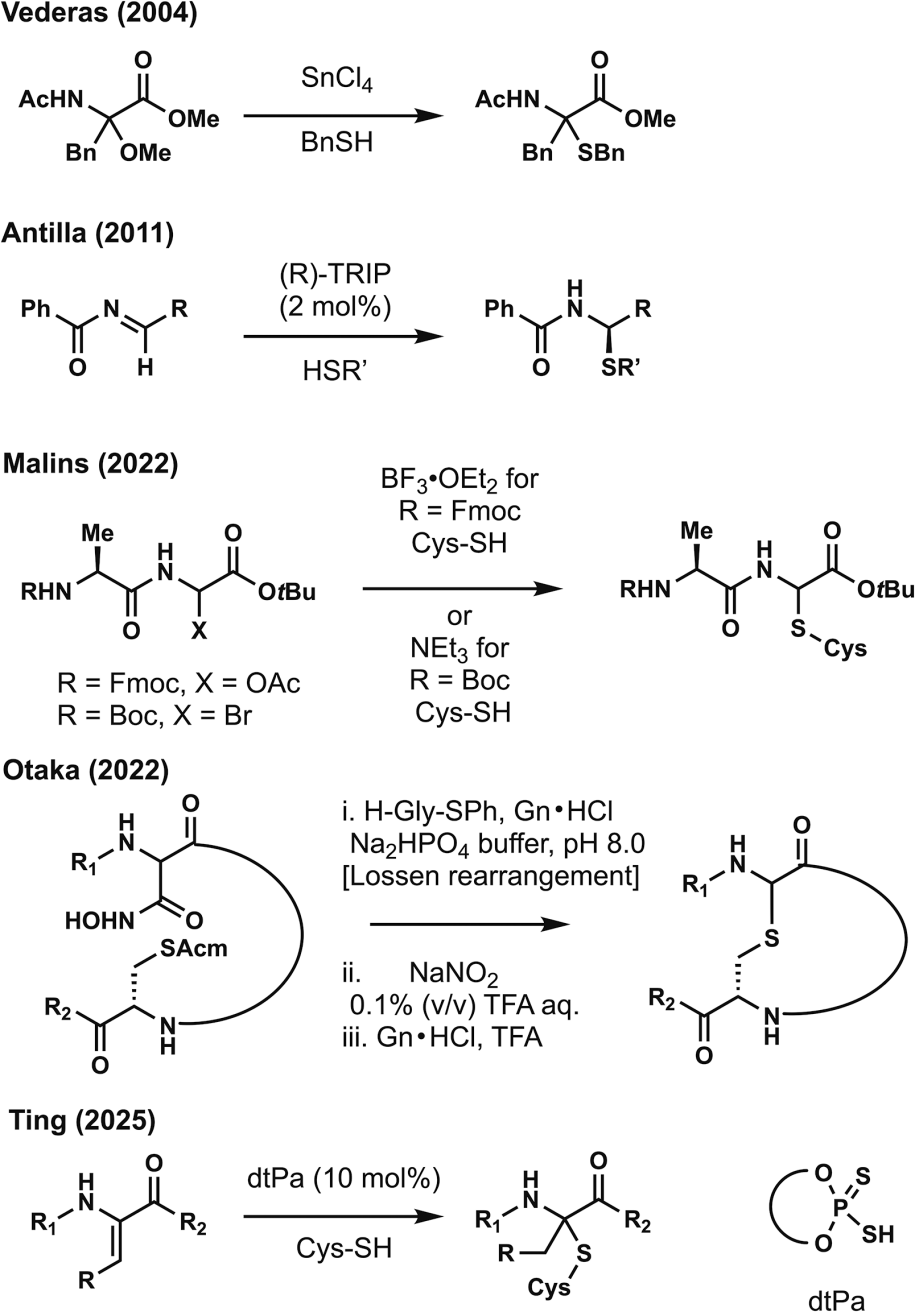
Prior strategies for the synthesis of sactionine cross-links.

These recent advances have enabled the synthesis of sactionine containing a thioaminal and can be potentially applied in the synthesis of the streptosactin (**2**) B ring. However, these strategies do not allow for the synthesis of the more substituted thioaminoketals that are found in all sactipeptides. In 2025, we reported the general Markovnikov hydrothiolation of dehydroamino acids (Dhaa) to access thioaminoketals found in sactipeptides (Figure 3).^18^ Central to our strategy was the use of a dithiophosphoric acid (dtPa) catalyst which selectively protonates the alkene of the dehydroamino acid and forms the iminium ion for thiol addition. This reaction was inspired by pioneering work in the area of chiral Bro̷nsted acid catalysis.^19–23^ Hydrofunctionalization of alkenes using chiral Bro̷nsted acid catalysts was reported by Yamamoto, List, and Toste.^24–30^ In 2011, Toste and co-worker reported the enantioselective, intramolecular hydroamination of dienes using dtPa catalysis.^30^ Inspired by this work, we examined different Bro̷nsted acid catalysts and found that the dithiophosphoric acid was essential for α-selective thiolation of dehydroamino acids. The method was applied in our total synthesis of enteropeptin A which utilized an intramolecular variant of this methodology.^31^ The reaction was also used in the total synthesis of enteropeptin B and C in 2025 which accomplished a unified synthesis of these sactipeptide natural products.^18^

## Results and Discussion

### First-Generation Approach toward Enteropeptin A by a Late-Stage Cyclization Strategy

In this article, we summarize the evolution of our strategy for the synthesis of the enteropeptins and describe several iterations of our synthetic approach toward enteropeptin A which culminated in the first total synthesis of a sactipeptide. Our first-generation retrosynthetic approach toward enteropeptin A (**4a**) involved late-stage formation of the thiomorpholine ring where cyclization of the 8-mer peptide (**10**) containing a dehydroornithine and a cysteine residue would forge the central thiomorpholine ring (Scheme 1). Global deprotection of acid-labile protecting groups would furnish the natural product. Peptide **10** could be obtained through convergent synthesis by amidative coupling of two tetrapeptides, carboxylic acid **11** and amine **12**. Acid **11** can be obtained from its corresponding *tert*-butyl ester and the dehydroamino acid can be formed from a Horner-Wadsworth-Emmons (HWE) reaction between phosphonate **13** and aldehyde **14** (Scheme 1). Phosphonate **13** can be obtained by solution phase peptide synthesis starting from (±)-Cbz-α-phosphonoglycine trimethyl ester and other commercially available amino acids.

**Scheme 1:**
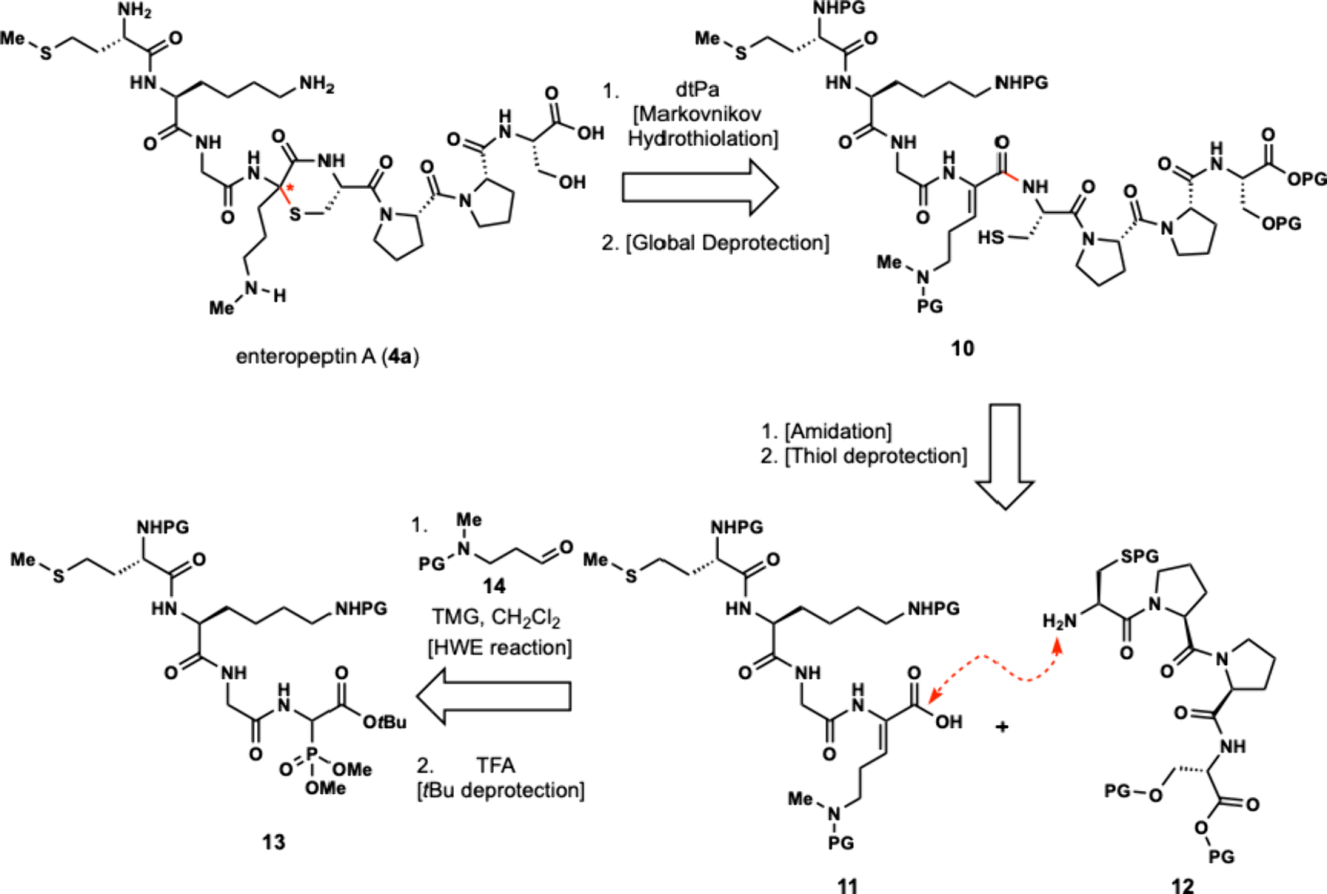
First-Generation Retrosynthesis of Enteropeptin A (**4a**)

In the forward direction, Cbz-Met-OH (**15**) was converted to its *N*-hydroxysuccinimide (NHS) ester with dicyclohexylcarbodiimide (DCC) and NHS (Scheme 2). Cbz-Met-NHS ester was then subjected to amidation with dipeptide **16** to generate the N-terminal tripeptide fragment (**17**) of enteropeptin A which was obtained in 73% yield over two steps. Dipeptide **16** was obtained in two steps by coupling of Boc-Lys­(Cbz)–OH (**18**) and glycine methyl ester hydrochloride salt (**19**) using EDC, HOBt, DIPEA to form dipeptide **20** in 56% yield. The N-terminal Boc group of **20** was removed using trifluoroacetic acid to produce dipeptide **16** in 83% yield as a trifluoroacetic acid salt (Scheme 3). The methyl ester of **17** was subjected to basic saponification to afford carboxylic acid **21** in 94% yield (Scheme 2). Acid **21** was then coupled to (±)-aminophosphonate **22** (prepared in three steps)^32^ using EDC, HOBt, and Hünig’s base to afford the tetrapeptide **23** as a 1:1 mixture of inconsequential diastereomers. Phosphonate **23** was then subjected to HWE reaction with aldehyde **24**. Using tetramethylguanidine as base and conditions developed by Boto and co-workers, the HWE reaction occurs with exclusive formation of the Z-dehydroamino acid (**25**).^33^ The *tert*-butyl ester of **25** was removed with ZnBr_2_ to furnish carboxylic acid **26** in 70% yield.^34^

**Scheme 2:**
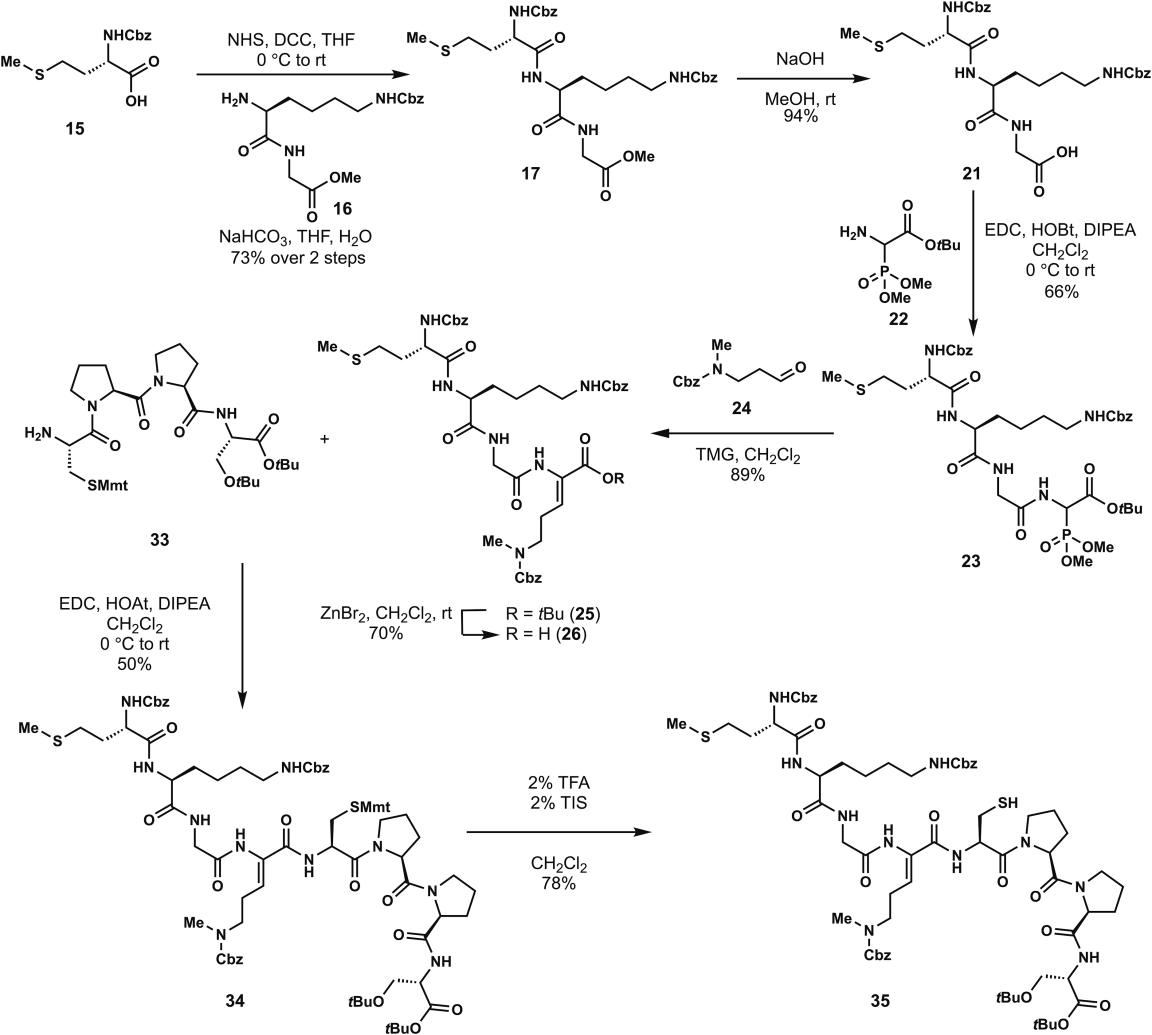
First-Generation Approach toward Enteropeptin A

**Scheme 3:**
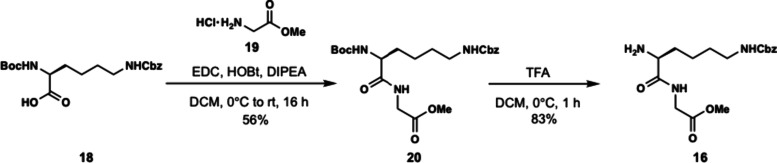
Synthesis of Dipeptide **16**

The C-terminal fragment of enteropeptin A was prepared starting with commercially available Fmoc-protected double proline (Fmoc-Pro-Pro-OH, **27**) which was coupled to H-Ser­(*t*Bu)-O*t*Bu hydrochloride acid salt (**28**) to produce tripeptide **29** (Scheme 4).^31^ Fmoc deprotection occurred smoothly to produce amine **30** in 75% yield. The moderate yield for this deprotection step was proposed to be due to the water solubility of the amine product. Therefore, we next examined the one pot deprotection coupling approach utilized in Boger’s synthesis of streptide.^35^ Fmoc-protected tripeptide **29** was subjected to one equivalent of 1,8-diazabicycloundec-7-ene (DBU) in dichloromethane at 0 °C for 90 min. After complete consumption of the starting material was determined by TLC analysis, Fmoc-Cys­(Mmt)–OH (**31**), HOAt and EDC were added directly to the reaction to produce tetrapeptide **32** in 79% yield directly from **29**. Treatment of **32** with DBU in dichloromethane unveiled the N-terminal amine to afford tetrapeptide **33** in quantitative yield.

**Scheme 4:**
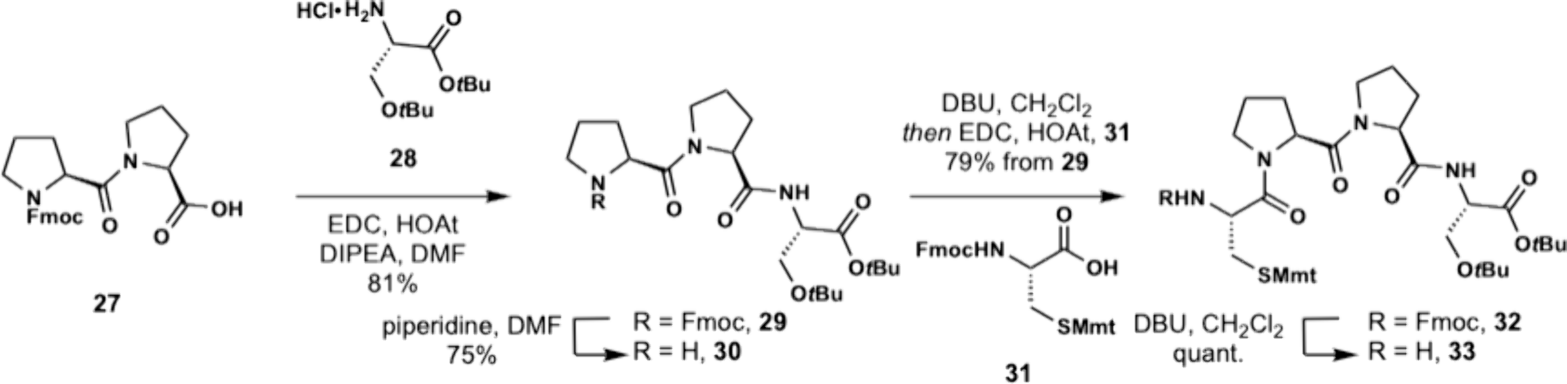
Synthesis of Tetrapeptide **33**

The two peptide fragments **26** and **33** were coupled using EDC, HOAt and Hünig’s base to afford octapeptide **34** containing the enteropeptin A sequence. The 4-methoxytrityl group was selectively deprotected with 2% TFA and 2% triisopropylsilane (TIPS) in CH_2_Cl_2_ to afford thiol **35** in 78% yield (Scheme 2).^36^

With thiol **35** in hand, we were poised to attempt the key step of our synthesis. Thiol **35** was subjected to dithiophosphoric acid (dtPa) **36** and acetonitrile at room temperature which resulted in no reaction (Table 1). Previously, we found that trisubstituted Dhaas required fluorobenzene at elevated temperature to enable Markovnikov hydrothiolation.^18^ Subjecting thiol **35** to dtPa **36** (10 mol %) and fluorobenzene at 90 °C also resulted in no observable formation of thiomorpholine **37**. Thiol **35** was then subjected to microwave irradiation (MWI, 150 °C) with catalyst **36** in trifluorotoluene and resulted in a complex mixture. At this point, we suspected that the peptide containing multiple Lewis basic amides could be inactivating the catalyst. Increasing the catalyst loading to 50 mol % also resulted in a complex mixture without any trace of the desired product (Table 1). Although this strategy was unsuccessful, the stability of thiol **35** was particularly encouraging in that it did not undergo spontaneous conjugate addition. Thiols are well-known to undergo polar or radical addition to dehydroamino acids.^37,38^ In this case, the conjugate addition of thiol **35** appears to be unfavorable due to geometric constraints of the peptide and how β-thiol addition necessitates a 7-endo trig cyclization.^39^ Thus, it was conceivable that a different peptide subjected to these conditions could allow for the desired six-membered thiomorpholine formation *vide infra.*

**Table 1:**
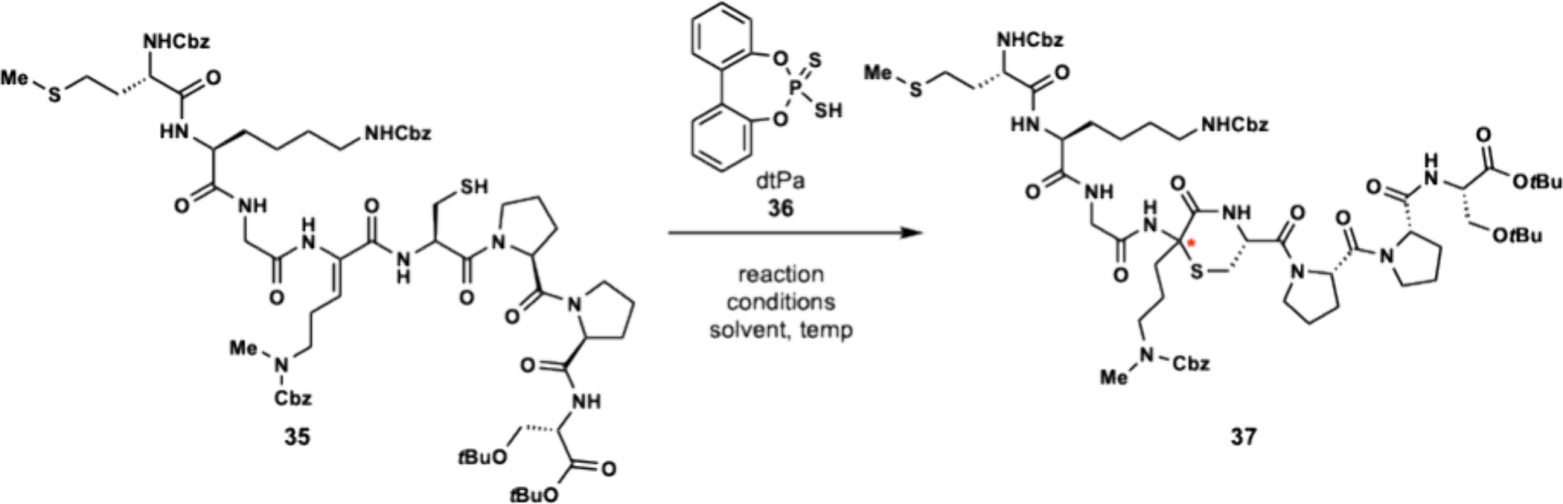
Late-Stage Cyclization Attempts on Peptide **35**

entry	catalyst	conditions	result
1	dtPa (10 mol %)	MeCN, rt	no reaction
2	dtPa (10 mol %)	PhF, 90 °C	no reaction
3	dtPa (10 mol %)	CF_3_Ph, 150 °C (MWI)	complex mixture
4	dtPa (50 mol %)	PhF, 90 °C	complex mixture

### Second-Generation Approach toward Enteropeptin A by a Thiomorpholine Annulation Strategy

In the second-generation approach, the thiomorpholine ring (**38**) was envisioned to be formed by annulation of dehydroamino acid **39** with cysteine **40** containing an unprotected amine and thiol (Scheme 5). The intermolecular hydrothiolation would occur to form the thioaminoketal **41**, and under these conditions spontaneous cyclization of the amine onto the methyl ester of the Dhaa would form the thiomorpholine in a single step. In this approach, smaller peptides were used as model substrates for the annulation reaction which simplified the analysis of the reaction and limited the number of amides in the starting materials to avoid catalyst deactivation. Dhaa **42** and Cys **43** were subjected to dtPa **36** in fluorobenzene at 90 °C and resulted in no reaction (Scheme 6). We suspected the lack of reactivity in this reaction was due to the amine deprotonating the dithiophosphoric acid catalyst preventing it from activating the Dhaa. As such, we then experimented with the hydrochloride acid salt of cysteine where the amine is already protonated. The Cys hydrochloride salt **44** was completely insoluble in PhF even at higher temperature, and no reaction was observed.

**Scheme 5:**
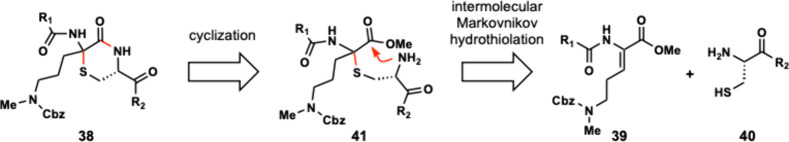
Second-Generation Retrosynthesis of Enteropeptin A by Thiomorpholine Annulation

**Scheme 6:**
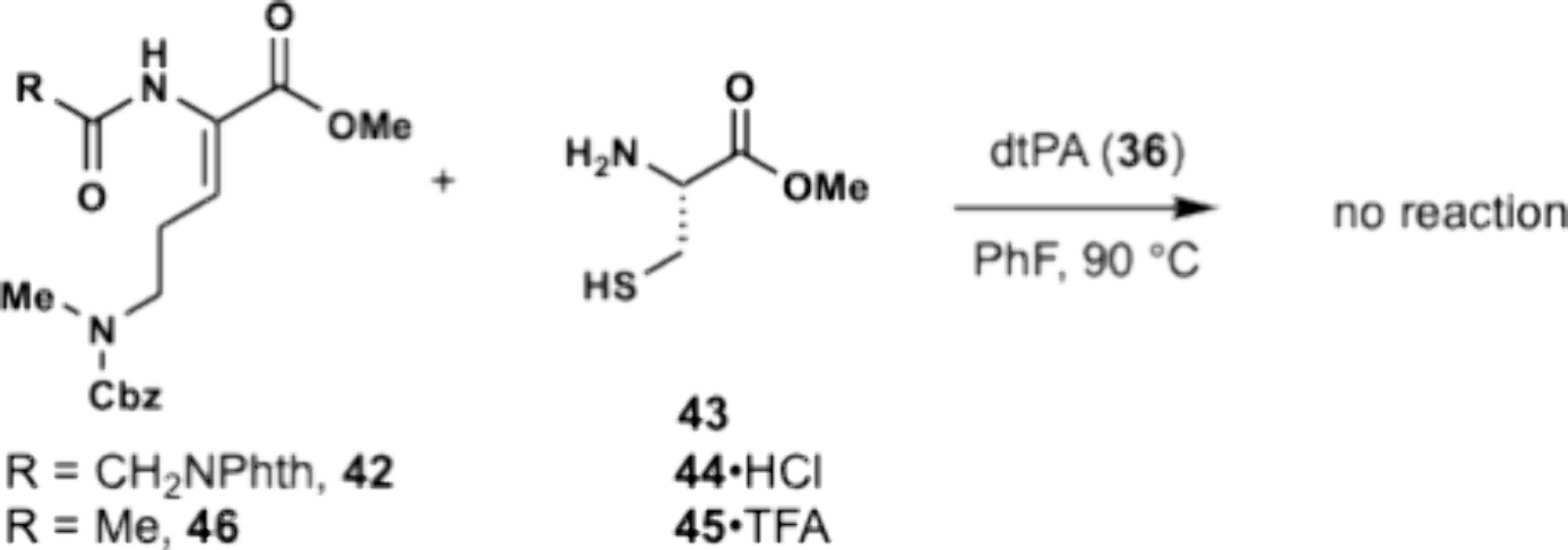
Attempted Thiomorpholine Annulation

The reaction was then attempted between Dhaa **42** and Cys trifluoroacetic acid salt **45**, which had improved solubility in PhF, but still no reaction was observed. A dehydroamino acid containing an N-terminal acetyl group (**46**) was examined as alternative substrate for thiomorpholine annulation. Dhaa **46** was obtained in 92% yield by a HWE reaction with aldehyde **24** and phosphonoglycine **47** (Scheme 7). Dhaa **46** also did not react with cysteine **43** or its protonated salts **44** or **45** under hydrothiolation conditions.

**Scheme 7:**
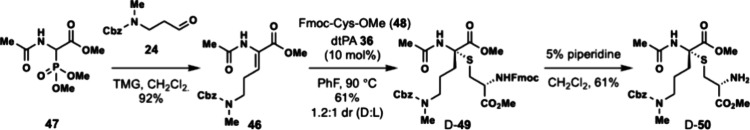
Synthesis of Thioaminoketal D-**50**

Undeterred by these results, we next examined the stepwise formation of the thiomorpholine ring by intermolecular Markovnikov hydrothiolation with a Fmoc-protected cysteine followed by a proposed amine deprotection and spontaneous amidative cyclization to form the six-membered ring. Dhaa **46** was subjected to Markovnikov hydrothiolation with Fmoc-Cys-OMe (**48**) to afford thioaminoketal **49** as a 1.2:1 ratio of diastereomers (D:L). The reaction occurred with slight preference for the D-thioaminoketal diastereomer which is found in the enteropeptins. Fortunately, the diastereomers (L-**49** and D-**49**) were easily separated by silica gel chromatography. Using D-**49** which contained the correct stereochemical configuration, the Fmoc group was removed using piperidine to form amine D-**50** in 61% yield. With amine D-**50** in hand, we then examined different conditions for amidative cyclization between the amine and the methyl ester to form thiomorpholine **51** (Table 2). Under acidic conditions, none of the desired product was observed. This was consistently observed using both Bro̷nsted acid catalysts, such as trifluoroacetic acid, or Lewis acids (Table 2, entry 1–4). Meanwhile under basic conditions, D-**50** was not stable likely due to fragmentation of the thioaminoketal. Previous work by Malins have shown that thioaminoketal containing peptides are not stable to basic conditions.^16^ Under bases like DBU or KO*t*Bu, extensive decomposition of the starting material was observed. When D-**50** was subjected to potassium carbonate in methanol (K_2_CO_3_/MeOH) formation of desired thiomorpholine **51** was obtained in 10% yield with the formation of a major byproduct, aminoketal **52**. The byproduct could be formed by elimination of the Cys to form an iminium followed by addition of methanol solvent. We then examined polar aprotic solvents such as THF or DMF which cannot form the aminoketal byproduct. Unfortunately, these conditions did not form the desired product. Despite our efforts, we were unsuccessful in improving the amide-bond forming reaction. Given the difficulty in optimization of the amidative cyclization, we revisited our initial strategy to form the peptide bond first followed by thiol cyclization. However, rather than performing the cyclization at the stage of the full-length peptide, we planned to investigate the thiol cyclization on simpler peptides early in the synthesis.

**Table 2:**
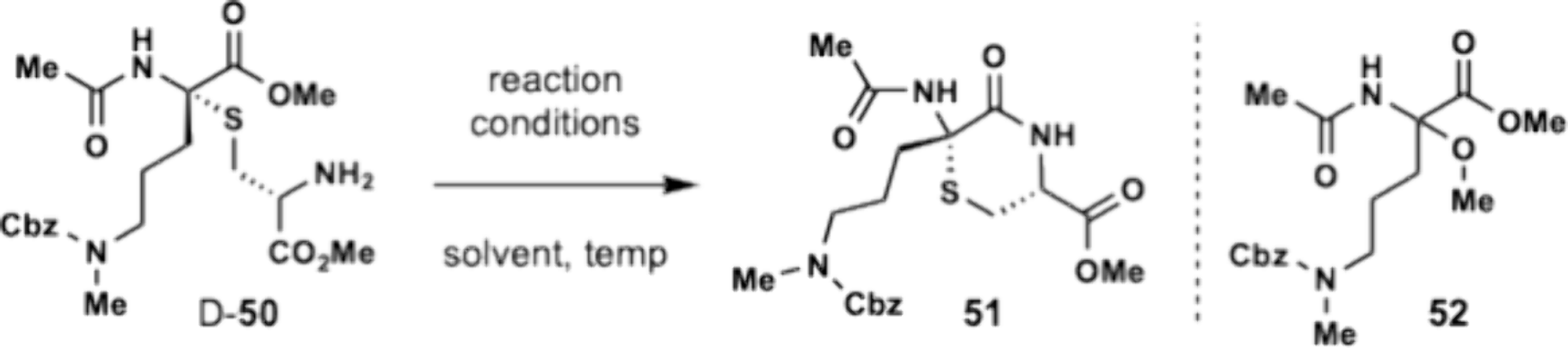
Optimization of the Amidative Cyclization

entry	reagent	solvent, temperature	yield (%)
1	TFA	CH_2_Cl_2_, rt	0
2	BF_3_·OEt_2_	CH_2_Cl_2_, rt	0
3	AlCl_3_	CH_2_Cl_2_, rt	0
4	MgBr_2_	THF, rt	0
5	DBU	CH_3_CN	0
6	KO*t*Bu	THF, rt	0
7	K_2_CO_3_	DMF, 110 °C	0
8	K_2_CO_3_	THF, 70 °C	0
9	K_2_CO_3_	MeOH, rt	10 (**51**), 45 (**52**)

### Third Generation: Early-Stage Cyclization

In our third and final iteration of our synthetic strategy, we examined early stage formation of the thiomorpholine ring by forming the amide bond first followed by thiol cyclization. Realizing that our previous unsuccessful attempts at intramolecular hydrothiolation could have been due to catalyst inactivation by the complex peptide starting material, we wondered if a shorter peptide could be amenable to thiol cyclization. Moreover, an early stage cyclization strategy would allow for a modular and unified synthesis of the enteropeptins through sequential coupling of different N- and C-terminal peptide fragments. Thus, we targeted cyclization of the central GOC tripeptide sequence which is common to all enteropeptins. Methyl ester **42** was converted to acid **53** by a modified Krapcho demethylation using lithium iodide^40^ followed by EDC coupling with S-trityl l-cysteine methyl ester (**54**) to afford tripeptide **55** in 34% yield over two steps. The trityl group was removed using TFA and triisopropylsilane to unveil thiol **56** in 92% yield. Thiol **56** was subjected to microwave irradiation in the presence of dithiophosphoric acid **36** as catalyst and trifluorotoluene at 150 °C to form thiomorpholine **57** in 61% yield (Scheme 8). Unfortunately, the reaction occurred to give exclusively the incorrect stereoisomer as the cyclization resulted in formation of the L-thioaminoketal while the enteropeptins possess the opposite D-configuration.

**Scheme 8:**
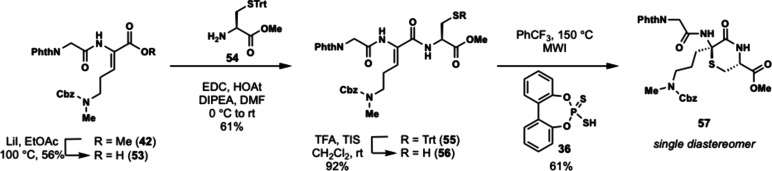
Synthesis of Thiomorpholine **57**

To synthesize enteropeptin A, we then started the synthesis using d-cysteine which would result in formation of the D-thioaminoketal (Scheme 9). Subsequent epimerization of the cysteine α-carbon would form the desired thiomorpholine for enteropeptin synthesis. Executing this strategy, acid **53** was subjected to EDC coupling with d-cysteine *ent*-**54** to afford tripeptide *ent*-**55**. Thiol deprotection produces *ent*-**56** which is subjected to dithiophosphoric acid catalyzed Markovnikov hydrothiolation to afford the D-thioaminoketal. Epimerization of the α-carbon with DBU occurred in 72% yield to afford **58** as a 1.5:1 mixture of diastereomers favoring the desired L configuration of cysteine. Krapcho demethylation resulted in demethylation of the methyl ester of **58** to afford acid **59**. The C-terminal acid of **59** was coupled with dipeptide **30** to afford hexapeptide **60** in 76% yield over two steps. Compound **60** was subjected to phthaloyl deprotection with ethylenediamine to afford amine **61**. The N-terminal amine of **61** was coupled with the dipeptide **62** to afford the octapeptide **63** containing the enteropeptin A sequence. Global deprotection was accomplished using trimethylsilyl bromide in trifluoroacetic acid to produce enteropeptin A (**4a**).^41,42^ Thioanisole proved essential to scavenge the highly reactive carbocation in the deprotection step to prevent undesired benzylation of the methionine thioether. This work constitutes a 14-step total synthesis of enteropeptin A and the first total synthesis of a sactipeptide.^31^

**Scheme 9:**
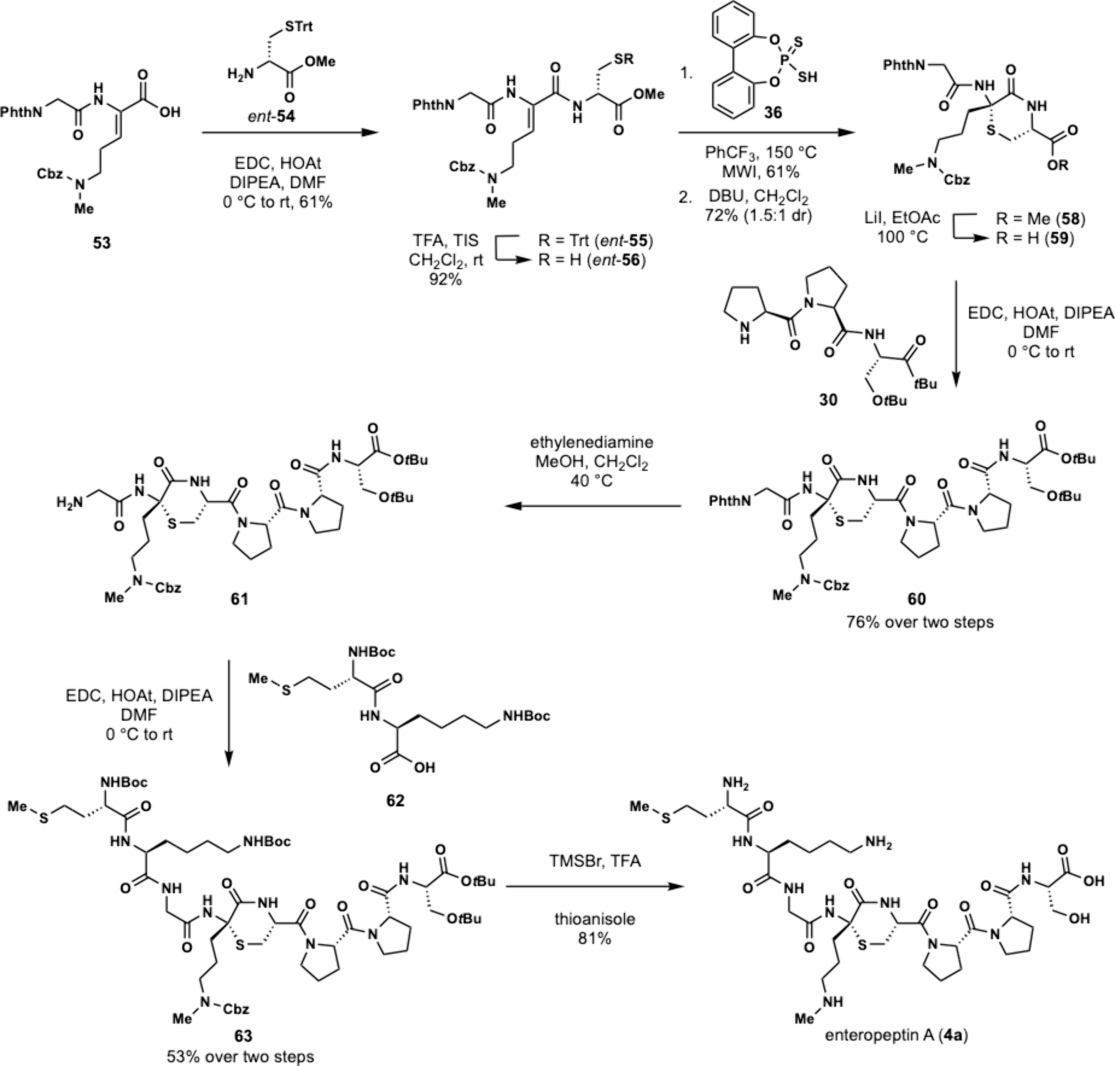
Total Synthesis of Enteropeptin A

Enteropeptin B (**4b**) is a heptapeptide that lacks the C-terminal serine residue when compared to **4a**. The modular synthesis that is achieved by early stage thiol cyclization allowed for the synthesis of enteropeptin B (**4b**) and C (**4c**, Scheme 10). The synthesis of the peptide congeners was initiated utilizing the same intermediates in the synthesis of enteropeptin A. Carboxylic acid **59** was coupled to double proline **64** which allowed for incorporation of the C-terminus of enteropeptin B. Aminolysis of the phthalimide **65** with ethylenediamine occurred smoothly to afford amine **66**. Coupling with the N-terminal dipeptide (**62**) afforded a heptapeptide containing the enteropeptin B sequence in 54% yield over two steps. The heptapeptide was subjected to global deprotection to complete the total synthesis of enteropeptin B (**4b**).

**Scheme 10:**
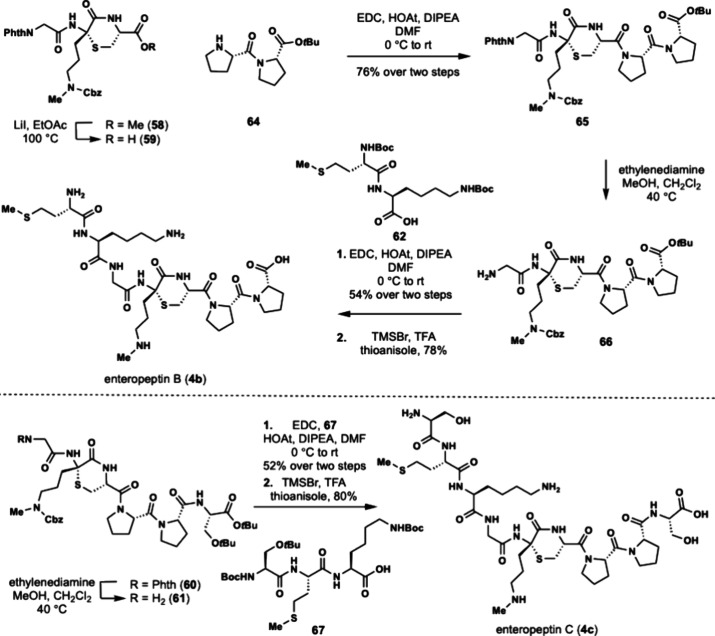
Total Synthesis of Enteropeptin B and C

Finally, we targeted enteropeptin C (**4c**) which is a nonapeptide that contains an extra N-terminal serine. Phthalimide **60** was treated with ethylenediamine to afford amine **61** which was then coupled to the N-terminal tripeptide **67** using EDC, HOAt and DIPEA. The resulting nonapeptide was subjected to global deprotection with TMSBr to afford **4c** in 80% yield (Scheme 10).

The early stage Markovnikov hydrothiolation enabled a modular synthesis compared with our initial strategy. By forming the thiomorpholine ring first, the subsequent amide bond-forming reactions with different N- and C-terminal peptides enabled a unified synthesis of enteropeptins A-C (**4a**-**4c**).

The key step in our unified synthesis of enteropeptins is a stereoselective Markovnikov hydrothiolation reaction that forms the thiomorpholine ring with exclusive diastereoselectivity. To further understand the origins of stereoselective peptide cyclization, compound **58** containing the D-thioaminoketal was resubjected to the cyclization conditions with dtPa **36**. Interestingly, no reaction was observed, and no epimerization of the thioaminoketal to the l-isomer **57** occurred (Figure 4a). This result indicates that the cyclization reaction is under kinetic control since the thioaminoketal does not epimerize or equilibrate under the cyclization conditions. Therefore, the diastereoselectivity of the peptide cyclization is due to the difference in transition state energies where cyclization of **56** to form **57** is favored compared to the transition state leading to **58**. A transition state model is proposed where the iminium ion is formed from dithiophosphoric acid catalyzed activation of the Dhaa. The iminium ion is proposed to be coplanar and adopt an *s*-trans configuration with the amide carbonyl for conjugation and for minimization of dipole moment.^43,44^ Thiol addition is proposed to occur through a six-membered boat-like transition state where thiol addition from the front face of the iminium ion leading to **57** is favored due to placing the hydrogen of Cys in the flagpole position (Figure 4b). Thiol addition from the back face of the iminium ion leading to **58** is disfavored, because the methyl ester would be in the flagpole position and result in a steric clash with the iminium ion. While chairlike transition states are often favored over boat-like transition states for six-membered ring formation, amide induced planarity from the peptide results in additional conformational restrictions. The coplanar iminium ion with the amide carbonyl results in three consecutive *sp^2^*-hybridized atoms in the six-membered transition state making it sterically prohibitive for the thiol methylene of the Cys side chain to cyclize through a half-chair transition state. For these reasons, we propose that the cyclization reaction to form the thiomorpholine ring in our enteropeptin synthesis occurs through a boat-like transition state (Figure 4b).

**Figure 4:**
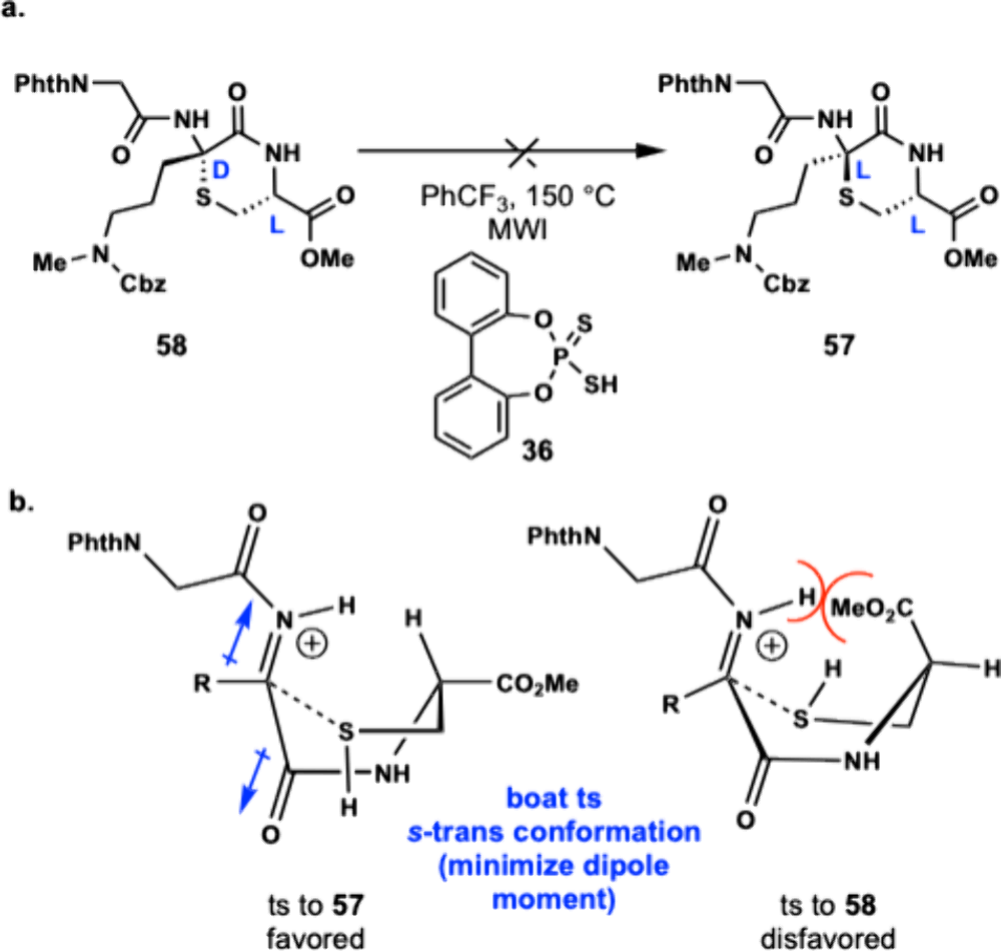
(a) Thioaminoketal epimerization experiment. (b) Proposed transition state for stereoselective peptide cyclization.

## Discussion

In summary, the unified total synthesis of the enteropeptin sactipeptides was accomplished through early stage formation of the thiomorpholine ring and amidative coupling of peptides of similar molecular complexity. Such strategies have been particularly successful in the preparation of analogs of natural products for the discovery of new antibiotics.^45–48^ Key to the success of our synthesis was the development of a Markovnikov hydrothiolation of Dhaa which forms the carbon–sulfur bond found in sactipeptides. For this reaction, the dithiophosphoric acid was essential for achieving the cyclization to form the thiomorpholine ring of the enteropeptins at an early stage in the synthesis. In conclusion, we have completed the first total synthesis of a sactipeptide in the synthesis of the enteropeptin A and assigned the stereochemical configuration of the thioaminoketal to be D-configured. Given that many new RiPPs are being discovered through advances in gene sequencing, it is expected that the number of isolated sactipeptide natural products will only continue to grow.^49^ Therefore, the development of new synthetic methods to access cross-links found in RiPP natural products will be highly enabling for the synthesis of newly discovered peptide natural products.

## Experimental Section

### General Procedures

Unless otherwise stated, all reactions were performed in oven-dried or flame-dried glassware under an atmosphere of dry nitrogen. Dry tetrahydrofuran (THF), dichloromethane, methanol, dimethylformamide (DMF), and acetonitrile were obtained by passing these previously degassed solvents through activated alumina columns. Anhydrous *a,a,a-*trifluorotoluene was used directly from Aldrich Sure/Seal bottles. Fluorobenzene was distilled from calcium hydride before use. Amines were distilled from calcium hydride before use. Reactions were monitored by thin layer chromatography (TLC) on Silicycle Siliaplate TLC plates (250 μm thickness, 60 Å porosity, F-254 indicator) and visualized by ultraviolet irradiation and staining with *p*-anisaldehyde, phosphomolybdic acid, or potassium permanganate developing agents. Volatile solvents were removed under reduced pressure using a rotary evaporator. Flash column chromatography was performed using Silicycle F60 silica gel (60Å, 230–400 mesh, 40–63 μm). Proton nuclear magnetic resonance (^1^H NMR), carbon nuclear magnetic resonance (^13^C NMR), and phosphorus nuclear magnetic resonance (^31^P NMR) spectra were recorded on Varian AVQ-400 and Bruker Avance NEO 400 MHz spectrometers. NMR spectra were recorded at 400 MHz for ^1^H, 100 MHz for ^13^C, and 162 MHz for ^31^P, using CDCl_3_ (^1^H, 7.26 ppm; ^13^C, 77.16 ppm), CD_3_OD (^1^H, 3.31 ppm; ^13^C, 49.00 ppm), D_2_O (^1^H, 4.79 ppm) as internal standard. The following abbreviations were used to explain the multiplicities: s = singlet, bs = broad singlet, d = doublet, t = triplet, q = quartet, dd = doublet of doublets, dt = doublet of triplets, td = triplet of doublets, m = multiplet, coupling constant (Hz), and integration. Melting points were determined using Mel-Temp apparatus. Infrared (IR) spectra were recorded on a Nicolet 380 FT-IR spectrometer. High-resolution mass spectra (HRMS) were obtained by a Bruker TIMS time-of-flight (tof) pro at the mass spectrometry facility at Brandeis University. A CEM Mars 6 Microwave Synthesizer instrument purchased from CEM Corporation was used for microwave irradiation. Microwave synthesis was conducted in microwave reactor with 20 mL sealed microwave reaction vessels (GlassChem 20 vessel). The reaction temperature is monitored by the MTS-300 temperature fiber optic probe from CEM Corporation. Compound **15** was purchased from Tokyo Chemical Industry (TCI). Enteropeptin A, B, and C were prepared using previously reported procedures. Compound **22** was prepared using previously reported procedures by Schmidt.^32^ Compound **4a**, **24**, **29**, **30**, **42**, **53**, **55**, *ent*-**55**, **56**, *ent-* **56**, **57**, **58**, **59**, **60**, **61**, **62**, and **63** were prepared using previously reported procedures in ref 30.^31^ Compound **4b**, **4c**, **64**, **65**, **66**, and **67** were prepared using previously reported procedures in ref 18.^18^ Structural assignments were made with additional information from gCOSY and gTOCSY experiments.
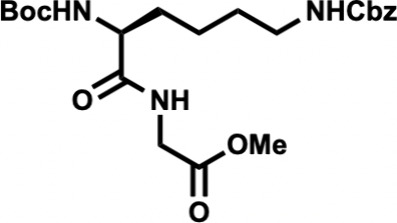


**Dipeptide 20**. A 250 mL flame-dried round-bottom flask equipped with a magnetic stir bar was charged with Boc-Lys­(Cbz)–OH (**18**, 2.00 g, 5.30 mmol, 1.0 equiv) and HOBt (0.70 g, 5.30 mmol, 1.0 equiv). The reaction vessel was evacuated and backfilled with nitrogen gas three times. Anhydrous CH_2_Cl_2_ (40 mL) was added to the reaction. Then, the reaction vessel was cooled to 0 °C followed by the addition of EDC (0.90 mL, 5.30 mmol, 1.0 equiv). The reaction mixture was kept at this temperature for 30 min. At this point, glycine methyl ester hydrochloride salt (**19**, 0.70 g, 5.30 mmol, 1.0 equiv) was added as a solid followed by the dropwise addition of Hünig’s base (iPr_2_NEt, 2.30 mL, 13.2 mmol, 2.5 equiv). The reaction mixture was left to warm to room temperature. After 16 h, the reaction mixture was quenched with 1 M HCl (20 mL) and then extracted with CH_2_Cl_2_ (3 × 50 mL). The combined organic layer was washed with brine and dried over with anhydrous Na_2_SO_4_, filtered and concentrated under reduced pressure. The resulting crude mixture was purified by silica gel column chromatography (50–90% EtOAc in Hexanes) to provide dipeptide **20** (1.35 g, 56% yield) as a white solid. **M.p.**: 78–79 °C. ^**1**^ **H NMR** (400 MHz, CDCl_3_): δ 7.32–7.16 (m, 5H), 7.12 (t, *J* = 5.6 Hz, 1H), 5.44 (d, *J* = 8.1 Hz, 1H), 5.24 (t, *J* = 5.0 Hz, 1H), 5.00 (s, 2H), 4.12 (dd, *J* = 7.5, 7.4 Hz, 1H), 3.98 (dd, *J* = 18.1, 5.7 Hz, 2H), 3.86 (dd, J = 18.1, 5.3 Hz, 1H), 3.61 (s, 3H), 3.10 (dd, *J* = 6.6, 6.5 Hz, 2H), 1.75 (dt, *J* = 13.9, 7.2 Hz, 1H), 1.58 (dt, *J* = 14.4, 7.6 Hz, 1H), 1.49–1.39 (m, 2H), 1.35 (s, 9H), 1.37–1.32 (m, 2H). ^**13**^ **C­{** ^**1**^ **H} NMR** (100 MHz, CDCl_3_): δ 172.8, 170.3, 156.7, 155.9, 136.6, 128.5, 128.1, 128.1, 80.0, 66.6, 54.1, 52.3, 41.0, 40.4, 32.0, 29.3, 28.3, 22.3. **FTIR** (thin film) cm^–1^: 3319, 2941, 1731, 1690, 1676, 1649, 1546, 1528. **HRMS (ESI)** (*m*/*z*): Calc.’d for C_22_H_34_N_3_O_7_ [M + H]^+^: 452.2391, found 452.2381.
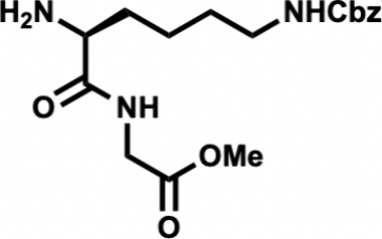


**Dipeptide 16**. A 500 mL flame-dried round-bottom flask equipped with a magnetic stir bar was charged with Boc-Lys­(Z)-Gly-OMe (**20**, 10.4 g, 23 mmol, 1.0 equiv). The reaction vessel was evacuated and backfilled with nitrogen gas three times. Anhydrous CH_2_Cl_2_ (100 mL) was introduced via cannula followed by the addition of trifluoroacetic acid (100 mL). After 1 h, the reaction mixture was concentrated under reduced pressure. The resulting solution was further basified with saturated NaHCO_3_ (150 mL), extracted with EtOAc (200 mL x 3), and washed with brine (100 mL). The combined organic layer was dried over Na_2_SO_4_ and concentrated under reduced pressure to afford dipeptide **16** as a white solid (6.7 g, 83% yield). The crude solid was used directly in the next step without further purification. **M.p.**: 160–161 °C. ^**1**^ **H NMR** (400 MHz, CDCl_3_): δ 8.16 (s, 1H), 7.36–7.18 (m, 5H), 5.42–5.17 (m, 3H), 5.00 (s, 2H), 4.00–3.86 (m, 2H), 3.77 (t, J = 6.8 Hz, 1H), 3.61 (s, 3H), 3.21–2.99 (m, 2H), 1.86–1.73 (m, 1H), 1.73–1.61 (m, 1H), 1.51–1.29 (m, 4H). ^**13**^ **C­{** ^**1**^ **H} NMR** (100 MHz, CDCl_3_): δ 172.7, 170.5, 156.8, 136.7, 128.6, 128.1, 128.0, 66.6, 54.1, 52.4, 41.0, 40.5, 32.7, 29.4, 22.0. **FTIR** (thin film) cm^–1^: 3288, 3060, 2947, 1683, 1634, 1531, 1468, 1439. **HRMS (ESI)** (*m*/*z*): Calc.’d for C_17_H_26_N_3_O_5_ [M + H]^+^: 352.1867, found 352.1891.
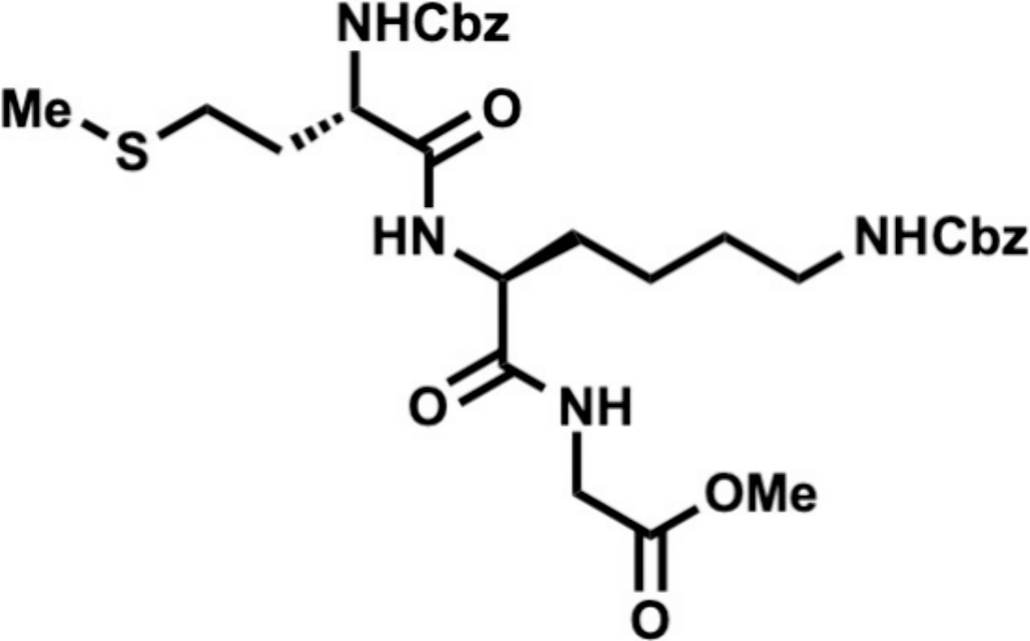


**Tripeptide 17**. A 500 mL flame-dried round-bottom flask equipped with a magnetic stir bar was charged with Z-Met-OH (**15**, 2.83 g, 10 mmol, 1.0 equiv) and N-hydroxysuccinimide (1.38 g, 12 mmol, 1.2 equiv). The reaction vessel was evacuated and backfilled with nitrogen gas three times. Anhydrous THF (60 mL) was introduced via cannula. Then, the reaction vessel was cooled to 0 °C and followed by the addition of DCC (2.16 g, 10.5 mmol, 1.05 equiv) as a solid. The reaction mixture was kept at this temperature for 30 min and slowly warmed to room temperature. After 16 h, the reaction mixture was filtered through a fritted glass funnel to remove DCU. The filtrate was concentrated under reduced pressure and used directly in the next step without further purification.

The crude residue was then charged with H-Lys­(Z)-Gly-OMe (**16**, 3.51 g, 10 mmol, 1.0 equiv). The reaction vessel was evacuated and backfilled with nitrogen gas three times. Anhydrous THF (60 mL) was introduced via cannula. Then, the reaction mixture was cooled to 0 °C and followed by the addition of NaHCO_3_ (3.36 g, 40 mmol, 4 equiv) dissolved in water (60 mL). After 30 min, the reaction was slowly warmed to room temperature and stirred for 16 h. The reaction mixture was then washed with 1 M HCl (100 mL), extracted with EtOAc (200 mL x 3) and washed with brine (100 mL). The combined organic layer was dried over Na_2_SO_4_ and concentrated under reduced pressure. The resulting crude residue was purified by crystallization using EtOAc to afford the tripeptide **17** as a white solid (4.50 g, 73% yield). **M.p.**: 163–164 °C. ^**1**^ **H NMR** (400 MHz, d_6_-DMSO): δ 8.36 (t, J = 5.8 Hz, 1H), 7.92 (d, J = 8.1 Hz, 1H), 7.50 (d, J = 8.1 Hz, 1H), 7.42–7.25 (m, 10H), 7.19 (t, J = 5.7 Hz, 1H), 5.02 (s, 2H), 5.00 (s, 2H), 4.27 (dd, J = 7.5 Hz, 1H), 4.11 (dt, J = 8.7, 4.9 Hz, 1H), 3.89 (dd, J = 17.4, 5.8 Hz, 1H), 3.80 (dd, J = 17.4, 5.7 Hz, 1H), 3.61 (s, 3H), 2.97 (dd, J = 6.7 Hz, 2H), 2.45 (t, J = 7.7 Hz, 2H), 2.02 (s, 3H), 1.95–1.82 (m, 1H), 1.82–1.71 (m, 1H), 1.68–1.58 (m, 1H), 1.58–1.46 (m, 1H), 1.45–1.35 (m, 2H), 1.35–1.25 (m, 2H). ^**13**^ **C­{** ^**1**^ **H} NMR** (100 MHz, d_6_-DMSO): δ (ppm) 172.1, 171.3, 170.2, 156.1, 156.0, 137.3, 137.0, 128.3, 127.8, 127.7, 127.7, 65.4, 65.1, 53.9, 52.2, 51.7, 40.5, 40.2, 33.4, 31.8, 29.7, 29.1, 22.4, 14.6. Two aromatic carbon signals are missing. **FTIR** (thin film) cm^–1^: 3288, 3063, 2936, 1735, 1685, 1633, 1530, 1454. **HRMS (ESI)** (*m*/*z*): Calc.’d for C_30_H_41_N_4_O_8_S [M + H]^+^: 617.2640, found 617.2604.
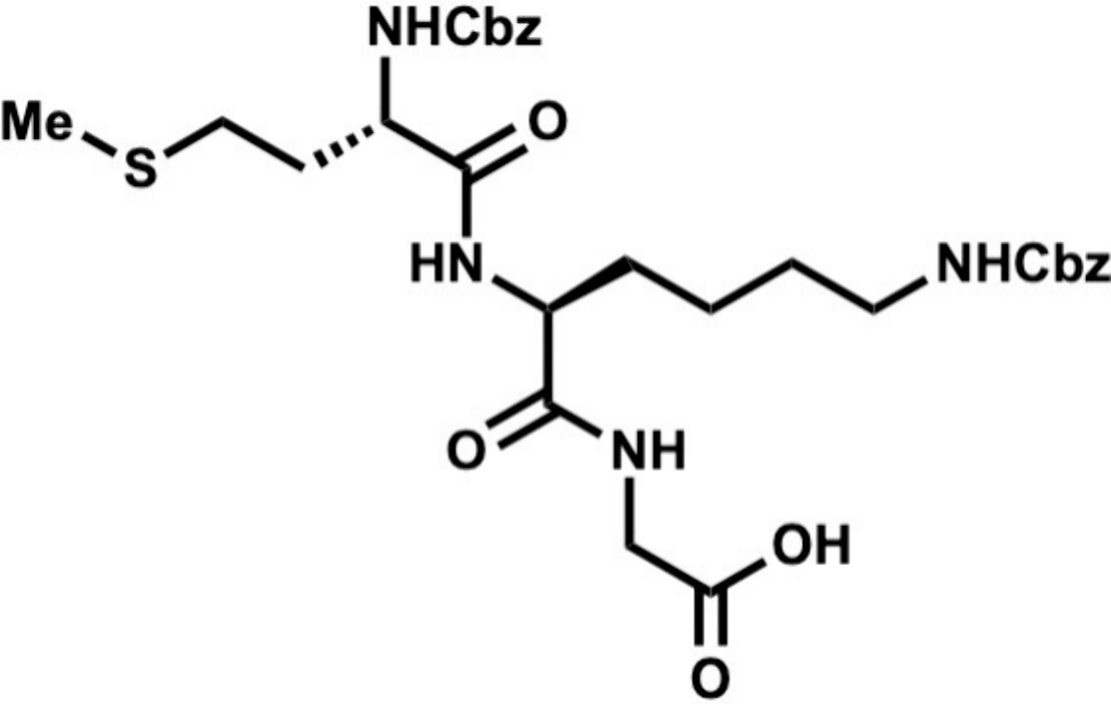


**Acid 21**. A 250 mL round-bottom flask equipped with a magnetic stir bar was charged with tripeptide **17** (0.92 g, 1.5 mmol, 1.0 equiv). Methanol (40 mL) was added to the reaction vessel followed by the addition of sodium hydroxide (0.24 g, 6 mmol, 4.0 equiv) in deionized water (20 mL). After 1 h, the reaction mixture was acidified with 1 M HCl, extracted with EtOAc (80 mL x 4), and washed with brine (50 mL). The combined organic layers were dried over Na_2_SO_4_ and concentrated under reduced pressure. The crude residue was purified by silica gel column chromatography (1% to 6% MeOH in CH_2_Cl_2_) to afford acid **21** as a white solid (0.85 g, 94% yield). **M.p.**: 149–150 °C. ^**1**^ **H NMR** (400 MHz, d_6_-DMSO) δ 8.18 (t, J = 5.9 Hz, 1H), 7.91 (d, J = 8.3 Hz, 1H), 7.50 (d, J = 8.1 Hz, 1H), 7.39–7.26 (m, 10H), 7.19 (t, J = 5.8 Hz, 1H), 5.02 (s, 2H), 4.99 (s, 2H), 4.26 (ddd, J = 8.6, 5.1 Hz, 1H), 4.10 (ddd, J = 8.5, 4.6 Hz, 1H), 3.78 (dd, J = 17.5, 5.8 Hz, 1H), 3.70 (dd, J = 17.5, 5.8 Hz, 1H), 2.95 (dd, J = 6.5 Hz, 2H), 2.48–2.38 (m, 2H), 2.02 (s, 3H), 1.94–1.83 (m, 1H), 1.83–1.71 (m, 1H), 1.71–1.59 (m, 1H), 1.57–1.45 (m, 1H), 1.43–1.32 (m, 2H), 1.32–1.18 (m, 2H). ^**13**^ **C­{** ^**1**^ **H} NMR** (100 MHz, d_6_-DMSO) δ 171.9, 171.3, 171.2, 156.1, 156.0, 137.3, 137.0, 128.4, 127.8, 127.8, 127.7, 65.5, 65.2, 54.0, 52.3, 40.7, 40.3, 31.9, 31.7, 29.7, 29.2, 22.5, 14.7. Two aromatic carbon signals are missing. **FTIR** (thin film) cm^–1^: 3289, 3067, 2919, 1687, 1634, 1532, 1454, 1440. **HRMS** (ESI) (*m*/*z*): Calc.’d for C_29_H_39_N_4_O_8_S [M + H]^+^: 603.2483, found 603.2503.
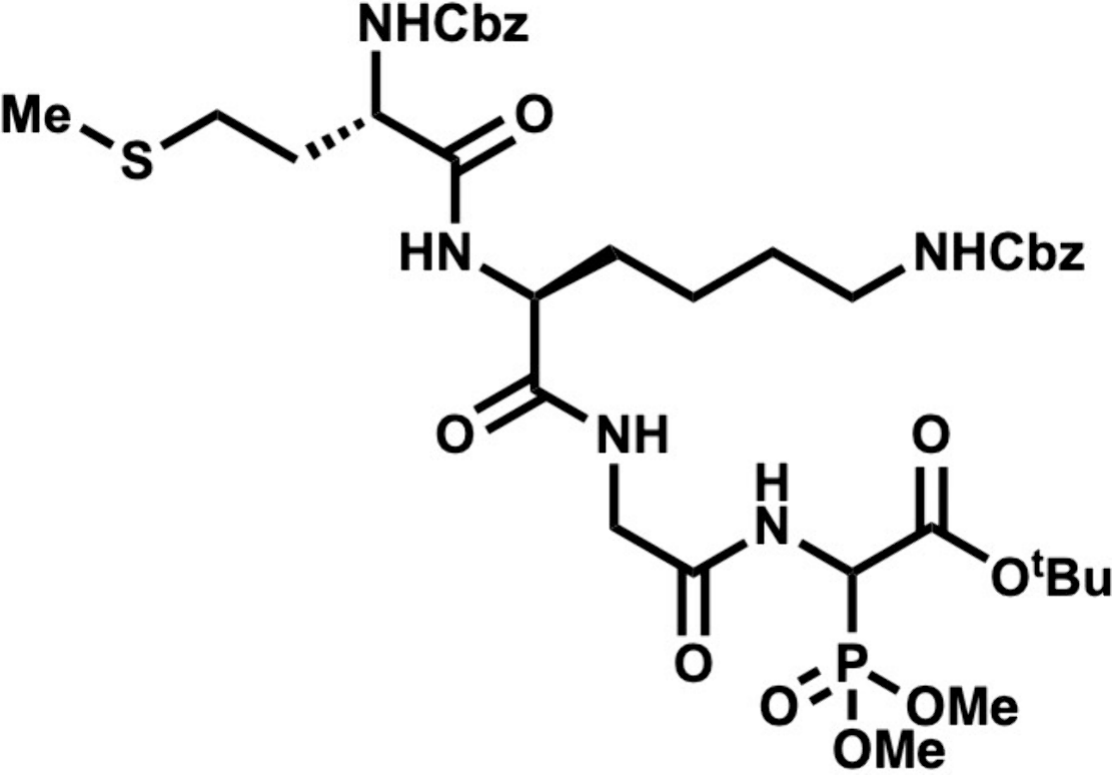


**Phosphonate 23**. A 250 mL flame-dried round-bottom flask equipped with a magnetic stir bar was charged with acid **21** (0.85 g, 1.4 mmol, 1.0 equiv) and HOBt (283 mg, 2.1 mmol, 1.5 equiv). The reaction vessel was evacuated and backfilled with nitrogen gas three times. At this point, the reaction vessel was cooled to 0 °C, and CH_2_Cl_2_ (20 mL) was added to the reaction vessel followed by the addition of EDC (0.36 mL, 2.07 mmol, 1.5 equiv). The reaction mixture was kept at this temperature for 30 min. Then, amine **22** (0.5 g, 2.1 mmol, 1.5 equiv) was added as a solution in dichloromethane (10 mL) followed by the dropwise addition of Hünig’s base (iPr_2_NEt, 0.6 mL, 3.5 mmol, 2.5 equiv). The reaction mixture was left to slowly warm to room temperature. After 16 h, the solution was washed with 10 wt % citric acid (50 mL), extracted with dichloromethane (50 mL x 3) and washed with brine (50 mL). The combined organic layers were dried over Na_2_SO_4_ and concentrated in vacuo. The crude residue was purified by silica gel column chromatography (1% to 5% MeOH in CH_2_Cl_2_) to afford phosphonate **23** as a white solid (760 mg, 66% yield) as a 1:1 mixture of diastereomers. **M.p.**: 90–91 °C. The NMR spectra are of a mixture of two diastereomers. ^**1**^ **H NMR** (400 MHz, CDCl_3_) 7.75 (dd, J = 48.7 (^3^ *J*_HP_), 8.2 Hz, 1H), 7.63–7.50 (m, 3H), 7.44–7.37 (m, 2H), 7.37–7.28 (m, 20H), 6.26–5.97 (m, 2H), 5.47–5.23 (m, 2H), 5.19–5.00 (m, 10H), 4.57–4.39 (m, 4H), 4.20–3.96 (m, 4H), 3.78 (s, 3H), 3.75 (s, 6H), 3.72 (s, 3H), 3.21–3.05 (m, 4H), 2.58–2.48 (m, 4H), 2.12–2.04 (m, 2H), 2.04 (s, 6H), 2.01–1.90 (m, 2H), 1.88–1.77 (m, 2H), 1.75–1.62 (m, 2H), 1.54–1.46 (m, 4H), 1.45 (s, 18H), 1.41–1.30 (m, 4H). ^**13**^ **C­{** ^**1**^ **H} NMR** (100 MHz, CDCl_3_) δ 172.34, 172.30, 172.09, 172.07, 168.87, 168.82, 168.81, 168.8, 165.23, 165.22, 156.80, 156.76, 156.55, 156.47, 136.9, 136.6, 128.5, 128.2, 128.10, 128.05, 83.88, 83.85, 66.95, 66.57, 54.14, 53.16, 53.07, 51.8, 51.6, 50.3, 50.2, 43.2, 43.1, 40.7, 32.7, 30.1, 29.5, 27.94, 27.93, 22.53, 15.36. Aromatic carbon signals from the two Cbz groups and the two diastereomers are overlapping. ^**31**^ **P NMR** (162 MHz, CDCl_3_) δ 18.9, 18.8. **FTIR** (thin film) cm^–1^: 3300, 2930, 1729, 1686, 1648, 1633, 1528, 1454. **HRMS** (ESI) (*m*/*z*): Calc.’d for C_37_H_55_N_5_O_12_PS [M + H]^+^: 824.3300, found 824.3269.
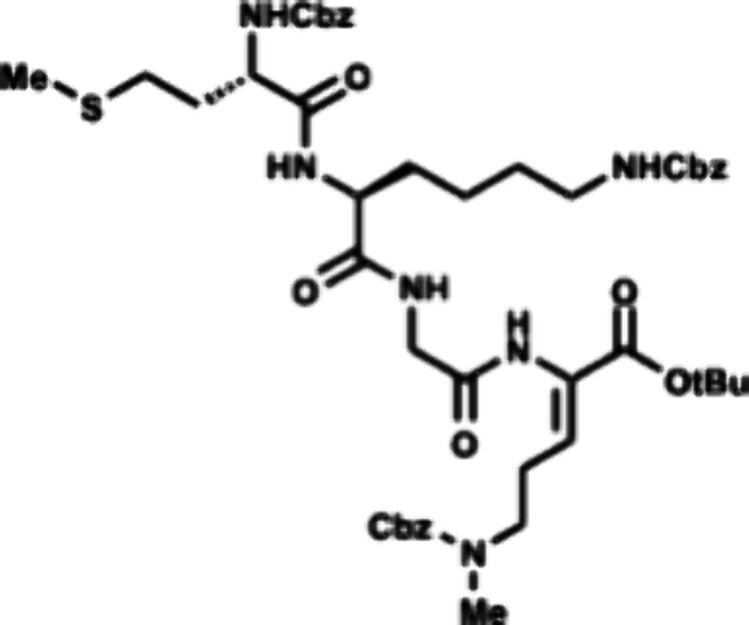


**Tetrapeptide 25**. A flame-dried round-bottom flask was charged with phosphonate **23** (4.9 g, 6 mmol, 1.0 equiv). The reaction vessel was evacuated and backfilled with nitrogen gas and this process was repeated three times. Anhydrous CH_2_Cl_2_ (60 mL) was introduced via cannula. Then, tetramethylguanidine (1.9 mL, 15 mmol, 2.5 equiv) was added to the reaction mixture, followed by the addition of aldehyde **24** (1.6 g, 7.2 mmol, 1.2 equiv) in 60 mL CH_2_Cl_2_. After 15 min, reaction mixture was quenched with 1 M HCl (20 mL) and the reaction mixture was then extracted with CH_2_Cl_2_ (2 × 80 mL). The combined organic layer was washed with brine and dried over with anhydrous Na_2_SO_4_, filtered, and concentrated under reduced pressure. The resulting crude mixture was purified by silica gel column chromatography (80% EtOAc in Hexanes) to provide the tetrapeptide **25** (4.9 g, 89% yield) as a white solid. **M.p.**: 93–94 °C.^1^ **H NMR** (400 MHz, CD_3_OD): δ 7.40–7.23 (m, 15H), 6.56 (t, J = 7.5 Hz, 1H), 5.10 (s, 2H), 5.08–5.02 (m, 4H), 4.31–4.27 (m, 1H), 4.25 (dd, J = 9.0, 5.3 Hz, 1H), 4.06–3.94 (m, 1H), 3.94–3.84 (m, 1H), 3.41 (t, J = 7.0 Hz, 2H), 3.10 (t, J = 6.7 Hz, 2H), 2.90 (s, 3H), 2.60–2.45 (m, 2H), 2.45–2.35 (m, 2H), 2.04 (s, 3H), 1.95–1.80 (m, 2H), 1.78–1.65 (m, 1H), 1.59–1.28 (m, 13H), 1.24–1.20 (m, 1H). ^**13**^ **C­{** ^**1**^ **H} NMR** (100 MHz, CD_3_OD): δ 174.8, 174.5, 170.6, 158.9, 158.6, 158.1, 158.0, 138.4, 138.2, 138.14, 138.05, 135.8, 135.7, 129.6, 129.48, 129.45, 129.32, 129.30, 129.04, 128.93, 128.90, 128.8, 82.7, 68.34, 68.27, 67.9, 67.3, 55.7, 55.1, 43.4, 41.5, 35.0, 34.6, 32.5, 32.1, 31.1, 30.4, 28.3, 28.0, 27.5, 24.0, 22.0, 15.3. Additional signals in ^13^C NMR spectrum are observed due to the presence of methyl carbamate (CbzMeN-) rotamers. Chemical shifts for both rotamers are described. **FTIR** (thin film) cm^–1^: 3296, 2935, 2461, 1679, 1626, 1531, 1428, 1365. **HRMS (ESI)** (*m*/*z*): Calc.’d for C_47_H_63_N_6_O_11_S [M + H]^+^: 919.4270, found 919.4180.
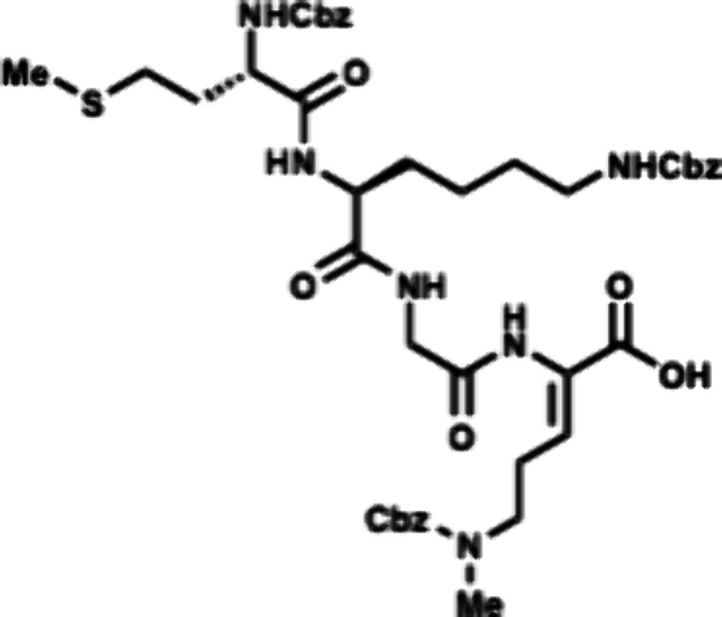


**Acid 26**. A flame-dried round-bottom flask was charged with tetrapeptide **25** (4.6 g, 5 mmol, 1.0 equiv). The reaction vessel was evacuated and backfilled with nitrogen gas and this process was repeated three times. Anhydrous CH_2_Cl_2_ (50 mL) was introduced via cannula. Then, zinc bromide (11.2 g, 50 mmol, 10.0 equiv) was added to the reaction mixture and the reaction was left stirring for 16 h. After 16 h, the reaction was quenched with 1 M HCl (50 mL) and the reaction mixture was then extracted with CH_2_Cl_2_ (6 × 80 mL). The combined organic layer was washed with brine and dried over with anhydrous sodium sulfate, filtered, and concentrated under reduced pressure. The resulting crude mixture was purified by silica gel column chromatography (1–4% MeOH in CH_2_Cl_2_) to provide the acid **26** (3.0 g, 70% yield) as a white solid. **M.p.**: 195–196 °C.^1^ **H NMR** (400 MHz, CD_3_OD): δ 7.40–7.21 (m, 15H), 6.71 (t, *J* = 7.4 Hz, 1H), 5.15–5.01 (m, 6H), 4.35–4.20 (m, 2H), 4.07–3.96 (m, 1H), 3.96–3.86 (m, 1H), 3.41 (t, *J* = 7.1 Hz, 2H), 3.09 (t, *J* = 6.7 Hz, 2H), 2.90 (s, 3H), 2.63–2.45 (m, 2H), 2.45–2.35 (m, 2H), 2.04 (s, 3H), 1.95–1.80 (m, 2H), 1.77–1.63 (m, 1H), 1.56–1.26 (m, 5H). ^**13**^ **C­{** ^**1**^ **H} NMR** (100 MHz, CD_3_OD): δ 174.8, 174.6, 170.5, 167.8, 158.9, 158.6, 158.0, 138.4, 138.2, 138.0, 136.8, 129.5, 129.5, 129.4, 129.0, 128.91, 128.86, 128.8, 68.4, 68.3, 67.9, 67.3, 55.6, 55.1, 43.6, 41.5, 35.0, 34.6, 32.5, 32.2, 31.1, 30.4, 28.3, 27.8, 24.0, 15.3. Additional signals in ^13^C NMR spectrum are observed due to the presence of methyl carbamate (CbzMeN-) rotamers. Chemical shifts for both rotamers are described. Aromatic carbon signals from the Cbz groups are overlapping. **FTIR** (thin film) cm^–1^: 3680, 3305, 2937, 2843, 1652, 1522, 1404, 1360. **HRMS (ESI)** (*m*/*z*): Calc.’d for C_43_H_55_N_6_O_11_S [M + H]^+^: 863.3644, found 863.3617.
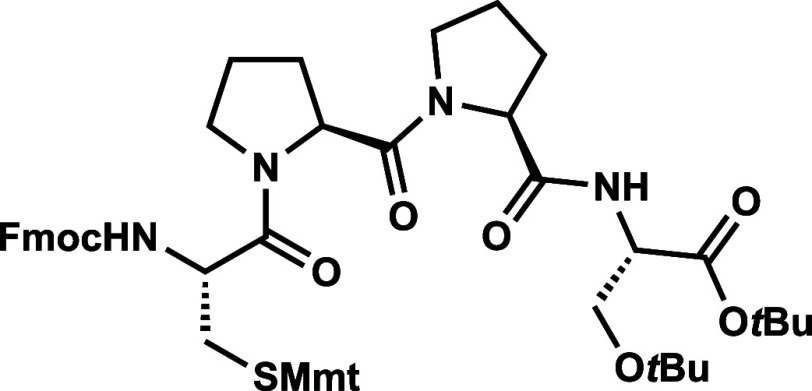


**Tetrapeptide 32**. A flame-dried round-bottom flask was charged with tripeptide **29** (4.4 g, 7.1 mmol, 1.0 equiv) and equipped with a stir bar. The reaction vessel was evacuated and backfilled with nitrogen gas and this process repeated three times. Dry CH_2_Cl_2_ (6 mL) was added to the reaction and the reaction vessel cooled to 0 °C. At this time, DBU (1.2 mL, 795 mmol, 1.1 equiv) was added dropwise and the reaction was left stirring at 90 min at 0 °C. After the starting material was consumed by TLC, cysteine **31** (4.8 g, 7.9 mmol, 1.1 equiv), HOAt (2.9 g, 21.3 mmol, 3.0 equiv), and EDC (3.7 mL, 21.3 mmol, 3.0 equiv) were added to the reaction mixture. The reaction mixture was allowed to warm slowly to room temperature and stirred for 16 h. The reaction mixture was then quenched with deionized water (200 mL) and extracted with EtOAc (3 × 400 mL). The combined organic layer was washed with brine, and then dried over anhydrous Na_2_SO_4_, filtered, and concentrated *in vacuo*. The resulting crude mixture was purified by silica gel column chromatography (60 to 80% EtOAc in Hexanes) to provide the tetrapeptide **32** (5.7 g, 79% yield) as a white solid. **M.p.**: 104–105 °C.^1^ **H NMR** (400 MHz, CDCl_3_) δ 7.73 (t, *J* = 6.6 Hz, 2H), 7.58 (d, *J* = 7.5 Hz, 2H), 7.43–7.33 (m, 8H), 7.33–7.22 (m, 10H), 7.18 (t, *J* = 7.2 Hz, 2H), 6.80 (t, *J* = 7.8 Hz, 2H), 5.23 (d, *J* = 8.9 Hz, 1H), 4.56 (dd, *J* = 5.5, 2.7 Hz, 2H), 4.48 (dt, *J* = 7.9, 2.9 Hz, 1H), 4.36 (dd, *J* = 10.4, 7.3 Hz, 1H), 4.32–4.24 (m, 2H), 4.19 (t, *J* = 7.2 Hz, 1H), 3.75 (s, 3H), 3.76–3.70 (m, 2H), 3.58–3.47 (m, 2H), 3.35 (q, *J* = 7.1 Hz, 1H), 3.04 (q, *J* = 6.5 Hz, 1H), 2.70–2.55 (m, 2H), 2.26–2.15 (m, 1H), 2.15–1.89 (m, 8H), 1.83 (dt, *J* = 12.3, 6.4 Hz, 2H), 1.44 (s, 9H), 1.11 (s, 9H).^13^ **C­{** ^**1**^ **H} NMR** (100 MHz, CDCl_3_): δ 171.3, 170.8, 169.4, 168.9, 158.2, 156.1, 144.9, 144.8, 143.9, 143.8, 141.3, 136.5, 131.1, 129.8, 129.7, 128.1, 127.7, 127.1, 127.1, 126.8, 125.3, 125.3, 120.0, 113.3, 81.7, 73.0, 67.10, 67.06, 61.9, 59.9, 58.0, 55.3, 53.3, 52.3, 47.1, 47.0, 33.8, 28.5, 28.4, 28.1, 27.4, 25.0, 24.8. **FTIR** ν_max_ (neat)/ cm^–1^ = 3282, 2935, 1684, 1633, 1526, 1440, 1249, 1025, 821, 739. **HRMS (ESI)** calculated for C_59_H_69_N_4_O_9_S ([M + H]^+^): 1009.4780; found 1009.4722.
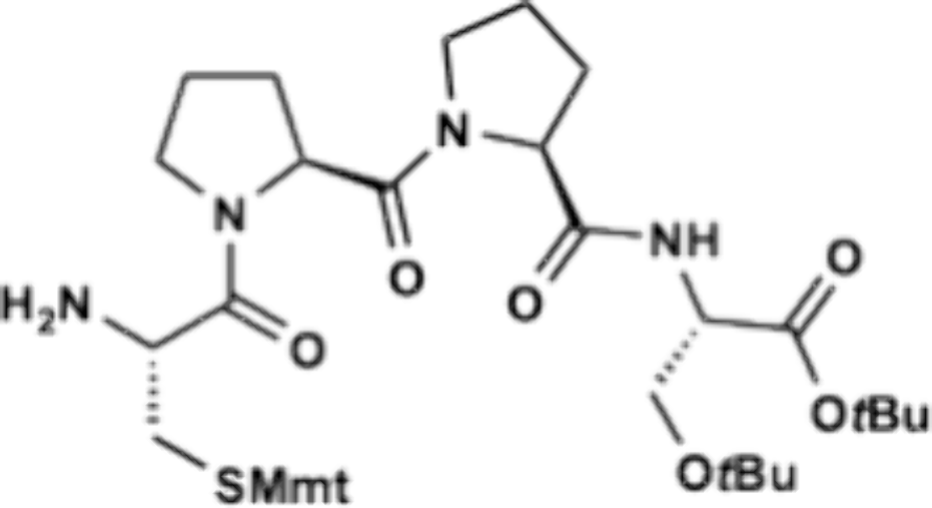


**Tetrapeptide 33.** A flame-dried round-bottom flask was charged with tetrapeptide **32** (60 mg, 0.06 mmol, 1.0 equiv) and equipped with a stir bar. The reaction vessel was evacuated and backfilled with nitrogen gas and this process repeated three times. Anhydrous CH_2_Cl_2_ (4 mL) was added to the reaction and the reaction vessel cooled to 0 °C. At this time, DBU (8.8 mL, 0.06 mmol, 1.0 equiv) was added and the reaction mixture was allowed to warm slowly to room temperature and stirred for 16 h. Afterward, the reaction mixture was concentrated *in vacuo*. The resulting crude mixture was directly purified by silica gel column chromatography (2 to 5% MeOH in CH_2_Cl_2_) to provide amine **33** (47 mg, quantitative yield) as a white foam.^1^ **H NMR** (400 MHz, CDCl_3_): δ 7.46–7.36 (m, 4H), 7.35–7.29 (m, 2H), 7.29–7.22 (m, 4H), 7.22–7.14 (m, 2H), 6.83–6.75 (m, 3H), 4.61–4.50 (m, 2H), 4.50–4.42 (m, 1H), 3.77 (s, 3H), 3.77–3.69 (m, 1H), 3.66–3.62 (m, 1H), 3.58–3.54 (m, 2H), 3.52 (dd, *J* = 8.6, 3.0 Hz, 1H), 3.24–3.15 (m, 1H), 3.14–3.05 (m, 1H), 2.98–2.89 (m, 1H), 2.66–2.55 (m, 1H), 2.44–2.14 (m, 3H), 2.12–1.90 (m, 4H), 1.86–1.74 (m, 1H), 1.43 (s, 9H), 1.12 (s, 9H). ^**13**^ **C­{** ^**1**^ **H} NMR** (100 MHz, CDCl_3_): δ 171.3, 171.1, 169.4, 158.3, 145.2, 145.1, 131.1, 129.8, 129.7, 128.1, 126.8, 113.4, 81.7, 73.0, 70.7, 66.9, 61.9, 59.9, 58.0, 55.3, 53.4, 52.9, 47.2, 46.8, 28.6, 28.4, 28.1, 27.5, 25.0, 24.8. **FTIR** (thin film) cm^–1^: 3372, 2974, 2828, 1737, 1637, 1508, 1444, 1393. **HRMS (ESI)** (*m*/*z*): Calc.’d for C_44_H_59_N_4_O_7_S [M + H]^+^: 787.4099, found 787.4068.
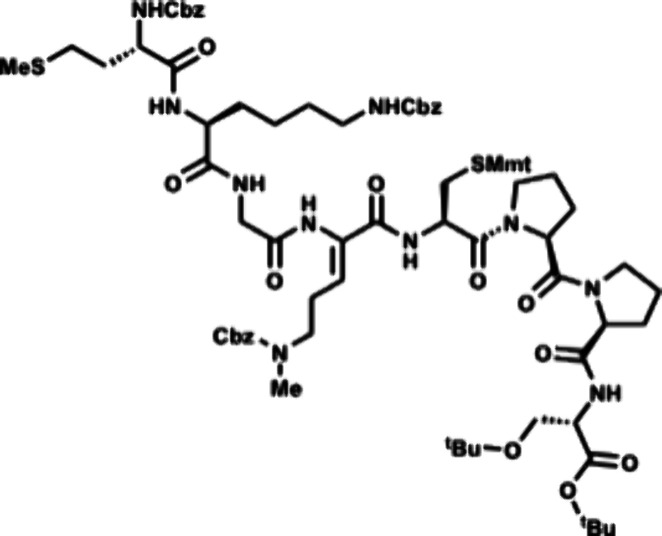


**Octapeptide 34.** A flame-dried 50 mL round-bottom flask was charged with carboxylic acid **26** (800.0 mg, 0.93 mmol, 1.0 equiv) and *N*-hydroxysuccinimide (160.0 mg, 1.39 mmol, 1.5 equiv). The reaction vessel was evacuated and backfilled with nitrogen gas, and this process was repeated three times. Anhydrous CH_2_Cl_2_ (8 mL) was added, and the resulting mixture was cooled to 0 °C. Then, *N*,*N’* dicyclohexylcarbodiimide (286.9 mg, 1.39 mmol, 1.5 equiv) was added as a solid. The reaction mixture was stirred under an ice bath for 1 h. Next, amine **33** (729.6 mg, 0.93 mmol, 1.0 equiv) in CH_2_Cl_2_ (7 mL) was added and the reaction mixture was allowed to warm slowly to room temperature and stirred for overnight. On complete consumption of all the starting material as indicated by TLC, the reaction mixture was quenched by adding brine and extracted with CH_2_Cl_2_ (3 × 25 mL). The combined organic layer was washed brine, and then dried over anhydrous Na_2_SO_4_, filtered, and concentrated in *vacuo*. The crude mixture was purified by silica gel column chromatography (0% to 5% MeOH in CH_2_Cl_2_) to afford octapeptide **34** (760.0 mg, 50% yield) as a white solid. **M.p.**: 105–106 °C.^1^ **H NMR** (400 MHz, CD_3_OD): δ 7.42–7.23 (m, 25H), 7.18 (d, *J* = 1.6 Hz, 2H), 6.88–6.78 (m, 2H), 6.61–6.43 (m, 1H), 5.18–4.98 (m, 6H), 4.55–4.45 (m, 2H), 4.45–4.35 (m, 2H), 4.32–4.19 (m, 2H), 4.02–3.82 (m, 2H), 3.79 (dd, *J* = 9.1, 3.8 Hz, 1H), 3.75 (s, 3H), 3.67 (dd, *J* = 7.9, 7.3 Hz, 1H), 3.55 (dd, *J* = 9.0, 3.6 Hz, 1H), 3.53–3.49 (m, 1H), 3.44–3.35 (m, 3H), 3.08 (t, *J* = 7.2 Hz, 2H), 2.94–2.87 (m, 4H), 2.87–2.82 (m, 1H), 2.65 (dd, *J* = 13.4, 4.9 Hz, 1H), 2.59–2.43 (m, 2H), 2.42–2.30 (m, 2H), 2.21–2.04 (m, 4H), 2.02 (s, 3H), 1.99–1.76 (m, 8H), 1.76–1.64 (m, 1H), 1.55–1.42 (m, 10H), 1.42–1.27 (m, 2H), 1.17 (s, 9H).^13^ **C­{** ^1^ **H} NMR** (100 MHz, CD_3_OD): δ 175.1, 174.9, 174.1, 172.4, 170.9, 170.4, 159.9, 146.6, 146.4, 138.5, 138.2, 138.1, 137.8, 132.3, 132.1, 130.9, 130.8, 130.7, 130.6, 129.6, 129.53, 129.47, 129.08, 129.04, 129.01, 128.95, 128.90, 128.8, 127.8, 114.3, 82.9, 74.3, 68.4, 68.0, 67.9, 67.4, 62.9, 61.2, 59.7, 55.8, 55.1, 52.9, 44.1, 41.5, 34.1, 32.5, 31.9, 31.2, 30.5, 30.2, 29.4, 28.3, 27.7, 25.8, 25.6, 24.0, 15.4. Additional signals in ^13^C NMR spectrum are observed due to the presence of methyl carbamate (CbzMeN-) rotamers. Chemical shifts for both rotamers are described. **FTIR** (thin film) cm^–1^: 3293, 2970, 1636, 1507, 1441, 1364, 1300, 1246. **HRMS (ESI)** (*m*/*z*): Calc.’d for C_87_H_111_N_10_O_17_S_2_ [M + H]^+^: 1631.7565, found 1631.7464.
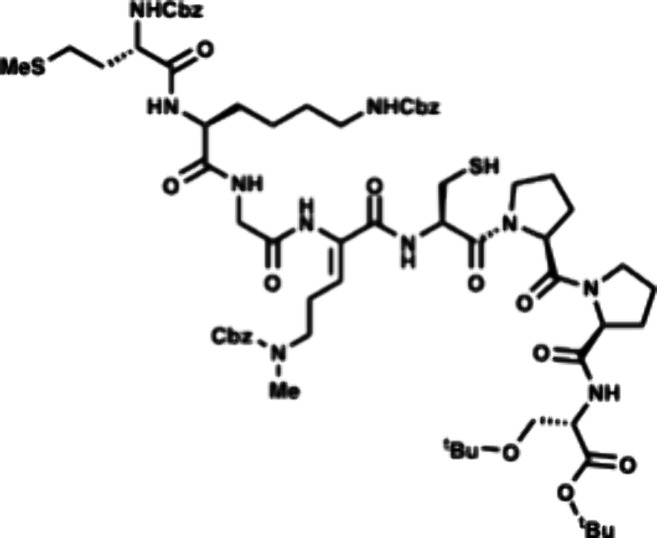


**Thiol 35**. A flame-dried round-bottom flask was charged with octapeptide **34** (100 mg, 0.06 mmol, 1.0 equiv). The reaction vessel was evacuated and backfilled with nitrogen gas and this process was repeated three times. Anhydrous CH_2_Cl_2_ (10 mL) was added via syringe and followed by the sequential addition of TFA (0.1 mL, 1% v/v) and triisopropylsilane (0.1 mL, 1% v/v). After 1 h, the reaction mixture was concentrated under reduced pressure. The crude mixture was then purified by reversed-phase C18 column chromatography using a Combi-Flash Rf automated chromatography instrument equipped with a 50 g RediSep Gold C18Aq Column (0% to 85% MeCN in H_2_O) to afford thiol **35** (64 mg, 78%) as a white solid. **M.p.**: 154–155 °C.^1^ **H NMR** (400 MHz, CD_3_OD): δ 7.42–7.22 (m, 15H), 6.64–6.45 (m, 1H), 5.17–5.00 (m, 6H), 4.87–4.82 (m, 1H), 4.66 (dd, *J* = 8.8, 4.4 Hz, 1H), 4.58–4.49 (m, 1H), 4.41 (dd, *J* = 3.3, 3.2 Hz, 1H), 4.34–4.21 (m, 2H), 4.00–3.83 (m, 3H), 3.79 (dd, *J* = 8.9, 3.6 Hz, 2H), 3.75–3.67 (m, 1H), 3.64–3.58 (m, 2H), 3.55 (dd, *J* = 9.1, 3.4 Hz, 2H), 3.46–3.34 (m, 2H), 3.10 (t, *J* = 6.8 Hz, 2H), 2.97–2.92 (m, 1H), 2.90 (s, 3H), 2.77 (dd, *J* = 13.8, 6.7 Hz, 1H), 2.60–2.45 (m, 2H), 2.45–2.32 (m, 2H), 2.29–2.07 (m, 3H), 2.06 (s, 3H), 2.00–1.79 (m, 8H), 1.79–1.65 (m, 1H), 1.55–1.48 (m, 1H), 1.48 (s, 9H), 1.43–1.32 (m, 1H), 1.17 (s, 9H).^13^ **C­{** ^1^ **H} NMR** (100 MHz, CD_3_OD): δ 174.9, 174.0, 172.5, 171.1, 170.8, 170.5, 166.2, 158.8, 158.6, 158.1, 157.9, 138.4, 138.2, 138.1, 134.4, 133.8, 131.3, 130.9, 129.6, 129.56, 129.53, 129.51, 129.1, 129.0, 128.93, 128.87, 128.86, 128.77, 128.76, 82.8, 74.3, 68.4, 68.3, 67.8, 67.3, 62.9, 61.2, 59.9, 55.6, 55.5, 55.2, 55.0, 44.1, 41.5, 35.2, 34.8, 32.5, 32.0, 31.2, 30.4, 30.2, 29.5, 28.4, 28.3, 27.72, 27.69, 26.57, 25.83, 25.79, 23.96, 18.22, 18.19, 15.40, 15.37, 13.6. Additional signals in ^13^C NMR spectrum are observed due to the presence of methyl carbamate (CbzMeN-) rotamers. Chemical shifts for both rotamers are described. **FTIR** (thin film) cm^–1^: 3674, 2972, 2865, 2463, 1635, 1419, 1362, 1246. **HRMS** (ESI) (*m*/*z*): Calc.’d for C_67_H_95_N_10_O_16_S_2_ [M + H]^+^: 1359.6363, found 1359.6228.

**Procedure for Thiol Cyclization of 35.** To a flame-dried, 10 mL reaction tube equipped with a magnetic stir bar was charged with thiol **35** (10.0 mg, 7.4 μmol, 1.0 equiv). The reaction vessel was evacuated and backfilled with nitrogen gas three times. Anhydrous solvent (1 mL) was added to the reaction tube followed by the addition of dtPa **36** (0.74 μmol, 0.1 equiv or 3.7 μmol, 0.5 equiv). The reaction was stirred at room temperature for 12 h or heated to 90 °C in an oil bath for 12 h. After this time, reaction mixture was cooled to room temperature, concentrated in *vacuo*, and the reaction was analyzed by NMR.

**Procedure for Thiol Cyclization of 35 with microwave irradation.** To a 20 mL glass microwave vessel equipped with a magnetic stir bar was charged with thiol **35** (10.0 mg, 7.4 μmol, 1.0 equiv). The reaction vessel was evacuated and backfilled with nitrogen gas three times. Anhydrous α,α,α-trifluorotoluene (2 mL) was added to the reaction vessel followed by the addition of dtPa **36** (0.74 μmol, 0.1 equiv). The reaction was heated to 150 °C in a CEM Mars 6 Microwave Synthesizer for 2 h. After this time, reaction mixture was cooled to room temperature and concentrated in *vacuo*. The reaction was analyzed by NMR.
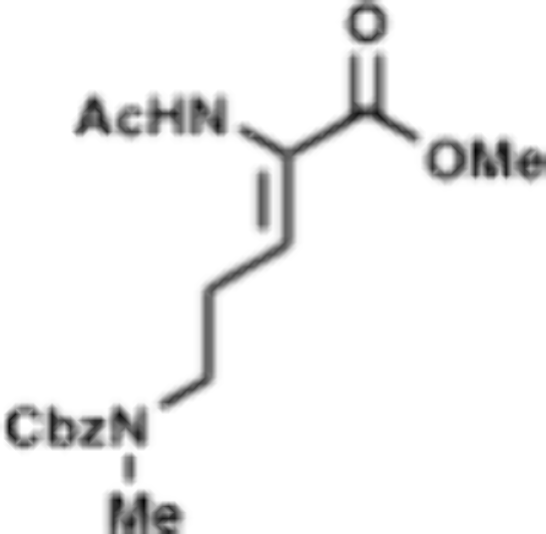


**Alkene 46**. A flame-dried round-bottom flask was charged with phosphonate **47** (1.2 g, 5.0 mmol, 1.0 equiv). The reaction vessel was evacuated and backfilled with nitrogen gas and this process was repeated three times. Anhydrous CH_2_Cl_2_ (50 mL) was introduced via cannula. Then, tetramethylguanidine (1.6 mL, 12.5 mmol, 2.5 equiv) was added to the reaction mixture followed by the addition of aldehyde **24** (1.3 g, 10.0 mmol, 2.0 equiv) in 50 mL CH_2_Cl_2_. After 15 min, reaction mixture was quenched with 1 M HCl (20 mL) and the reaction mixture was then extracted with CH_2_Cl_2_ (3 × 50 mL). The combined organic layer was washed with brine and dried over with anhydrous Na_2_SO_4_, filtered, and concentrated under reduced pressure. The resulting crude mixture was purified by silica gel column chromatography (60% EtOAc in Hexanes) to provide alkene **46** (1.5 g, 92% yield) as a colorless oil. NMR signals are split due to the presence of a 1:1 ratio of amide rotamers. Chemical shifts for both rotamers are described. ^**1**^ **H NMR** (400 MHz, CDCl_3_): δ 7.40–7.28 (m, 5H), 7.19 (bs, 0.5H, NH), 6.85 (bs, NH, 0.5H), 6.68–6.54 (m, 1H), 5.13 (s, 2H), 3.76 (s, 3H), 3.50–3.37 (m, 2H), 2.93 (s, 3H), 2.51–2.35 (m, 2H), 2.10 (s, 3H). ^**13**^ **C­{** ^**1**^ **H} NMR** (100 MHz, CDCl_3_): δ 168.7, 168.4, 165.0, 156.5, 156.3, 136.9, 134.2, 133.0, 128.6, 128.1, 127.9, 127.0, 126.1, 67.2, 52.6, 48.0, 47.2, 34.8, 34.5, 28.3, 27.5, 23.5. **FTIR** (thin film) cm^–1^: 3270, 2950, 1667, 1497, 1434, 1402, 1366, 1254. **HRMS (ESI)** (*m*/*z*): Calc.’d for C_17_H_23_N_2_O_5_ [M + H]^+^: 335.1601, found 335.1589.
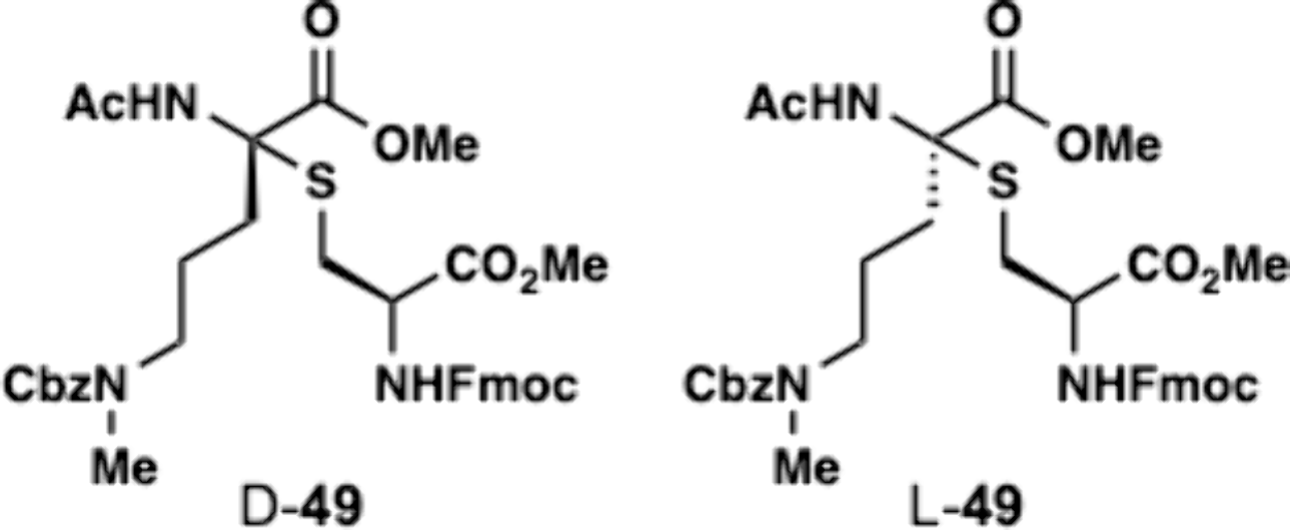


**Sactionine** D-**49** and L-**49**. An oven-dried 10 mL tube was charged with alkene **46** (400 mg, 1.2 mmol, 1.0 equiv) and cysteine **48** (850 mg, 2.4 mmol, 2.0 equiv) and dithiophosphoric acid **36** (34 mg, 0.12 mmol, 0.1 equiv). Dithiophosphoric acid **36** was prepared in one step according to Hu, B.^50^ The reaction vessel was evacuated and backfilled with nitrogen gas and this process was repeated three times. Anhydrous fluorobenzene (5 mL) was introduced via syringe. The reaction mixture was then heated to 90 °C in an oil bath and heated to reflux for 12 h. After this time, the reaction was cooled to room temperature and concentrated under reduced pressure. The crude mixture was purified by silica gel column chromatography (EtOAc/Ether/Hexanes = 8:1:1) to provide sactionine D-**49** (275 mg, 33%) and L-**49** (229 mg, 28%) as white foams. NMR signals are split due to the presence of a 1:1 ratio of amide rotamers. Chemical shifts for both rotamers are described. D-**49**: ^**1**^ **H NMR** (400 MHz, CDCl_3_): δ 7.77 (d, *J* = 7.5 Hz, 2H), 7.61 (d, *J* = 7.5 Hz, 2H), 7.54 (bs, 1H), 7.40 (t, *J* = 7.4 Hz, 2H), 7.38–7.27 (m, 7H), 5.83–5.69 (bs, 1H), 5.09 (s, 2H), 4.63 (dd, *J* = 7.1, 7.1 Hz, 1H), 4.41 (d, *J* = 7.3 Hz, 2H), 4.24 (t, *J* = 7.1 Hz, 1H), 3.85–3.73 (m, 6H), 3.31–3.19 (m, 2H), 3.19–3.12 (m, 1H), 2.84 (s, 3H), 2.81–2.70 (m, 1H), 2.38–2.24 (m, 2H), 2.01 (s, 1.5H, N-COCH_3_), 1.97 (s, 1.5H, N-COCH_3_), 1.76–1.65 (m, 1H), 1.65–1.51 (m, 1H). ^**13**^ **C­{** ^**1**^ **H} NMR** (100 MHz, CDCl_3_): δ 173.4, 171.4, 169.9, 156.3, 137.1, 128.6, 128.6, 127.9, 127.9, 67.1, 64.0, 63.8, 53.0, 52.8, 52.5, 49.0, 48.6, 34.7, 34.2, 33.6, 31.2, 23.0, 22.6. **FTIR** (thin film) cm^–1^: 3280, 2950, 1733, 1677, 1515, 1435, 1404, 1370. **HRMS** (ESI) (*m*/*z*): Calc.’d for C_36_H_42_N_3_O_9_S [M + H]^+^: 692.2636, found 692.2691. L-**49**: ^**1**^ **H NMR** (400 MHz, CDCl_3_): δ 7.76 (d, *J* = 7.5 Hz, 2H), 7.60 (t, *J* = 7.4 Hz, 2H), 7.45–7.38 (m, 2H), 7.38–7.28 (m, 7H), 6.82 (bs, N–H, 0.5H), 6.70 (bs, N–H, 0.5H), 5.56 (bs, 1H), 5.11 (s, 2H), 4.62–4.51 (m, 1H), 4.46 (dd, *J* = 8.7, 8.7 Hz, 1H), 4.37 (dd, *J* = 8.8, 8.8 Hz, 1H), 4.23 (t, *J* = 7.0 Hz, 1H), 3.85–3.62 (m, 6H), 3.38–3.11 (m, 2H), 3.08–2.95 (m, 1H), 2.88 (s, 3H), 2.66–2.52 (m, 1H), 2.18–2.06 (m, 1H), 2.03 (s, 1.5H, N-COCH_3_), 1.99 (s, 1.5H, N-COCH_3_), 1.65–1.48 (m, 1H), 1.43–1.29 (m, 1H). ^**13**^ **C­{** ^**1**^ **H} NMR** (100 MHz, CDCl_3_): δ 171.2, 170.9, 169.1, 156.3, 155.8, 143.9, 143.7, 141.4, 137.0, 128.6, 128.0, 127.9, 127.2, 125.2, 125.1, 120.1, 67.4, 67.1, 66.8, 66.6, 54.0, 53.6, 53.1, 48.7, 48.3, 47.2, 34.8, 34.3, 31.9, 31.7, 31.4, 30.4, 29.8, 23.9, 23.4, 22.9.. NMR signals are split due to the presence of a 1:1 ratio of amide rotamers. Chemical shifts for both rotamers are described. **FTIR** (thin film) cm^–1^: 3396, 2953, 1724, 1688, 1498, 1437, 1405, 1367. **HRMS** (ESI) (*m*/*z*): Calc.’d for C_36_H_42_N_3_O_9_S [M + H]^+^: 692.2636, found 692.2687.
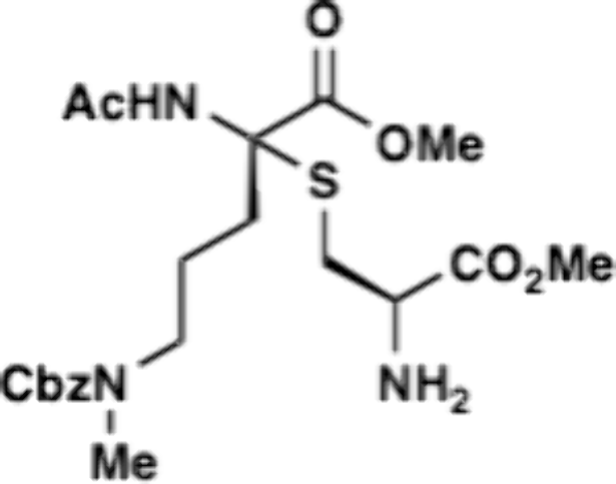


**Amine D-50.** A flame-dried round-bottom flask equipped with a magnetic stir bar was charged with D-**49** (570 mg, 0.82 mmol, 1 equiv). The reaction vessel was evacuated and backfilled with nitrogen gas and this process was repeated three times. The reaction was added anhydrous CH_2_Cl_2_ (57 mL), followed by the dropwise addition of piperidine (2.85 mL, 28.85 mmol, 5% v/v) at room temperature. The reaction mixture was stirred at room temperature for 2 h. At this time, the reaction was quenched with 50 mL of deionized water, and the reaction mixture was extracted with CH_2_Cl_2_ (5 × 20 mL). The combined organic layer was washed with brine and dried over with anhydrous sodium sulfate, filtered and concentrated under reduced pressure. The resulting crude mixture was purified by silica gel column chromatography (0.5–2.5% methanol in CH_2_Cl_2_) to afford amine D-**50** (235 mg, 61% yield) as a pale-yellow oil. NMR signals are split due to the presence of a 1:1 ratio of amide rotamers. Chemical shifts for both rotamers are described.^1^ **H NMR** (400 MHz, CDCl_3_) δ 9.10 (bs, N–H, 0.5H), 9.04 (bs, N–H, 0.5H), 7.39–7.27 (m, 5H), 5.11 (s, 2H), 3.89 (t, *J* = 4.2 Hz, 1H), 3.78–3.72 (m, 6H), 3.35–3.22 (m, 2H), 3.18 (dd, *J* = 15.0, 4.5 Hz, 1H), 3.07 (dd, *J* = 15.0, 3.8 Hz, 1H), 2.93–2.86 (s, 3H), 2.48 (ddd, *J* = 14.6, 11.9, 4.9 Hz, 1H), 2.21 (bs, 2H), 2.04–1.98 (d, *J* = 15.6 Hz, 4H), 1.75–1.62 (m, 1H), 1.57–1.43 (m, 1H).^13^ **C NMR­{** ^**1**^ **H}** (100 MHz, CDCl_3_) δ 173.4, 171.4, 169.9, 156.3, 137.1, 128.62, 128.58, 127.94, 127.86, 67.1, 64.0, 63.8, 53.0, 52.8, 52.5, 49.0, 48.6, 34.7, 34.2, 33.6, 31.0 23.0, 22.6. **FTIR** (thin film) cm^–1^: 3304, 2951, 1735, 1690, 1485, 1435, 1233, 1199, 1011, 911, 727. **[α]**_**24**_^**D**^ = −17.42 (*c* = 1.0, MeOH). **HRMS (ESI)** Calc.’d for C_21_H_31_N_3_O_7_S ([M + H]^+^): 470.1955; found 470.1861.

**Procedure for Amidative Cyclization with Lewis acids and bases.** To a flame-dried, 10 mL reaction tube equipped with a magnetic stir bar was charged with amine D-**50** (5.0 mg, 10.6 μmol, 1.0 equiv). The reaction vessel was evacuated and backfilled with nitrogen gas three times. Anhydrous solvent (1 mL) was added to the reaction tube followed by the addition of either acid (TFA, BF_3_OEt_2_, AlCl_3_, MgBr_2_) or base (DBU, KO^*t*^Bu, K_2_CO_3_) (10.6 μmol, 1.0 equiv). The reaction was stirred at room temperature or heated to reflux in an oil bath for 12 h. On complete consumption of all the starting material as indicated by TLC, the reaction mixture was quenched by adding water, extracted with EtOAc (3 × 5 mL). The combined organic layer was washed with brine, and then dried over anhydrous Na_2_SO_4_, filtered, and concentrated in *vacuo.* 
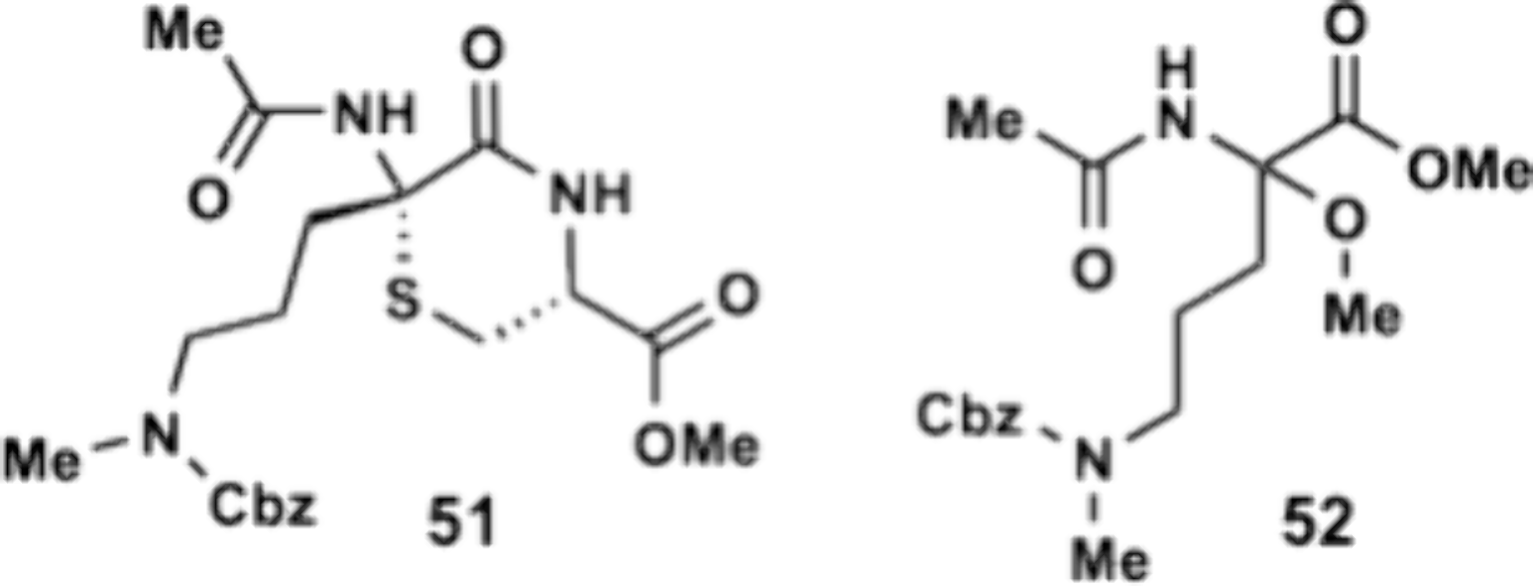


**Thiomorpholine 51** and **Aminoketal 52.** To a flame-dried, 50 mL round-bottom flask charged with amine D-**50** (80.0 mg, 0.17 mmol, 1.0 equiv), K_2_CO_3_ (23.5 g, 0.17 mmol, 1.0 equiv), and a magnetic stir bar was added MeOH (8 mL). The reaction was stirred at room temperature for 12 h. On complete consumption of all the starting material as indicated by TLC, the reaction mixture was quenched by adding water and concentration *in vacuo* to remove methanol. The crude residue was extracted with CH_2_Cl_2_ (3 × 25 mL). The combined organic layer was washed brine, and then dried over anhydrous Na_2_SO_4_, filtered, and concentrated in *vacuo*. The crude mixture was purified by silica gel column chromatography (0% to 3% MeOH in CH_2_Cl_2_) to afford thiomorpholine **51** (7.5 mg, 10% yield) and aminoketal **52** (28.2 mg, 45% yield) as clear oils. Thiomorpholine **51**: ^**1**^ **H NMR** (400 MHz, CD_3_OD): δ 7.33–7.24 (m, 5H), 5.07 (s, 2H), 4.43–4.29 (m, 1H), 3.77 (s, 3H), 3.52 (t, *J* = 11.6 Hz, 1H), 3.28–3.27 (m, 2H), 3.08–2.99 (m, 1H), 2.89 (s, 3H), 2.02–1.92 (m, 1H), 1.88 (s, 3H), 1.84–1.73 (m, 2H), 1.69–1.58 (m, 1H). ^**13**^ **C­{** ^**1**^ **H} NMR** (100 MHz, CD_3_OD): δ 172.6, 170.8, 170.4, 158.0, 138.2, 129.6, 129.1, 128.8, 68.3, 63.6, 59.3, 53.5, 49.3, 38.7, 35.0, 34.3, 29.5, 23.8, 23.4, 22.4. **FTIR**: ν_max_ (neat)/ cm^–1^ = 3300, 2952, 1743, 1658, 1484, 1402, 1309, 1210, 1150, 1026, 976. **HRMS (ESI)**: Calc.’d for C_20_H_28_N_3_O_6_S ([M + H]^+^): 438.1693; found 438.1721. Aminoketal **52**: ^**1**^ **H NMR** (400 MHz, CDCl_3_, VT 50 °C): δ 7.36–7.28 (m, 5H), 5.12 (s, 2H), 3.77 (s, 3H), 3.31–3.24 (m, 5H), 2.89 (s, 3H), 2.37–2.27 (m, 1H), 2.03 (s, 3H), 1.94–1.85 (m, 1H), 1.54–1.44 (m, 2H). ^**13**^ **C­{** ^**1**^ **H} NMR** (100 MHz, CDCl_3_): δ 170.5, 169.8, 169.6, 156.6, 156.2, 136.9, 128.6, 128.1, 127.9, 87.4, 86.8, 67.1, 53.1, 51.7, 48.5, 48.3, 34.7, 34.0, 33.0, 32.8, 23.7, 23.5, 22.2, 21.6. **FTIR**: ν_max_ (neat)/ cm^–1^ = 3313, 2947, 1750, 1673, 1532, 1454, 1196, 1151, 1094, 1059, 917. **HRMS (ESI)**: Calc.’d for C_18_H_26_N_2_NaO_6_ ([M + Na]^+^): 389.1683; found 389.1665.

## Supplementary Material





## Data Availability

The data underlying this study are available in the published article and its Supporting Information.

## Notes

FAIR data, including the primary NMR FID files, for compounds **16**, **17**, **20**, **21**, **23**, **25**, **26**, **32**–**35**, **46**, D-**49**, L-**49**, D-**50**, **51**, and **52** (ZIP)
NMR spectra for all new compounds (PDF)
