# Supplementary material for: Evolution of a Strategy for the Unified Synthesis of Enteropeptin Sactipeptides
Source: J Org Chem. 2026 Feb 23;91(9):3529–44. doi: 10.1021/acs.joc.5c03063 (PMC12973295; doi:10.1021/acs.joc.5c03063)
Supplement: Supplementary file 1 [file jo5c03063_si_001.zip › Compound 21 - 1H/1/pdata/1/email_yz-MKG-OH-1H-dilute-DMSO_1_1.pdf]

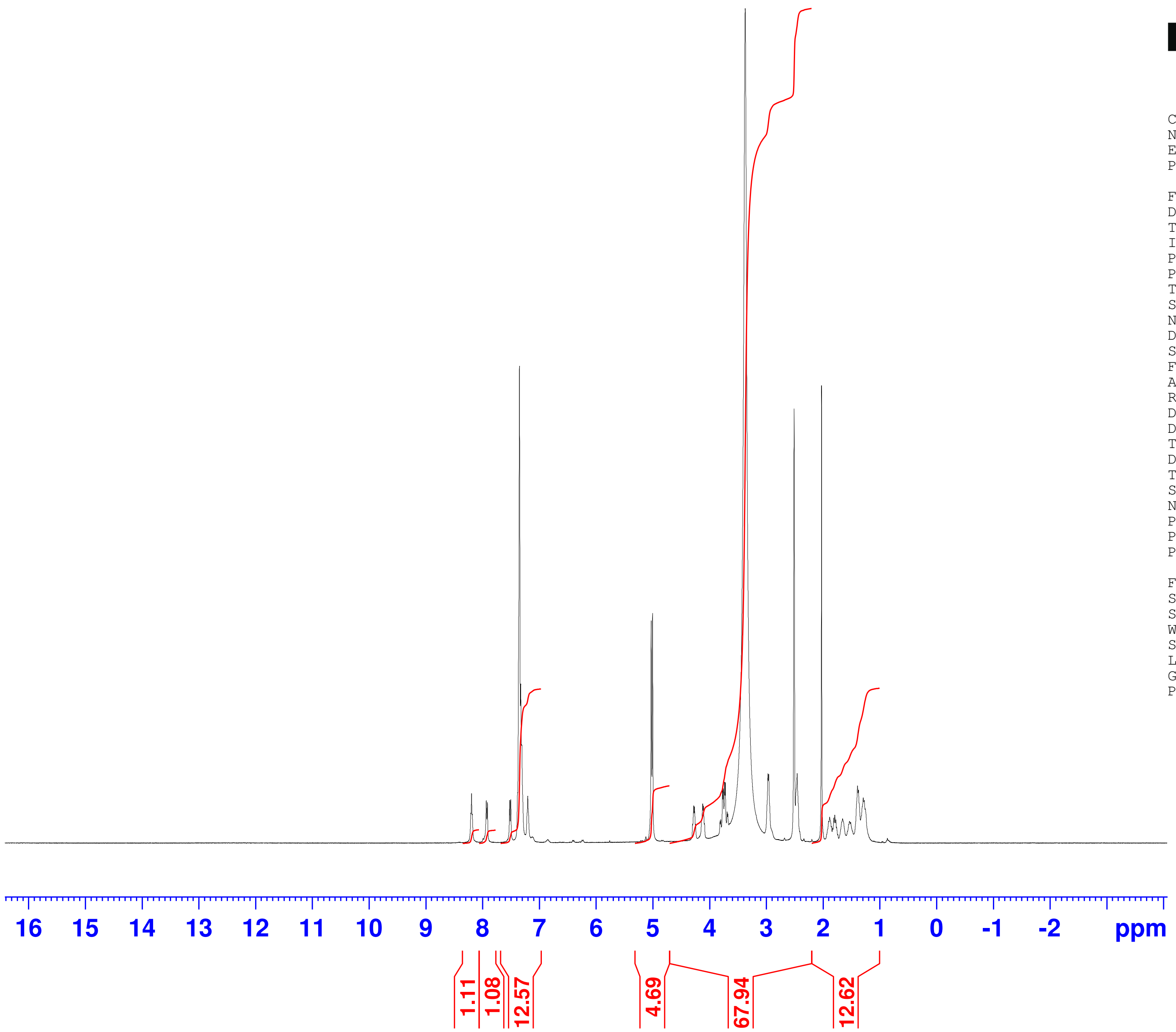

BRUKER

Current Data Parameters

NAME	yz-MKG-OH-1H-dilute-DMSO
EXPNO	1
PROCNO	1

F2 - Acquisition Parameters

Date_	20251121
Time	16.52 h
INSTRUM	Avance
PROBHD	Z163739_0940 (
PULPROG	zg30
TD	65536
SOLVENT	DMSO
NS	16
DS	2
SWH	8196.721 Hz
FIDRES	0.250144 Hz
AQ	3.9976959 sec
RG	101
DW	61.000 usec
DE	13.89 usec
TE	298.0 K
D1	1.00000000 sec
TD0	1
SFO1	400.1324708 MHz
NUC1	1H
P0	2.67 usec
P1	8.00 usec
PLW1	23.00000000 W

F2 - Processing parameters

SI	65536
SF	400.1300000 MHz
WDW	EM
SSB	0
LB	0.30 Hz
GB	0
PC	1.00
