# Supplementary material for: Evolution of a Strategy for the Unified Synthesis of Enteropeptin Sactipeptides
Source: J Org Chem. 2026 Feb 23;91(9):3529–44. doi: 10.1021/acs.joc.5c03063 (PMC12973295; doi:10.1021/acs.joc.5c03063)
Supplement: Supplementary file 1 [file jo5c03063_si_001.zip › Compound 21 - 13C/2/pdata/1/email_yz-Z-MKG-OH-13C-DMSO_2_1.pdf]

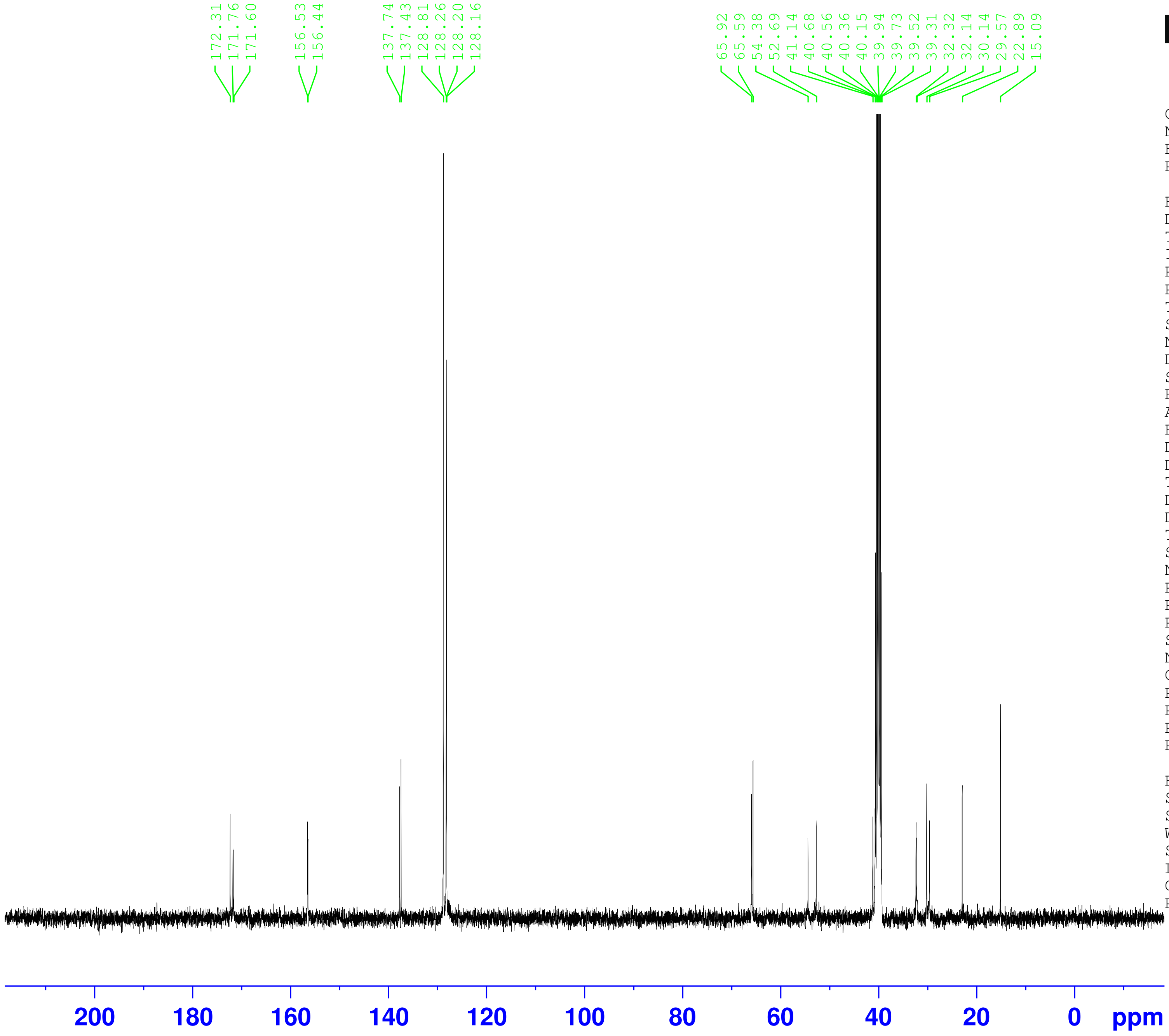


```
Current Data Parameters
NAME      yz-Z-MKG-OH-13C-DMSO
EXPNO                        2
PROCNO                       1

F2 - Acquisition Parameters
Date_                 20251121
Time                     18.03 h
INSTRUM                 Avance
PROBHD    Z163739_0940 (
PULPROG                 zgpg30
TD                       65536
SOLVENT                   DMSO
NS                         320
DS                           4
SWH                  23809.524 Hz
FIDRES                0.726609 Hz
AQ                   1.3762560 sec
RG                         101
DW                      21.000 usec
DE                        6.50 usec
TE                       298.0 K
D1                  2.00000000 sec
D11                 0.03000000 sec
TD0                          1
SFO1               100.6228298 MHz
NUC1                       13C
P0                        2.67 usec
P1                        8.00 usec
PLW1               87.00000000 W
SFO2               400.1316005 MHz
NUC2                        1H
CPDPRG[2               waltz65
PCPD2                    90.00 usec
PLW2               23.00000000 W
PLW12               0.18173000 W
PLW13               0.09140800 W

F2 - Processing parameters
SI                       32768
SF                 100.6127685 MHz
WDW                         EM
SSB                          0
LB                        1.00 Hz
GB                           0
PC                        1.40
```
