# Supplementary material for: Evolution of a Strategy for the Unified Synthesis of Enteropeptin Sactipeptides
Source: J Org Chem. 2026 Feb 23;91(9):3529–44. doi: 10.1021/acs.joc.5c03063 (PMC12973295; doi:10.1021/acs.joc.5c03063)
Supplement: Supplementary file 1 [file jo5c03063_si_001.zip › Compound 33 - 13C/1/pdata/1/email_yz-H-CPPS-13C-CDCl3_1_1.pdf]

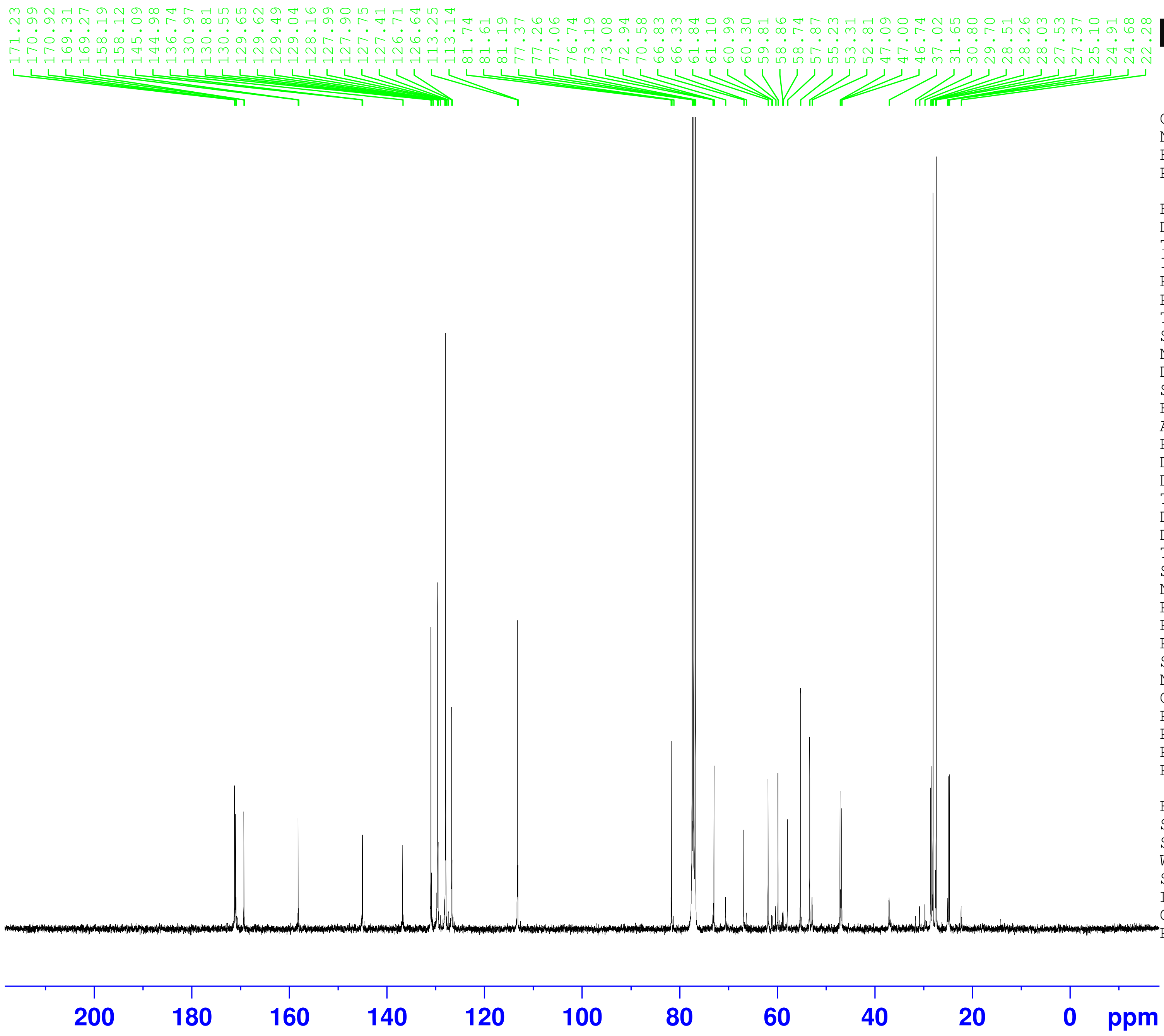

BRUKER

Current Data Parameters

NAME	yz-H-CPPS-13C-CDCl3
EXPNO	1
PROCNO	1

F2 - Acquisition Parameters

Date_	20251120	
Time	2.28	h
INSTRUM	Avance	
PROBHD	Z163739_0940 (	
PULPROG	zgpg30	
TD	65536	
SOLVENT	CDCl3	
NS	5600	
DS	4	
SWH	23809.524	Hz
FIDRES	0.726609	Hz
AQ	1.3762560	sec
RG	101	
DW	21.000	usec
DE	6.50	usec
TE	298.0	K
D1	2.00000000	sec
D11	0.03000000	sec
TD0	1	
SFO1	100.6228298	MHz
NUC1	13C	
P0	2.67	usec
P1	8.00	usec
PLW1	87.00000000	W
SFO2	400.1316005	MHz
NUC2	1H	
CPDPRG[2	waltz65	
PCPD2	90.00	usec
PLW2	23.00000000	W
PLW12	0.18173000	W
PLW13	0.09140800	W

F2 - Processing parameters

SI	32768	
SF	100.6127685	MHz
WDW	EM	
SSB	0	
LB	1.00	Hz
GB	0	
PC	1.40	
