# Supplementary material for: Evolution of a Strategy for the Unified Synthesis of Enteropeptin Sactipeptides
Source: J Org Chem. 2026 Feb 23;91(9):3529–44. doi: 10.1021/acs.joc.5c03063 (PMC12973295; doi:10.1021/acs.joc.5c03063)
Supplement: Supplementary file 1 [file jo5c03063_si_001.zip › Compound 34 - 1H/5/pdata/1/email_yz-MKGOC(mmt)PPS-1H-CD3OD-2_5_1.pdf]

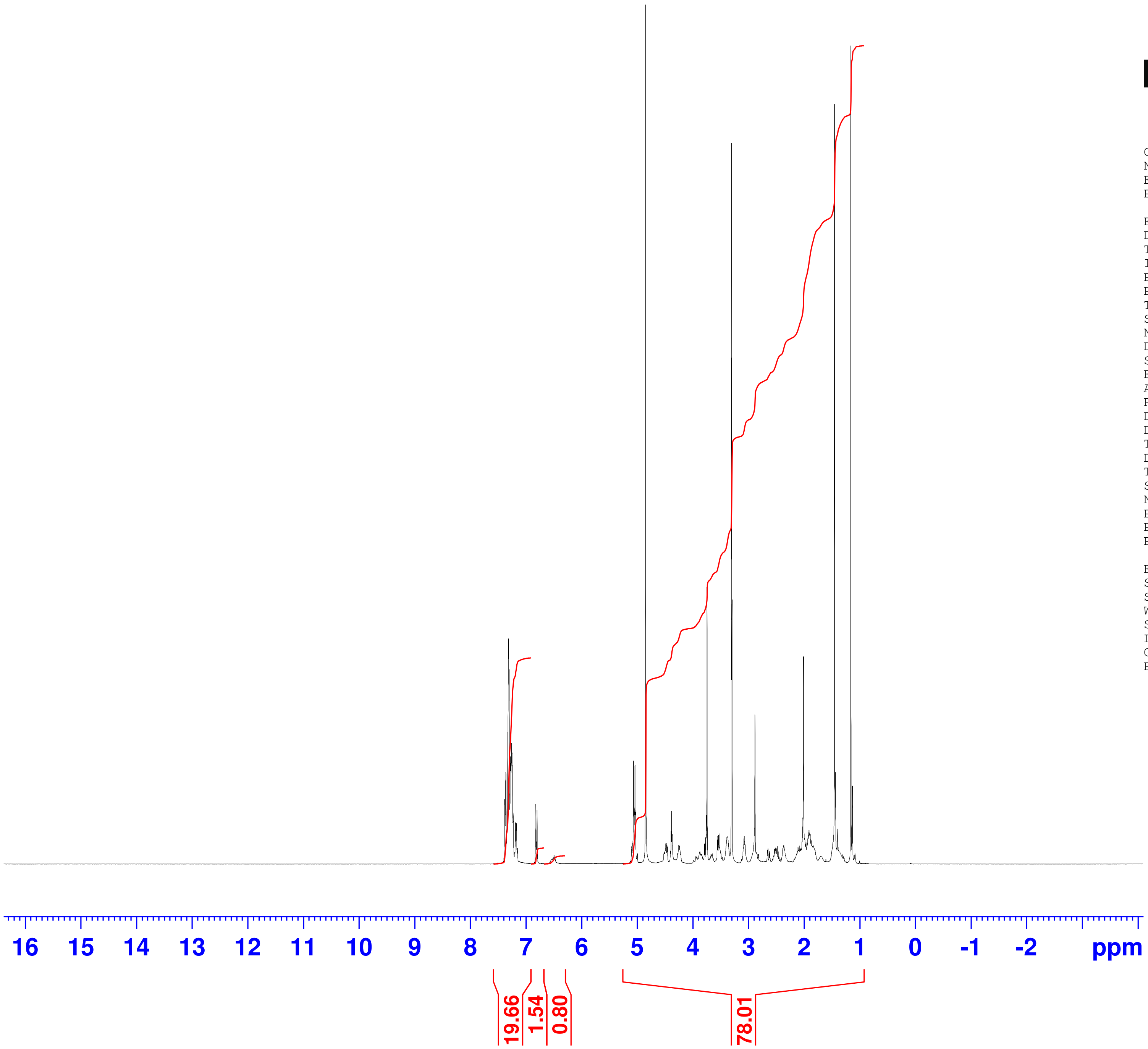

BRUKER

Current Data Parameters
NAME yz-MKGOC(mmt)PPS-1H-CD3OD-2
EXPNO 5
PROCNO 1

F2 - Acquisition Parameters

Date_	20251023	
Time	19.21	h
INSTRUM	Avance	
PROBHD	Z163739_0940 (	
PULPROG	zg30	
TD	65536	
SOLVENT	CD3OD_SPE	
NS	64	
DS	2	
SWH	8196.721	Hz
FIDRES	0.250144	Hz
AQ	3.9976959	sec
RG	101	
DW	61.000	usec
DE	13.89	usec
TE	298.0	K
D1	1.00000000	sec
TD0	1	
SFO1	400.1324708	MHz
NUC1	1H	
P0	2.67	usec
P1	8.00	usec
PLW1	23.00000000	W

F2 - Processing parameters

SI	65536	
SF	400.1300117	MHz
WDW	EM	
SSB	0	
LB	0.30	Hz
GB	0	
PC	1.00	
