# Supplementary material for: Evolution of a Strategy for the Unified Synthesis of Enteropeptin Sactipeptides
Source: J Org Chem. 2026 Feb 23;91(9):3529–44. doi: 10.1021/acs.joc.5c03063 (PMC12973295; doi:10.1021/acs.joc.5c03063)
Supplement: Supplementary file 2 [file jo5c03063_si_002.pdf]

Supporting Information

Evolution of a Strategy for the Unified Synthesis of Enteropeptin Sactipeptides

Shuvendu Saha†, Yiwei Zhang†, Yesen Cheng, Chi P. Ting*

Brandeis University, Department of Chemistry, 415 South St. Waltham, MA 02453, United States

chiting@brandeis.edu

†These authors contributed equally to this manuscript.

Supporting Information

Table of Contents:

Microwave Synthesis and Temperature Control……......…………………….............................S2

NMR spectra……......…………………………………………..…………………………..S3

A CEM Mars 6 Microwave Synthesizer instrument (CEM Corporation) was used for microwave irradiation. Microwave synthesis was conducted in microwave reactor with 20 mL sealed microwave reaction vessels (GlassChem 20 vessel). The reaction temperature is monitored by the MTS-300 temperature fiber optic probe from CEM Corporation.

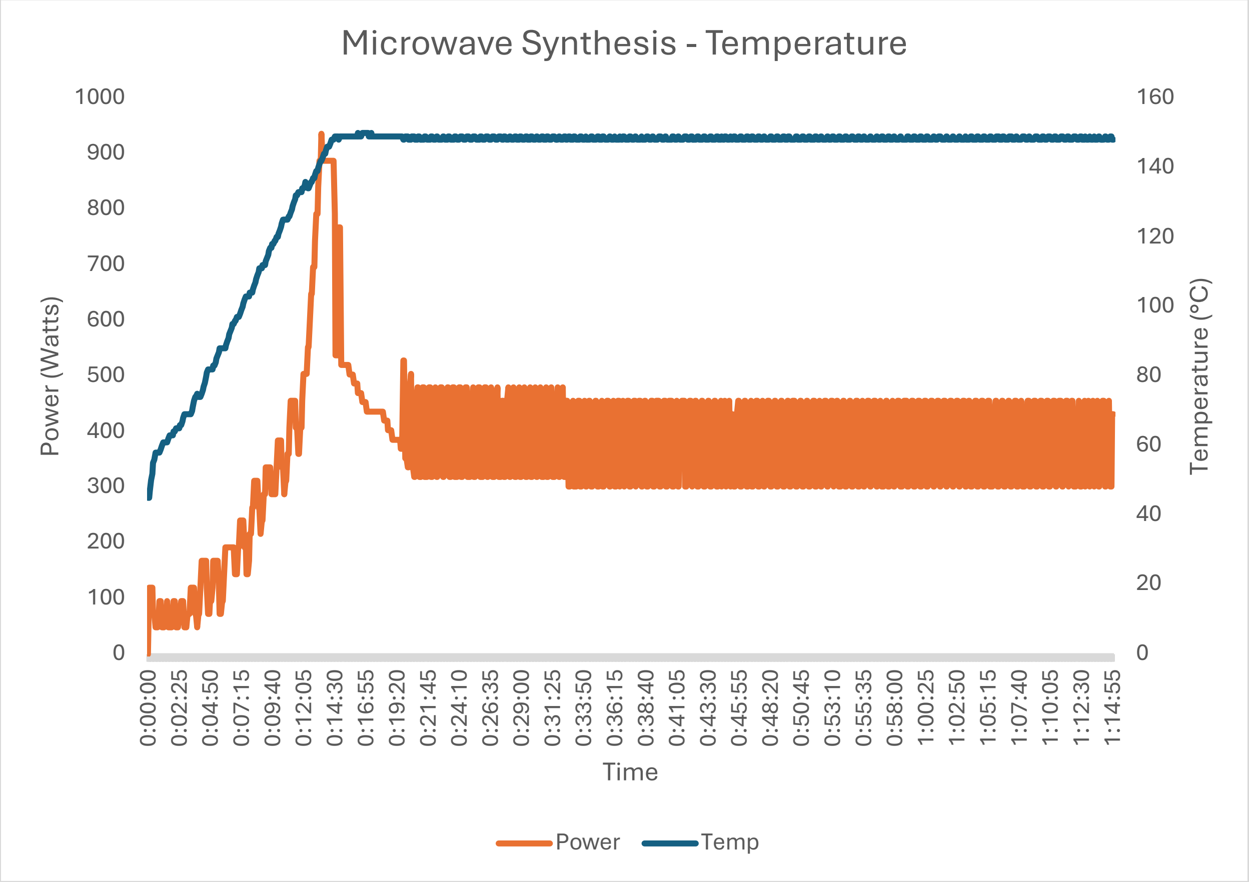

Figure S1. Temperature Profile for Microwave Synthesis.

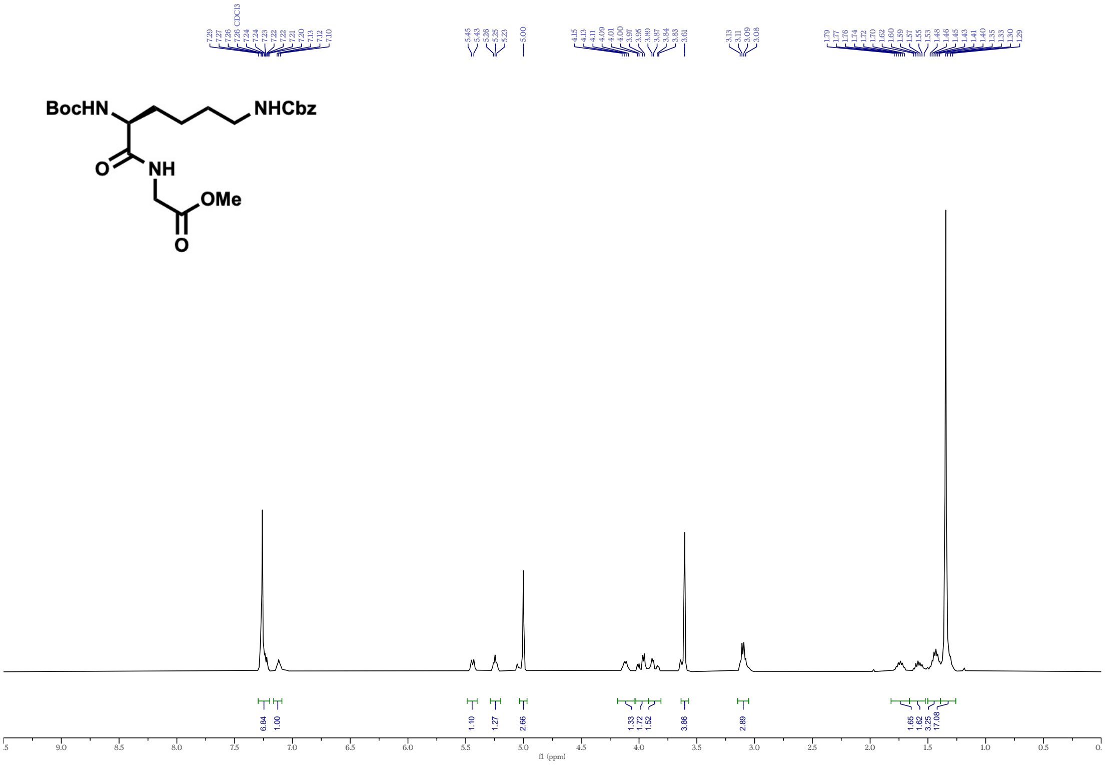

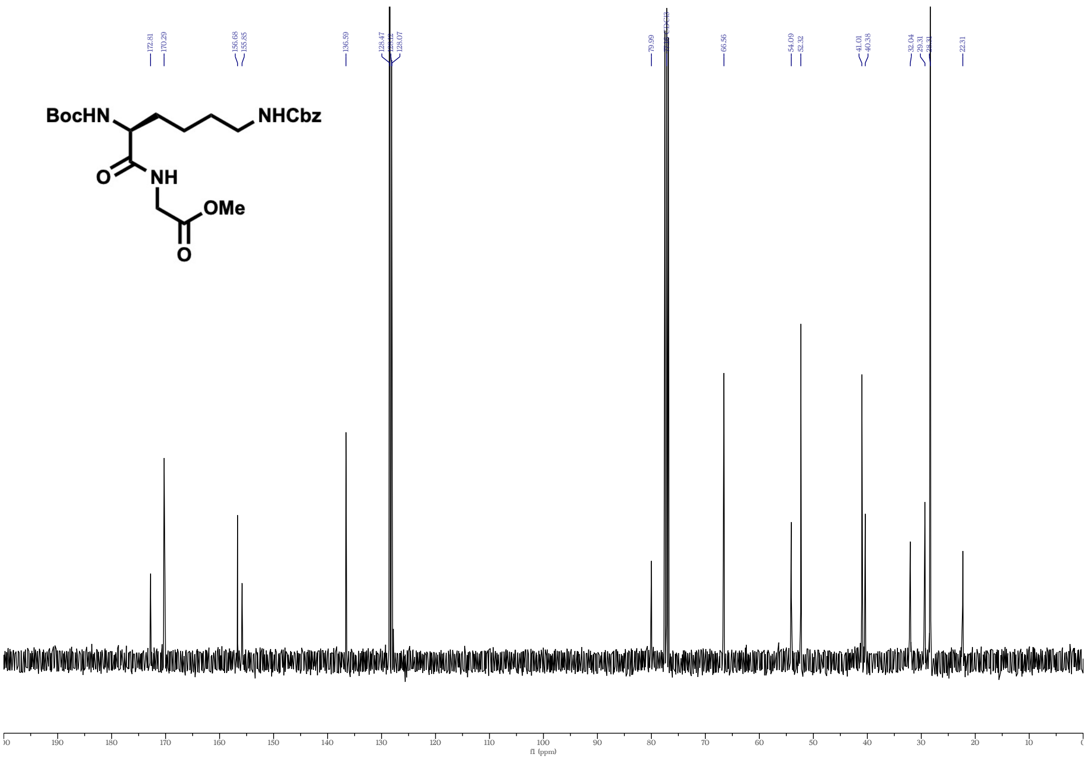

^{1}H NMR (400 MHz) and $^{13}C\{^{1}H\}$ NMR (100 MHz) Spectra of **20** in $CDCl_3$.

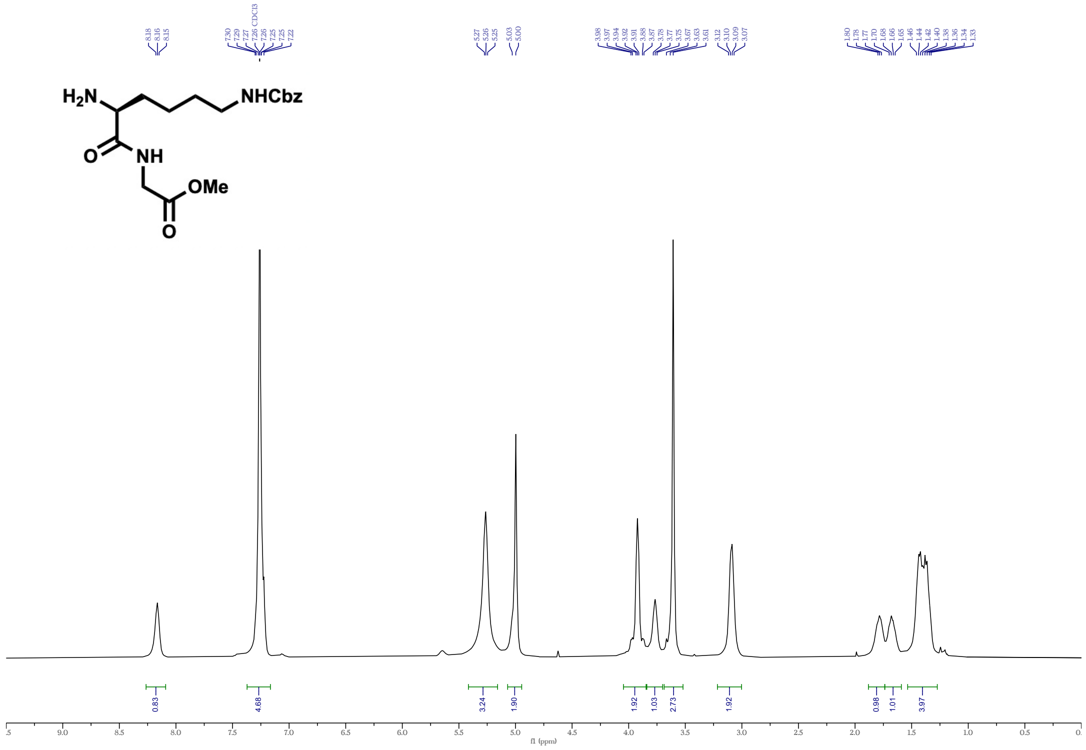

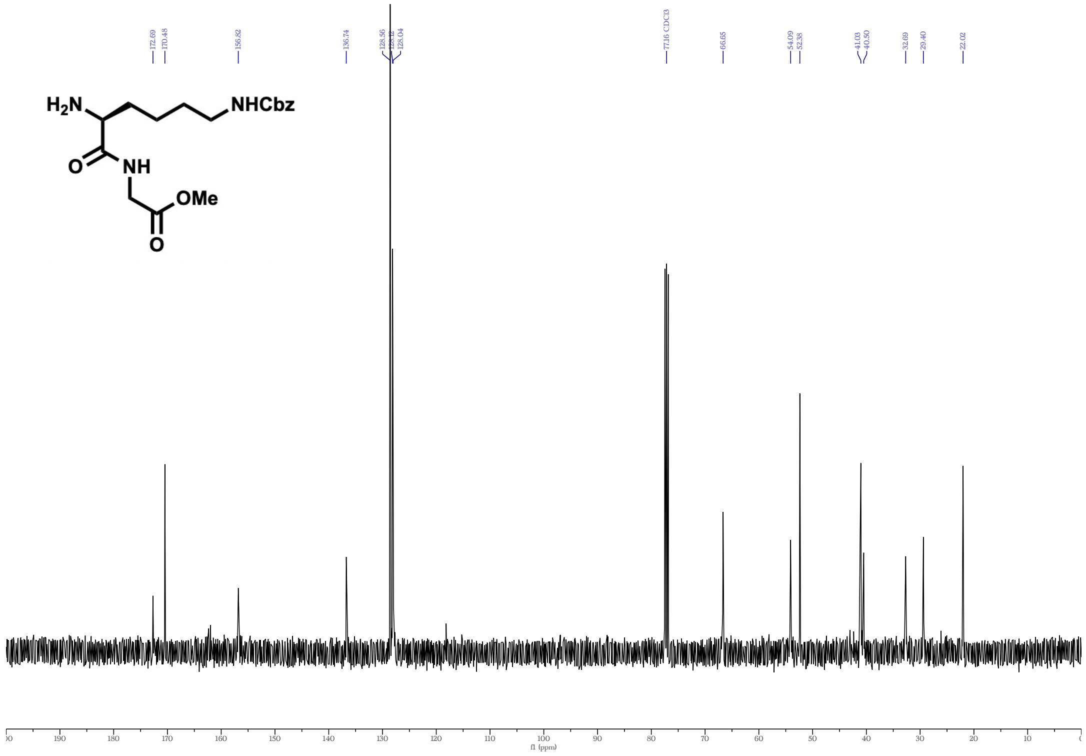

^{1}H NMR (400 MHz) and $^{13}C\{^{1}H\}$ NMR (100 MHz) Spectra of **16** in $CDCl_3$.

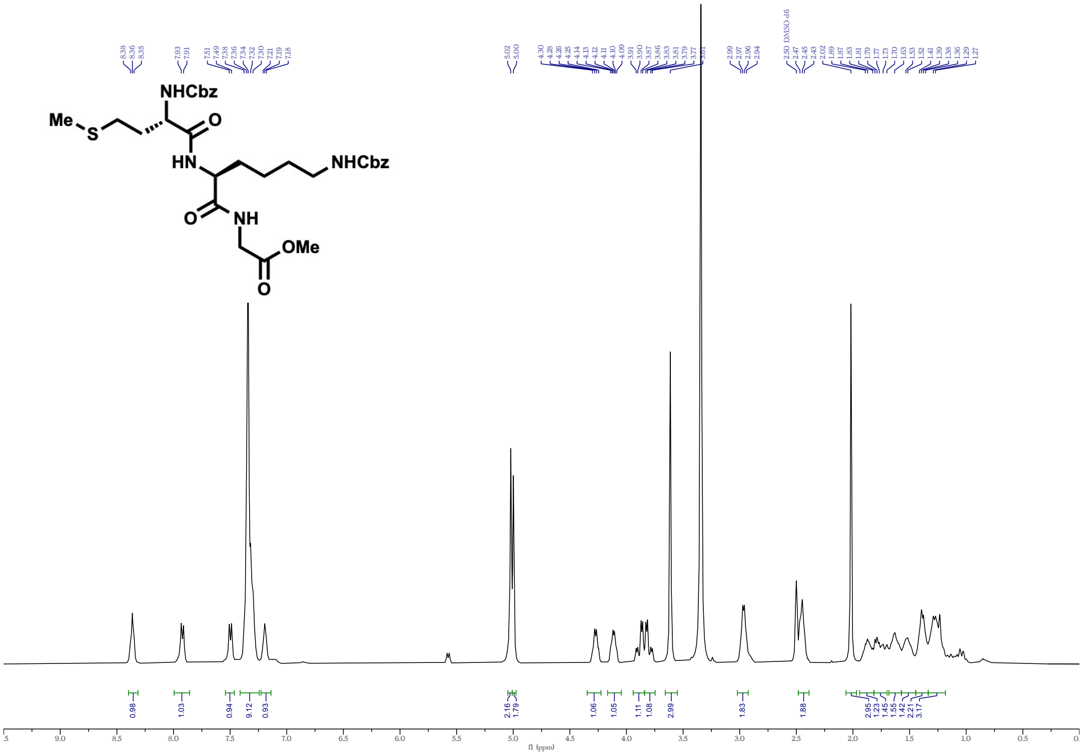

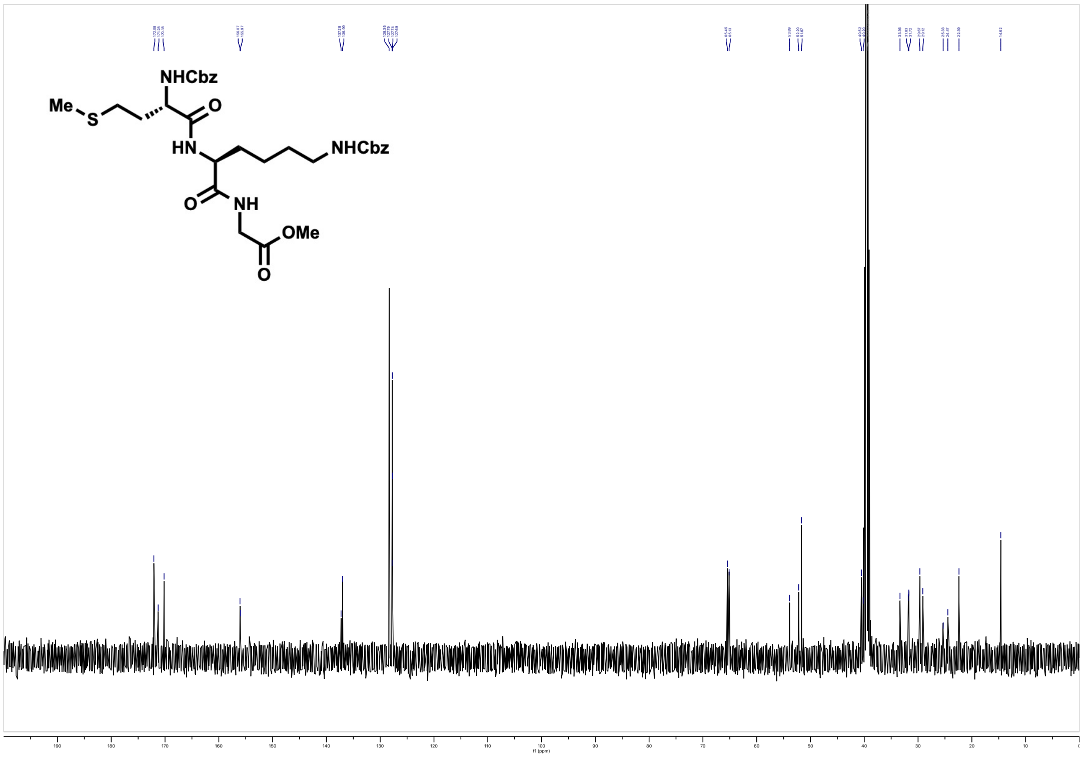

^{1}H NMR (400 MHz) and $^{13}C\{^{1}H\}$ NMR (100 MHz) Spectra of **17** in d_6-DMSO.

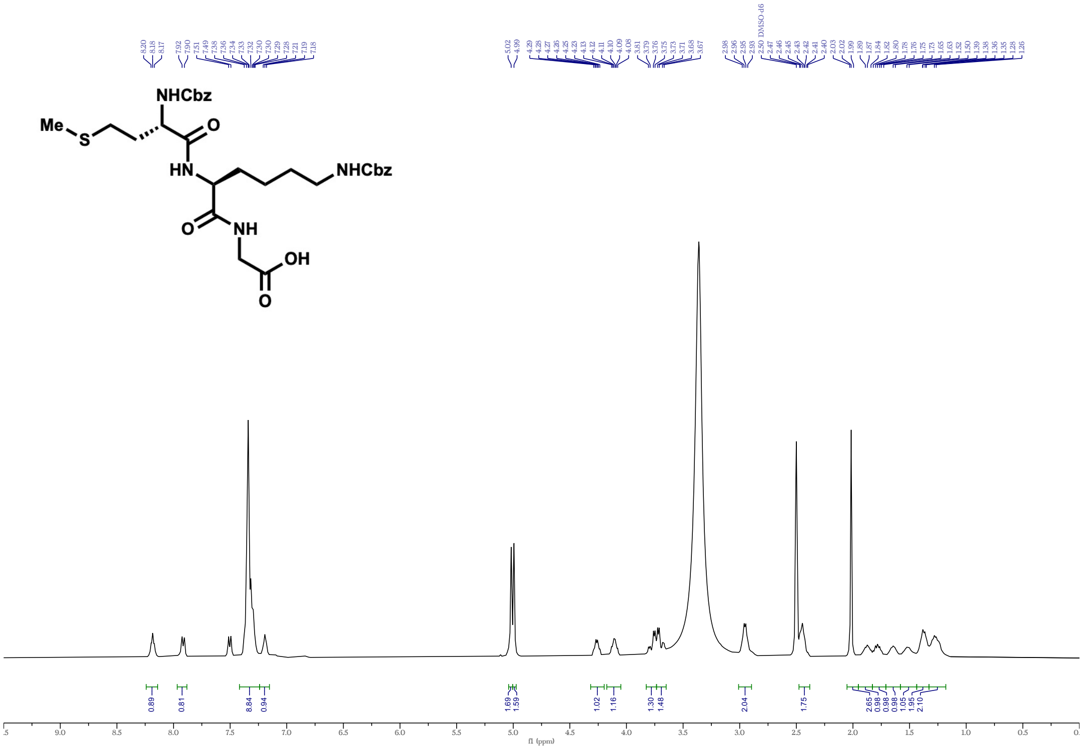

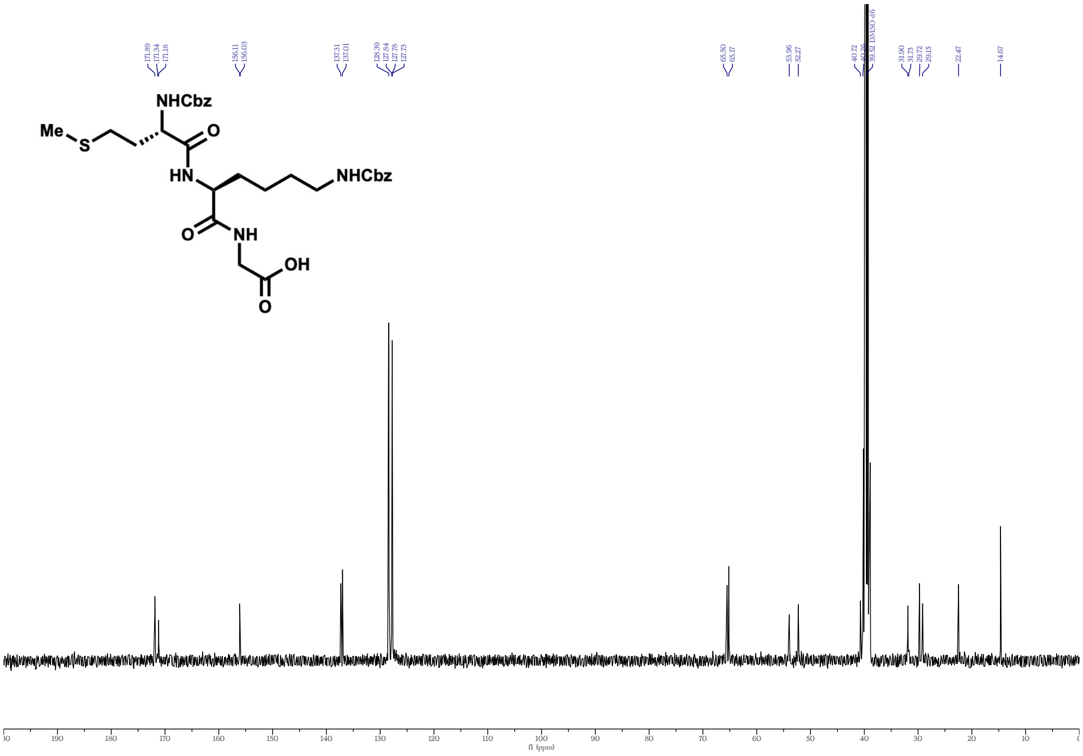

^{1}H NMR (400 MHz) and $^{13}C\{^{1}H\}$ (100 MHz) NMR Spectra of **21** in d_6-DMSO.

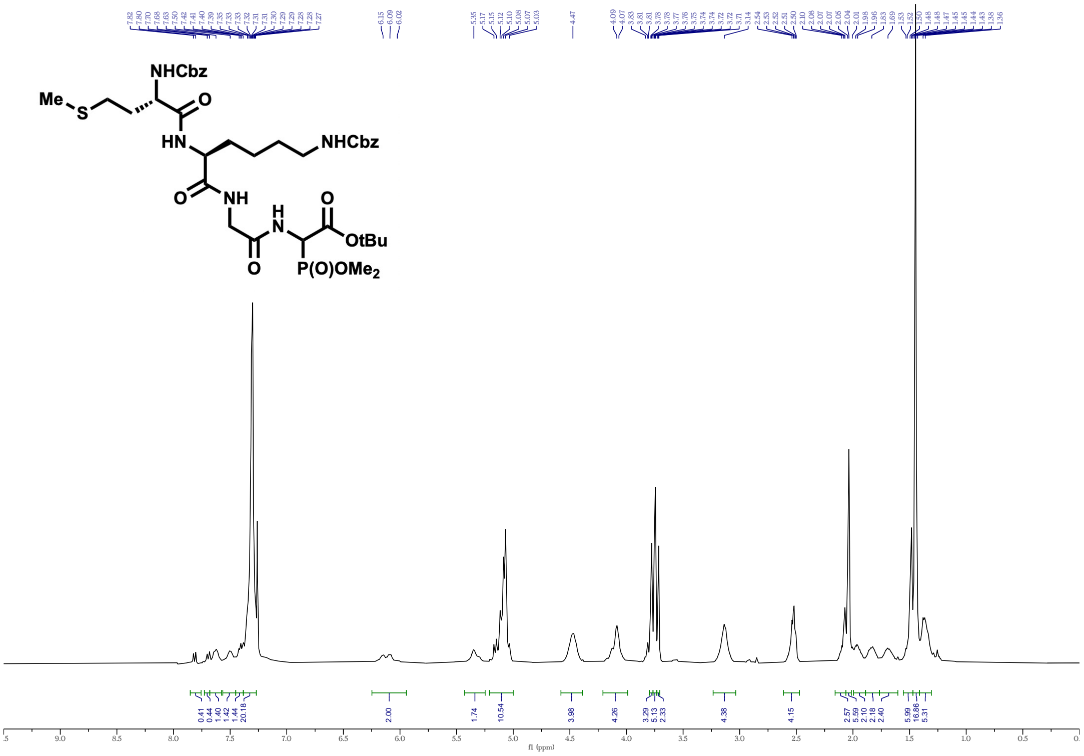

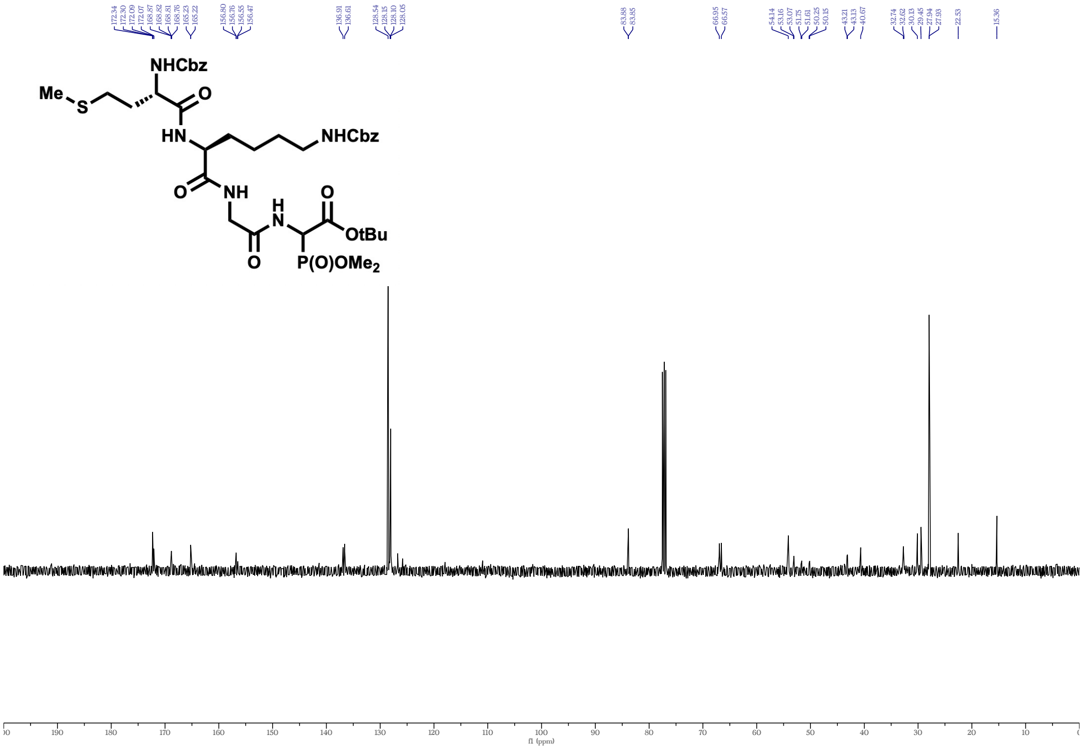

^{1}H NMR (400 MHz) and $^{13}C\{^1H\}$ (100 MHz) NMR Spectra of **23** in $CDCl_3$.

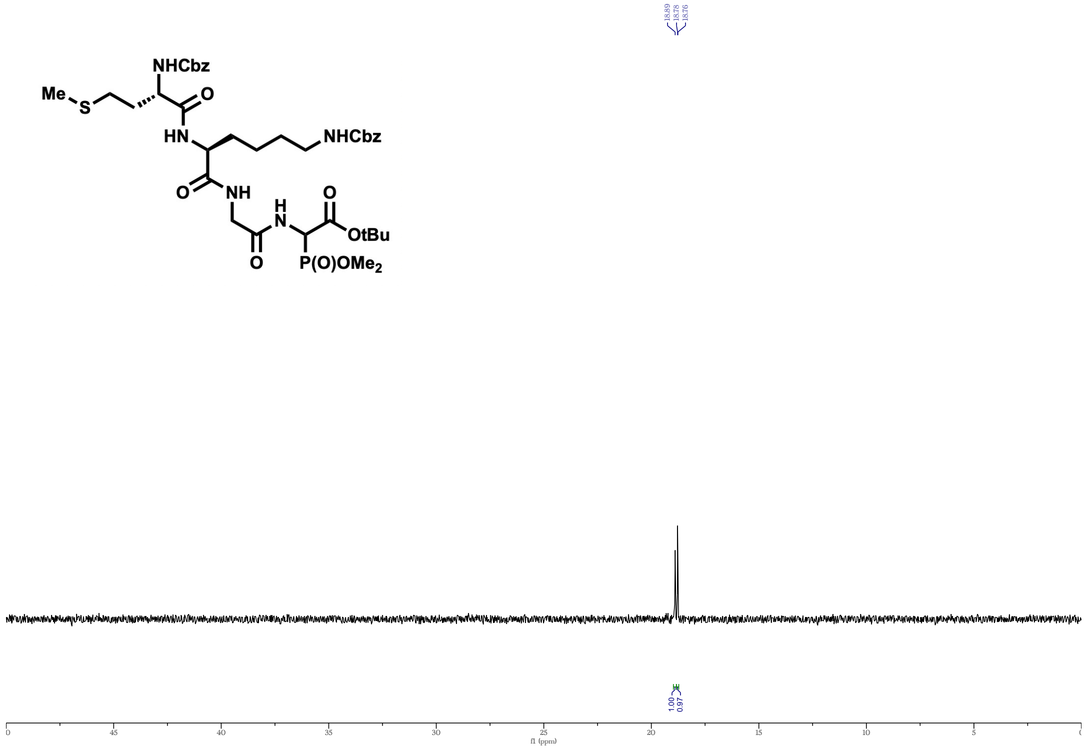

$^{31}P\{^{1}H\}$ NMR (162 MHz) Spectra of **23** in $CDCl_3$.

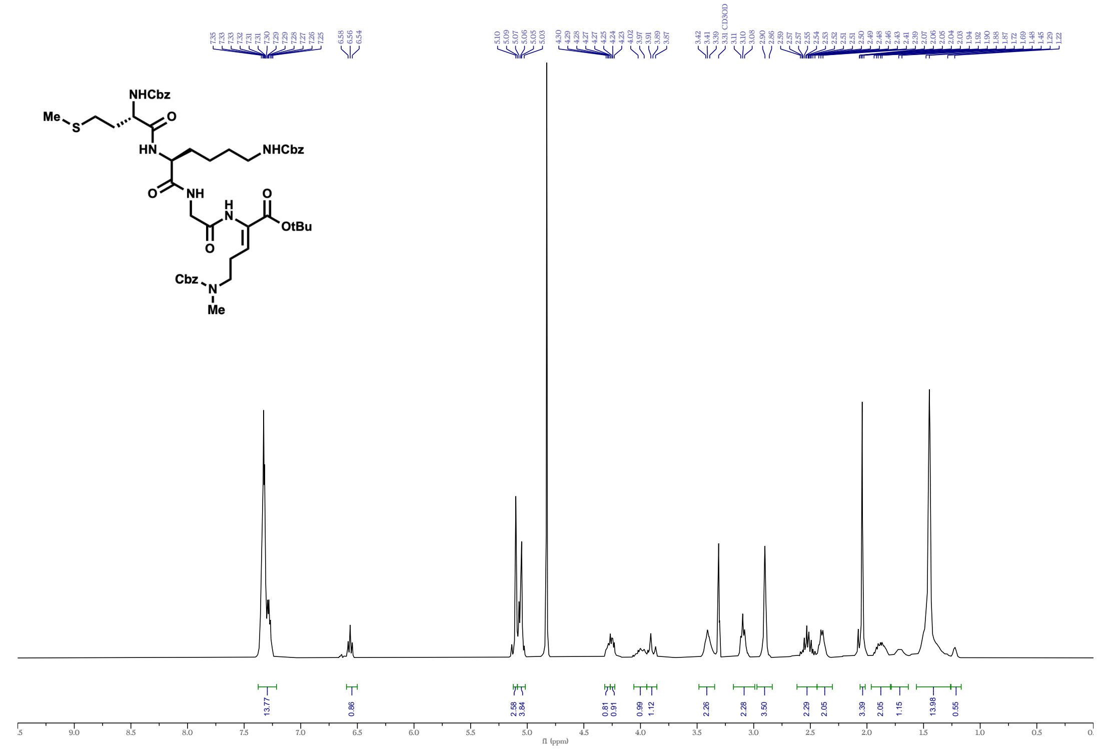

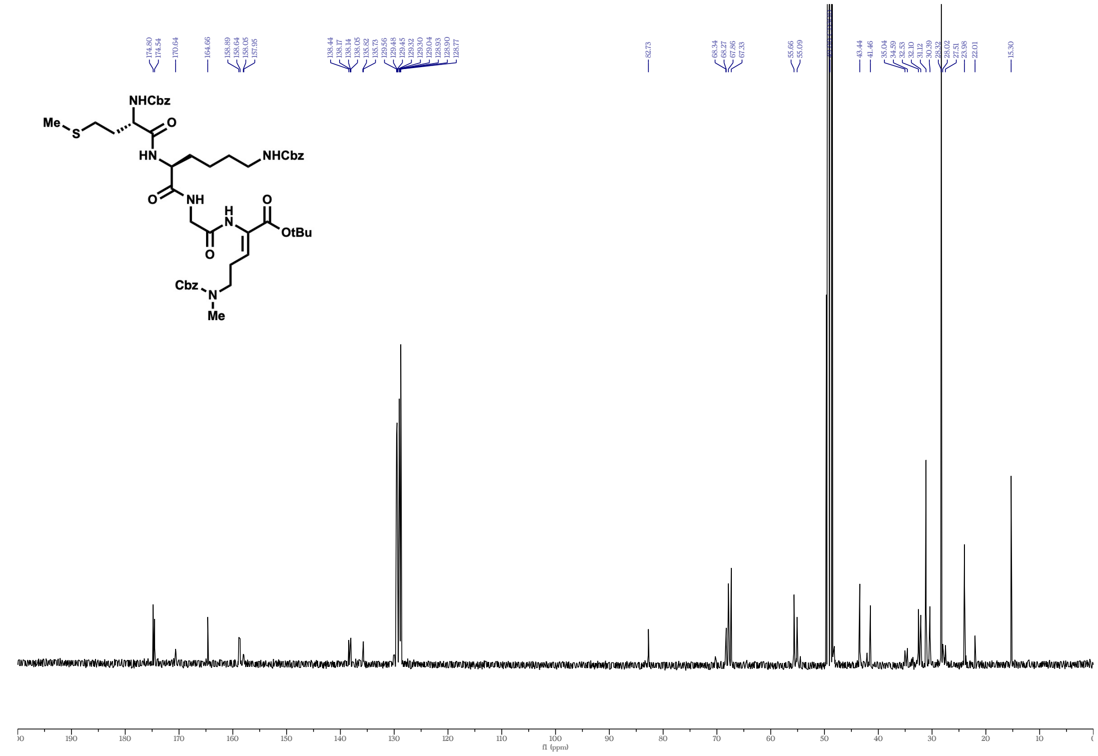

^{1}H NMR (400 MHz) and $^{13}C\{^{1}H\}$ NMR (100 MHz) Spectra of **25** in CD_3OD.

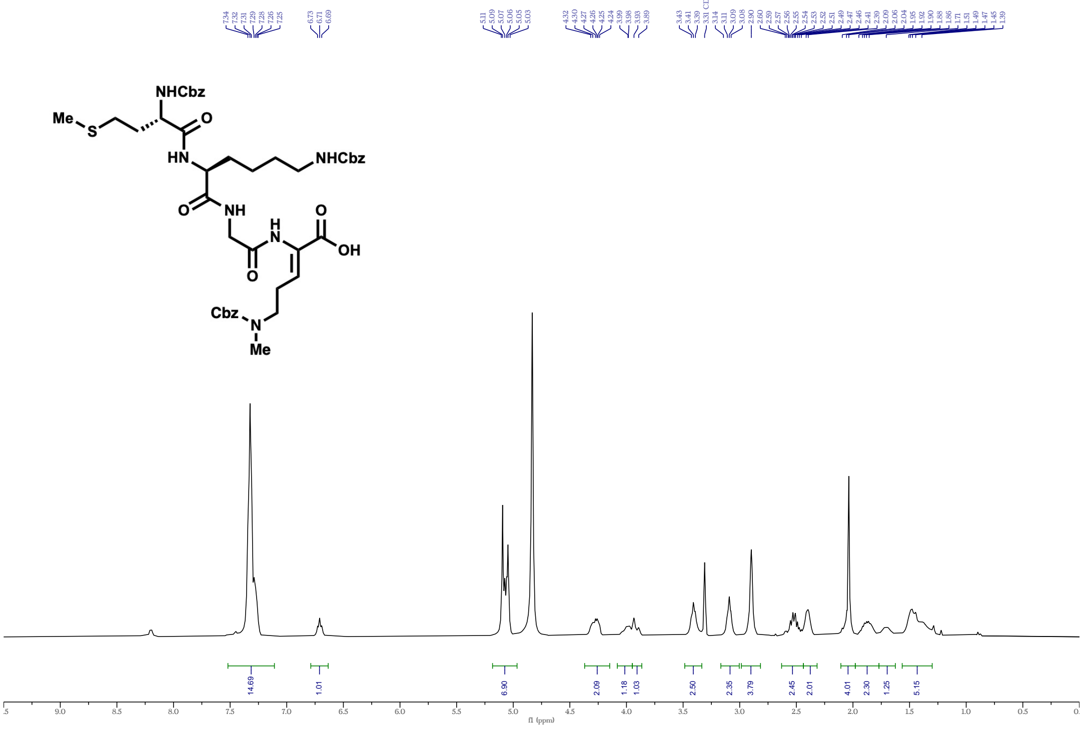

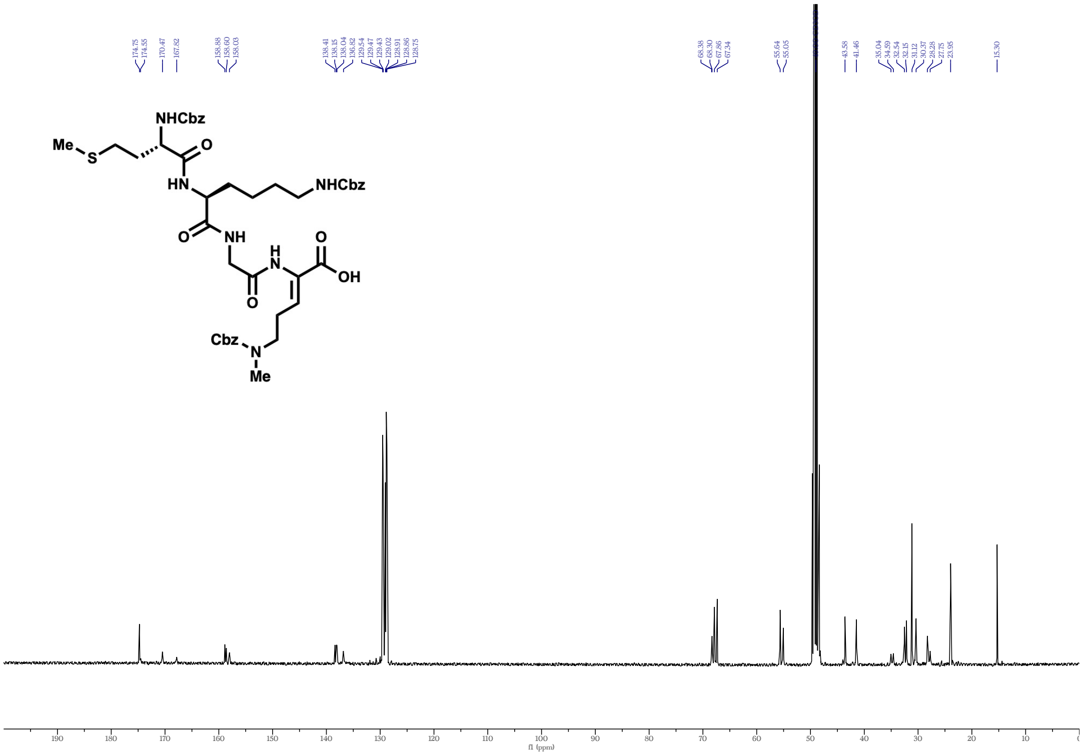

^{1}H NMR (400 MHz) and $^{13}C\{^{1}H\}$ NMR (100 MHz) Spectra of **26** in CD_3OD.

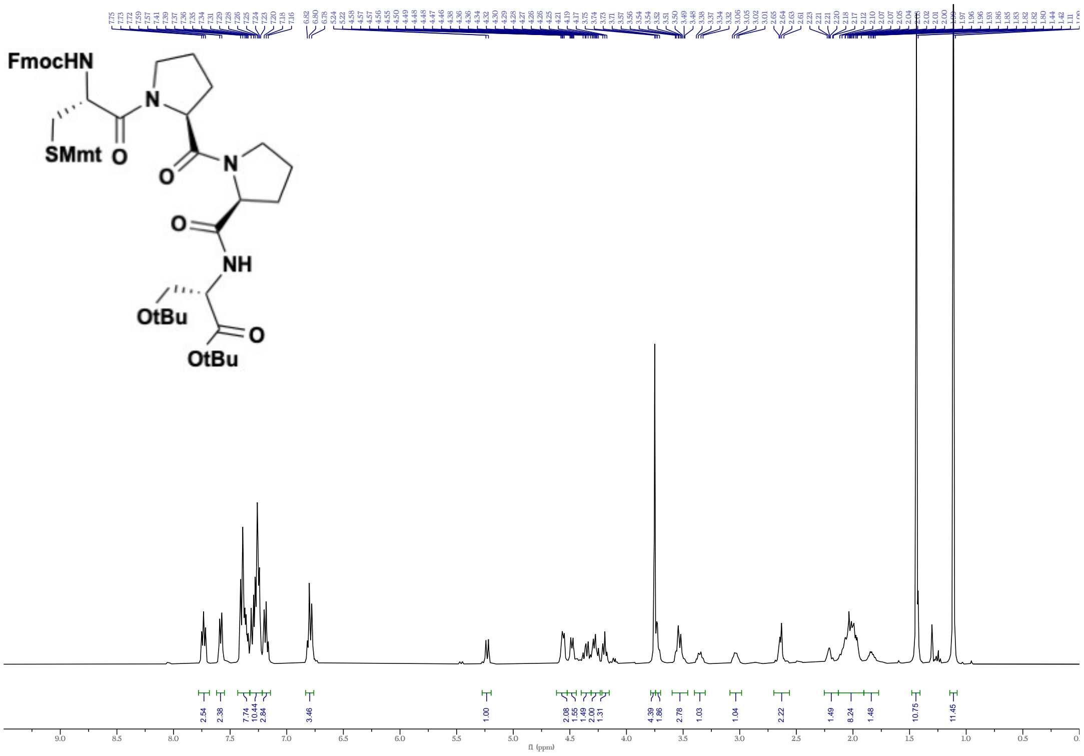

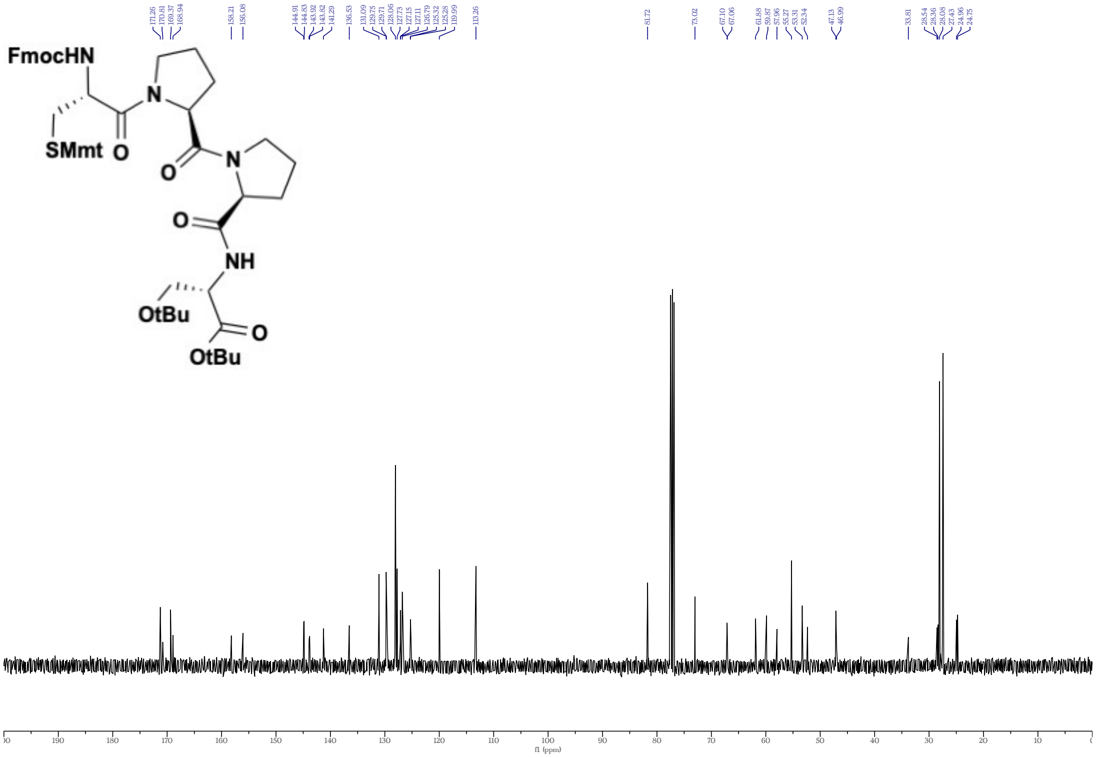

^{1}H NMR (400 MHz) and ^{13}C{^{1}H} NMR (100 MHz) Spectra of **32** in $CDCl_3$.

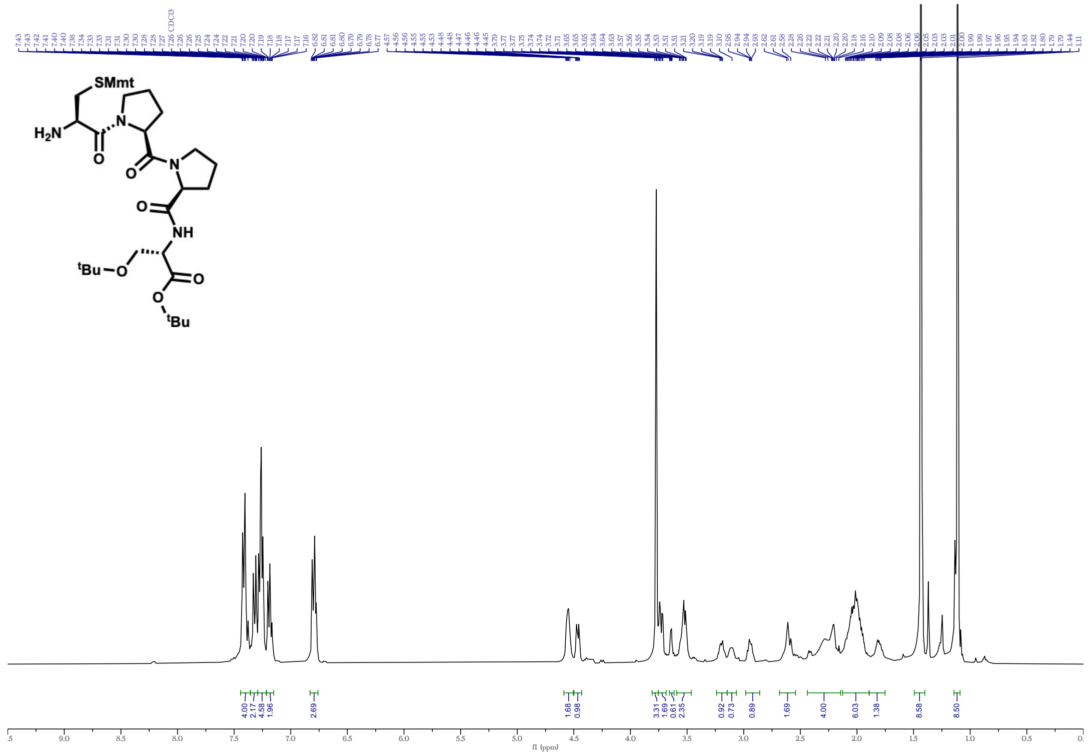

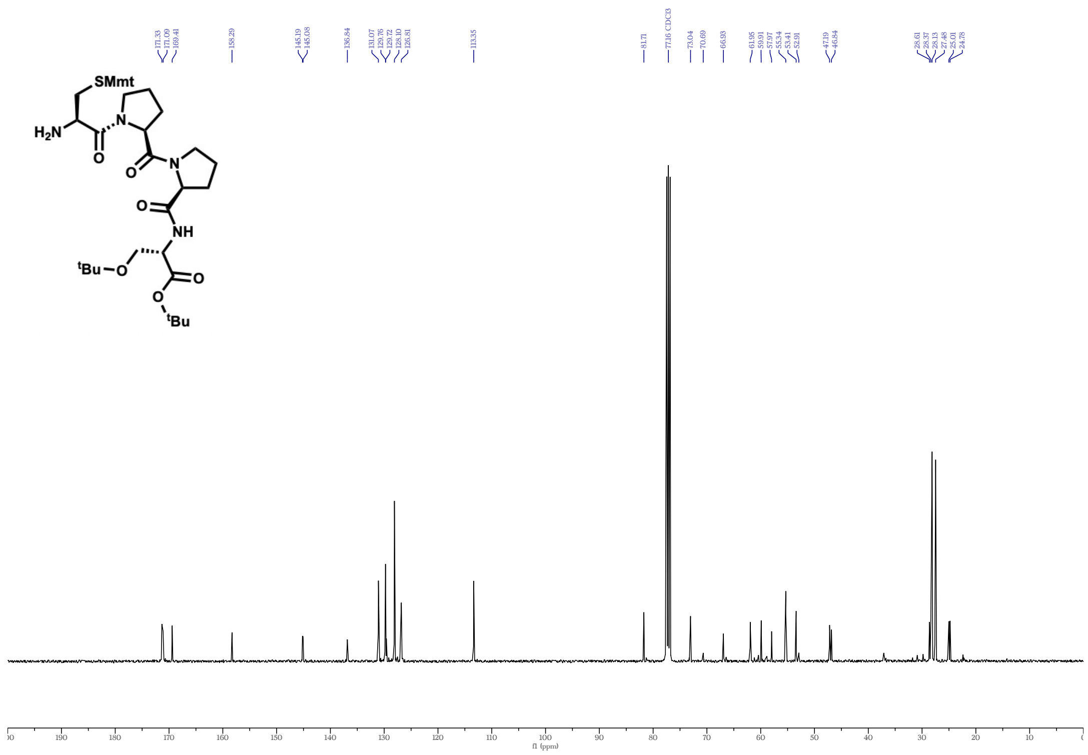

^{1}H NMR (400 MHz) and $^{13}C\{^{1}H\}$ NMR (100 MHz) Spectra of **33** in $CDCl_3$.

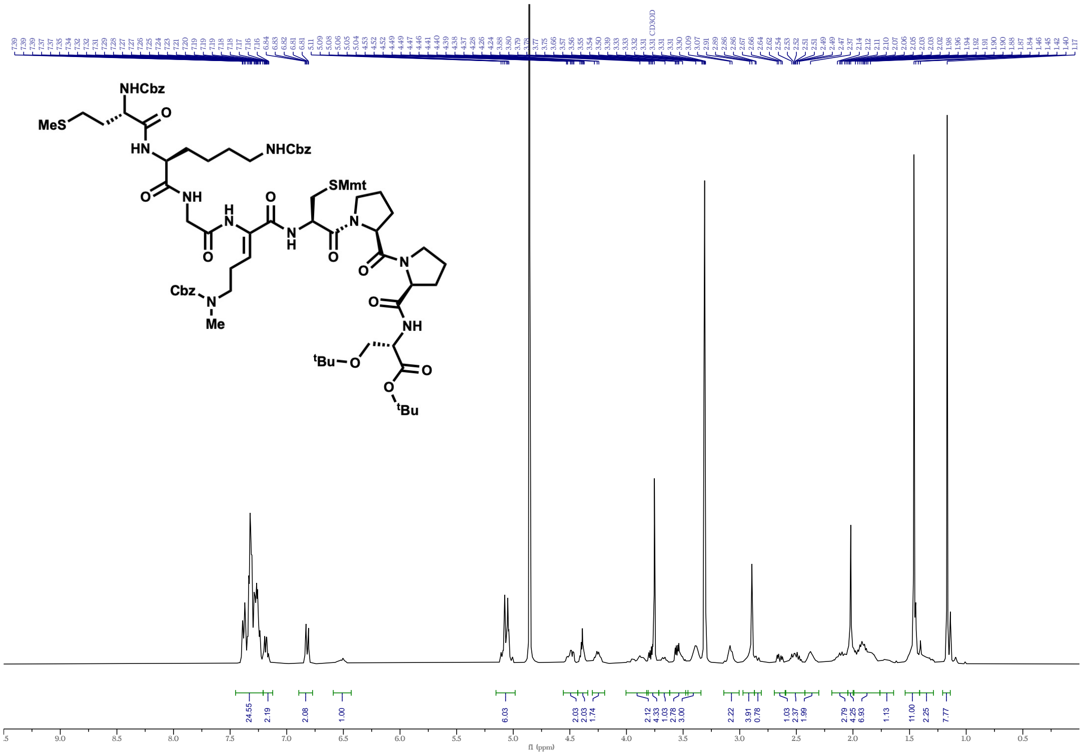

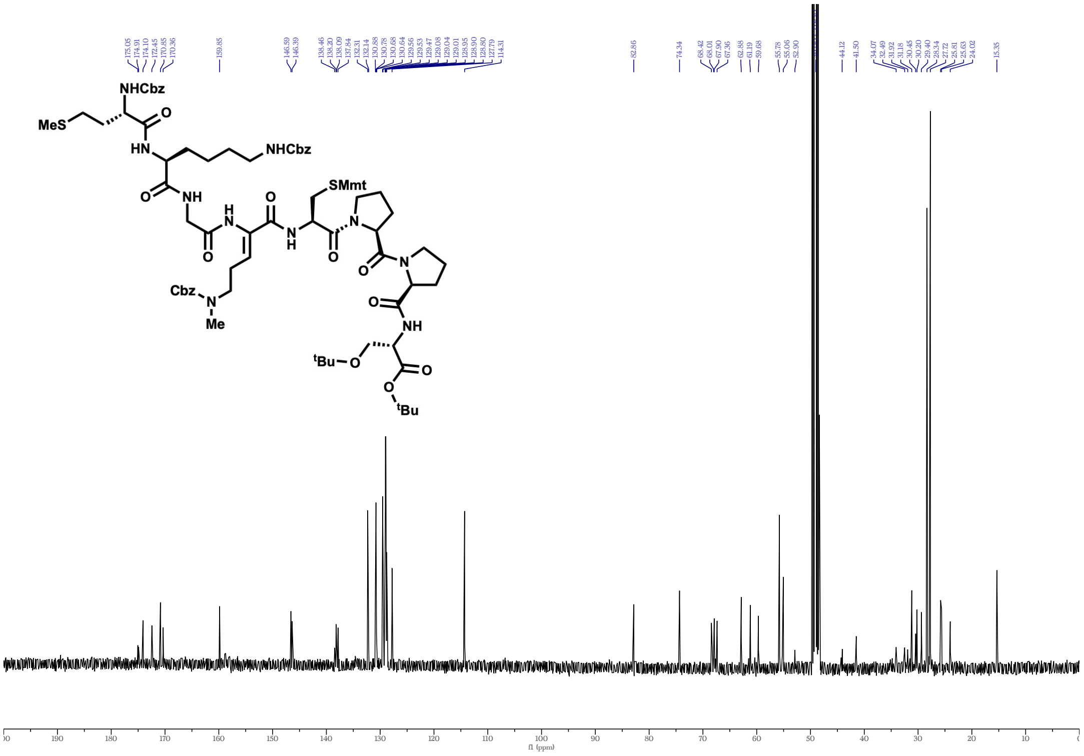

^{1}H NMR (400 MHz) and ^{13}C{^{1}H} NMR (100 MHz) Spectra of **34** in CD_3OD.

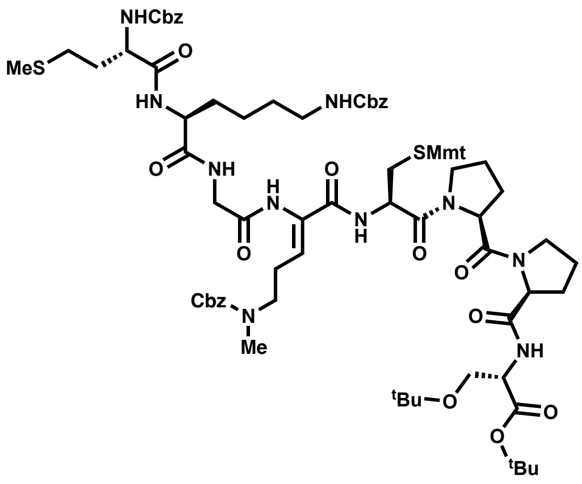

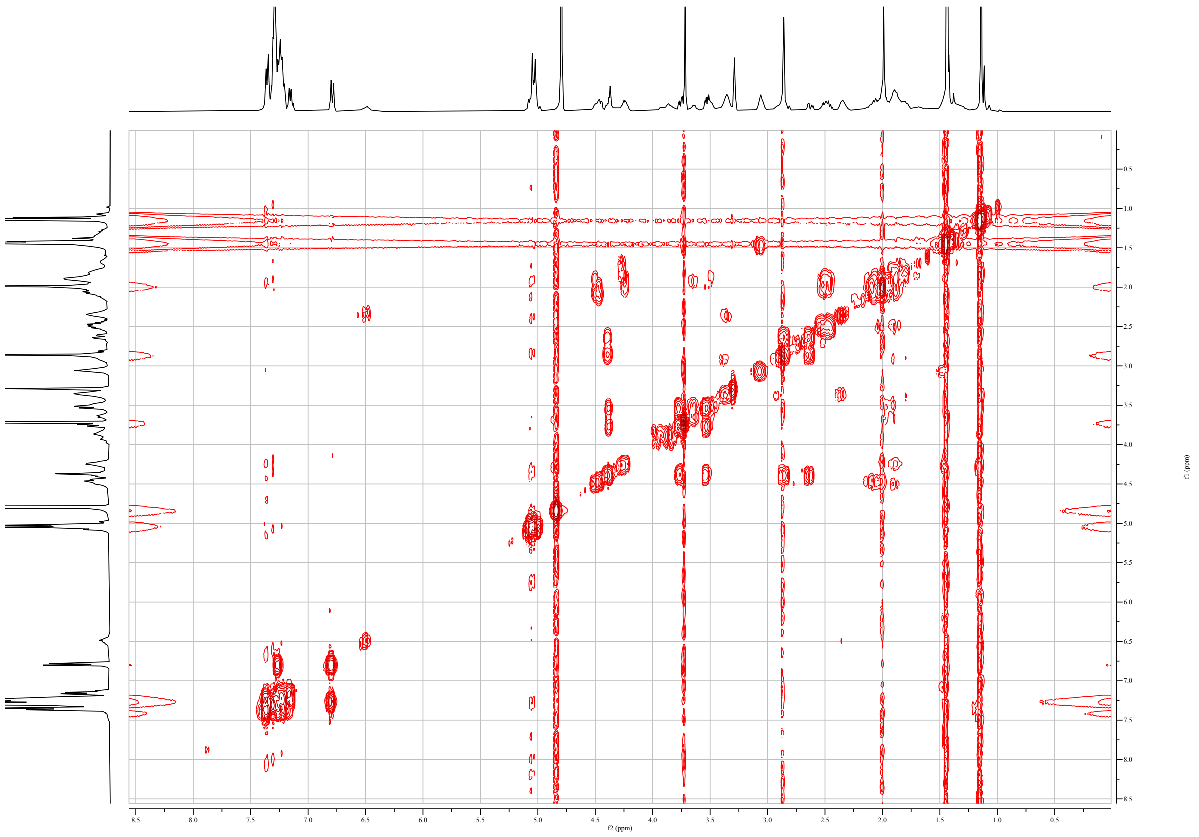

2D COSY NMR (400 MHz) Spectrum of **34** in CD_3OD.

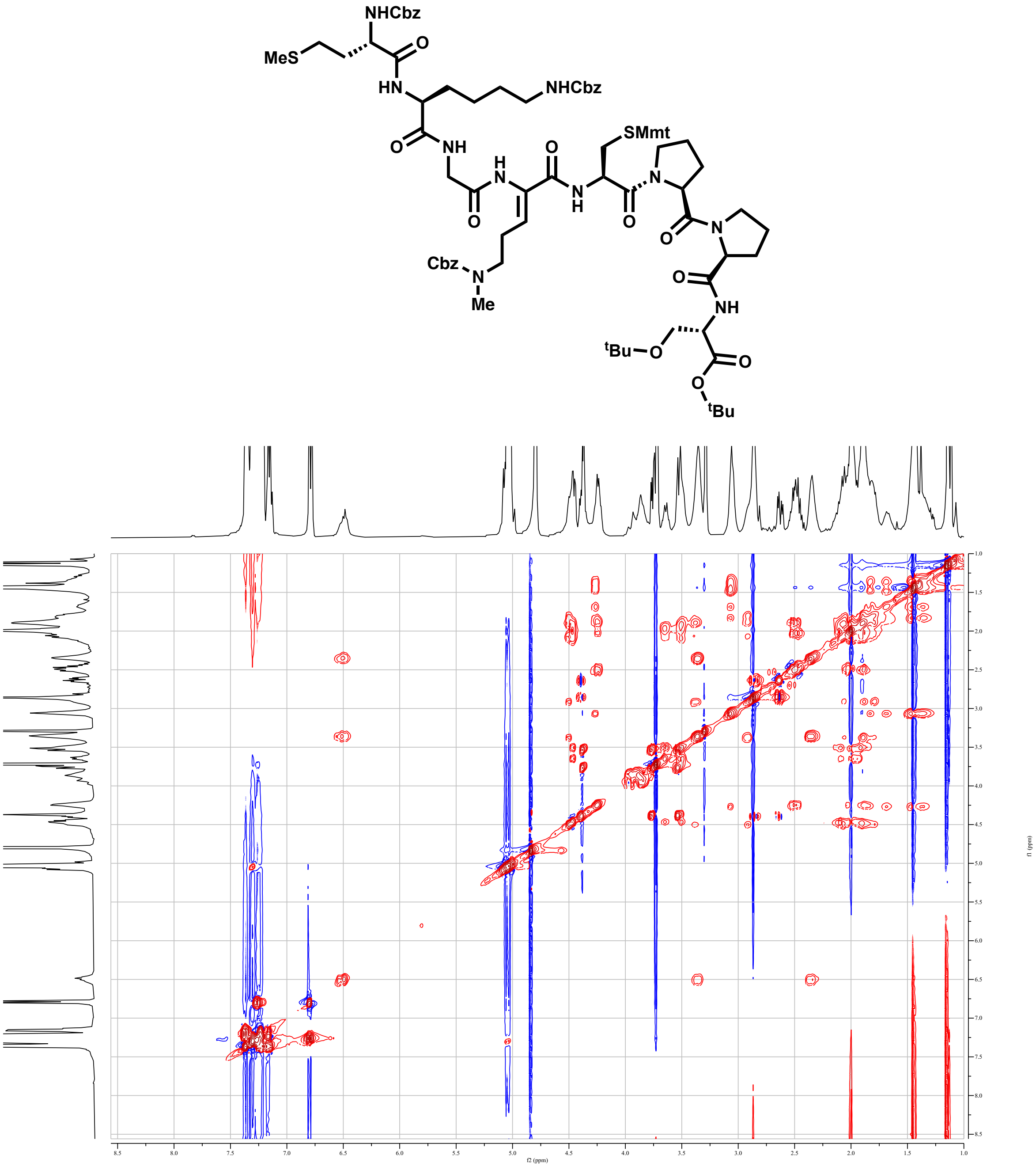

2D TOCSY NMR (400 MHz) Spectrum of **34** in CD_3OD.

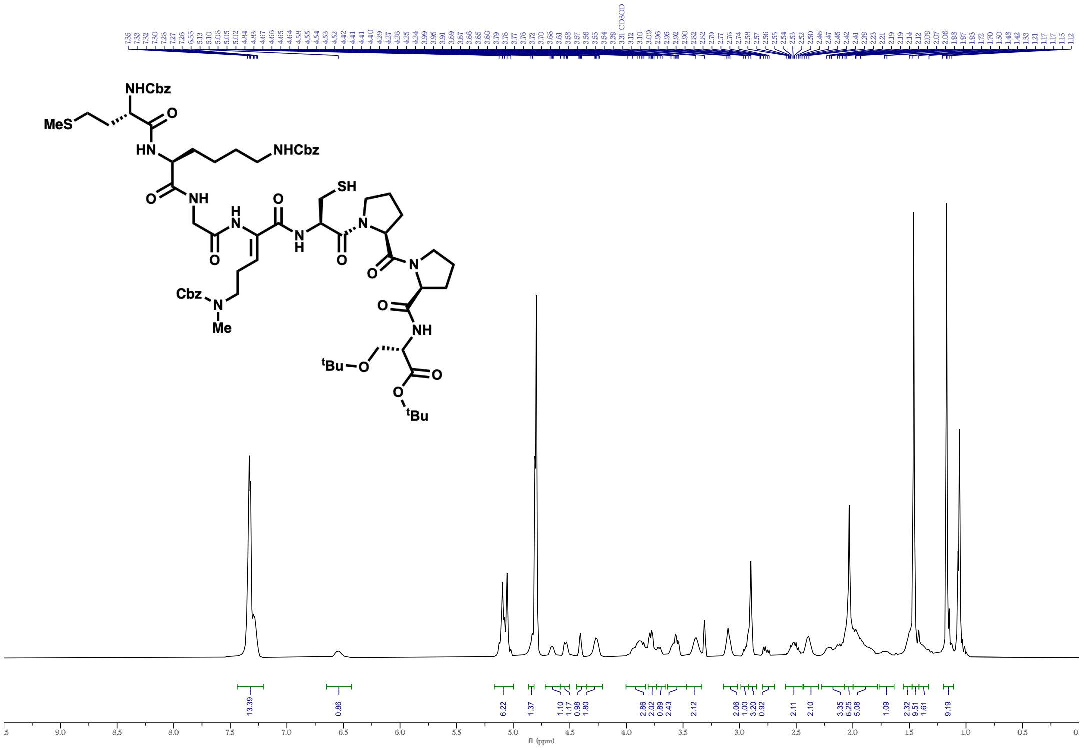

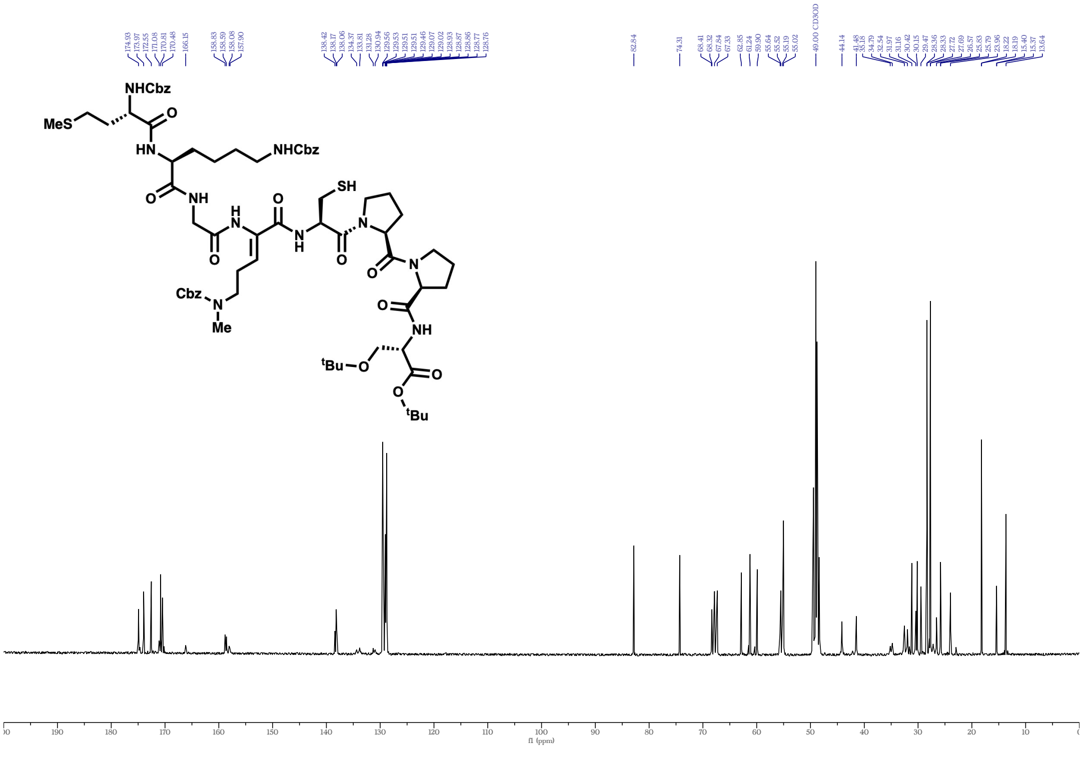

^{1}H and $^{13}C\{^{1}H\}$ NMR (400 MHz) Spectra of **35** in CD_3OD.

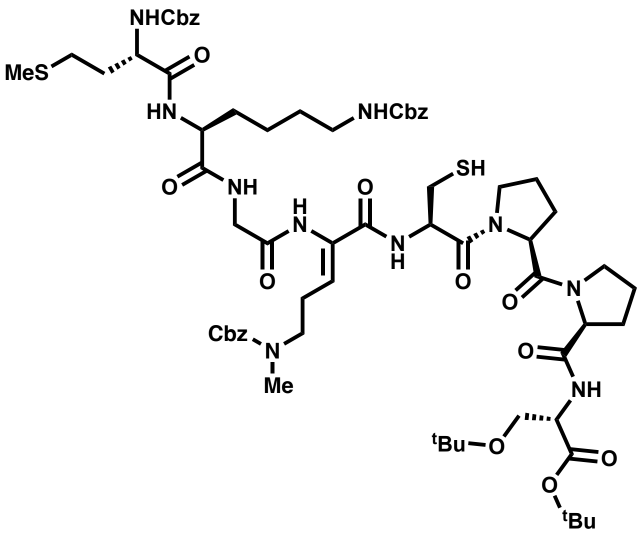

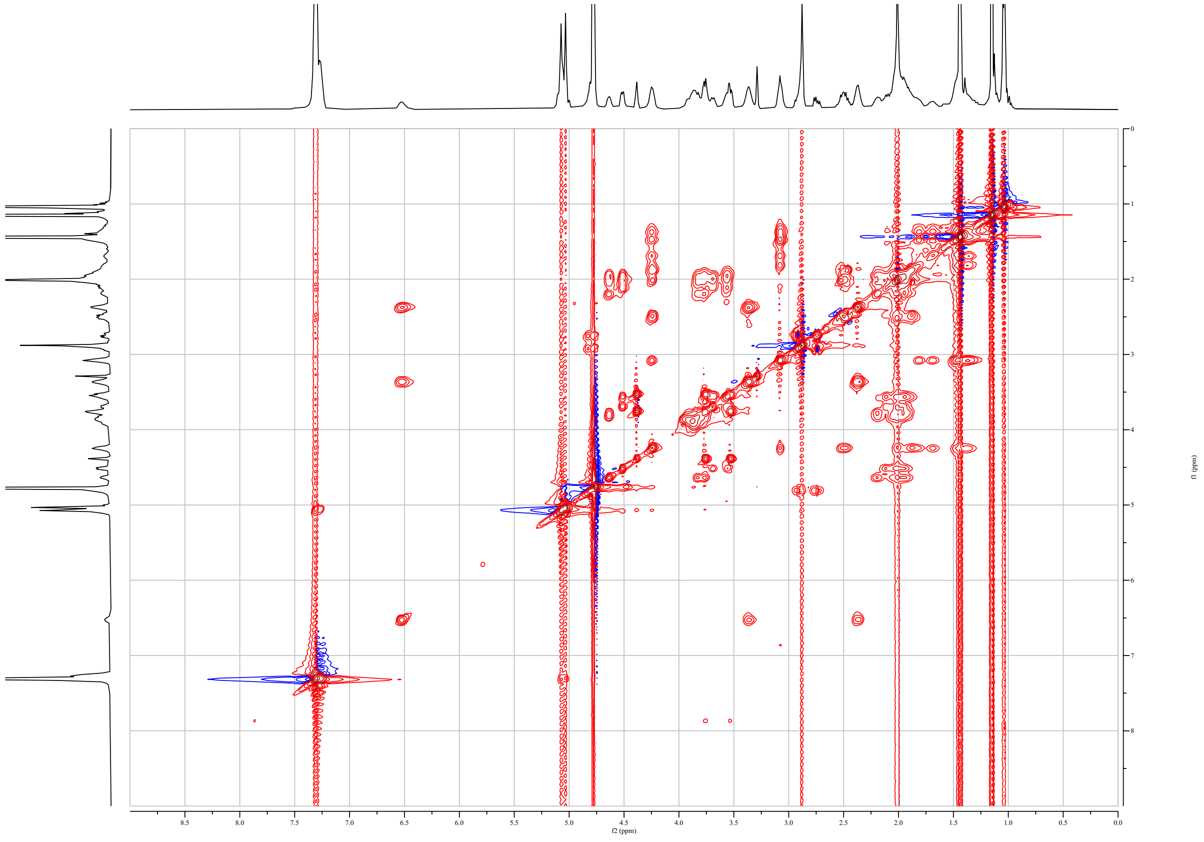

2D COSY NMR (400 MHz) Spectrum of **35** in CD_3OD.

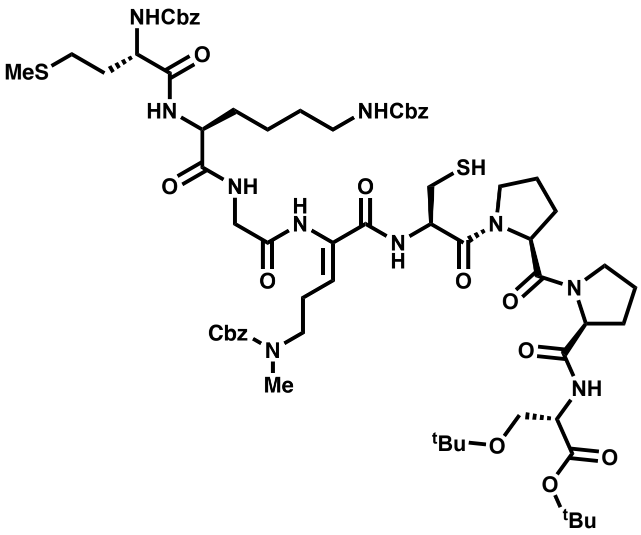

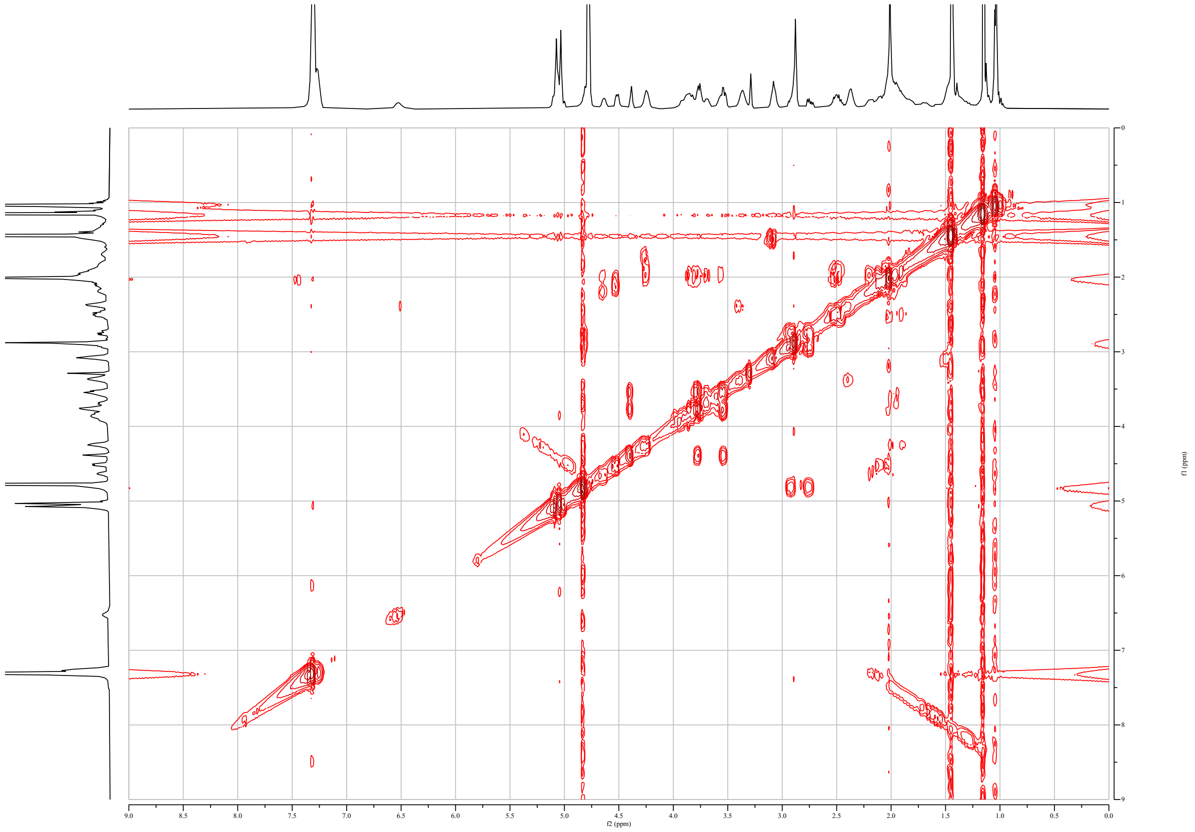

2D TOCSY NMR (400 MHz) Spectrum of **35** in CD_3OD.

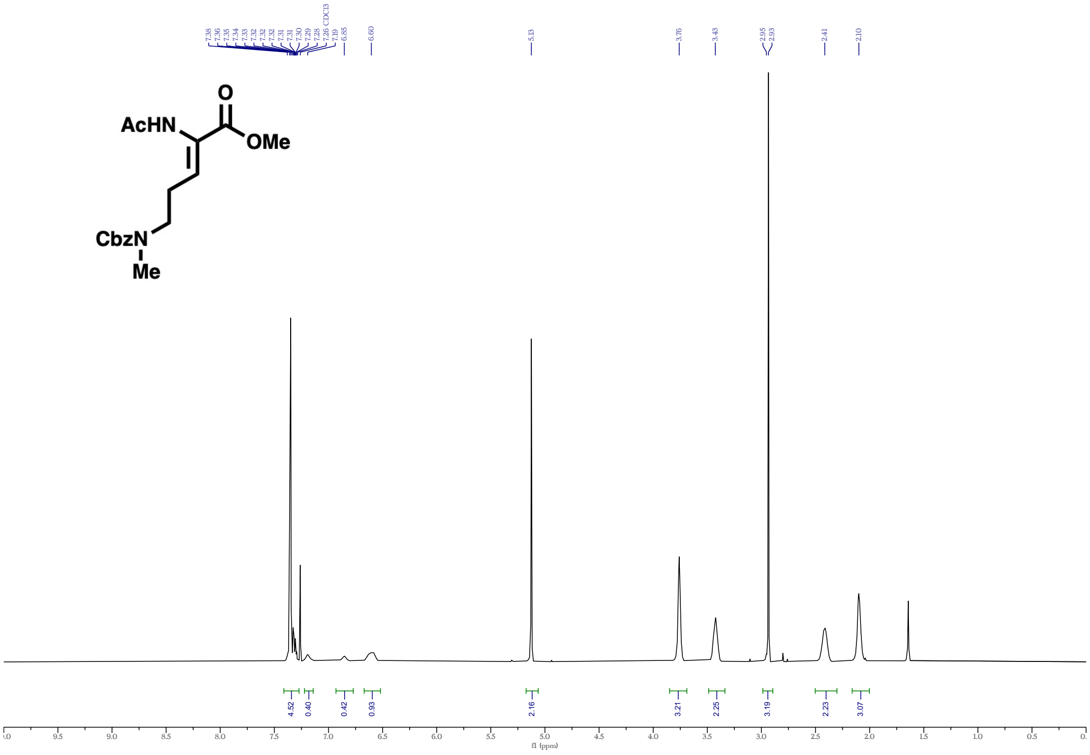

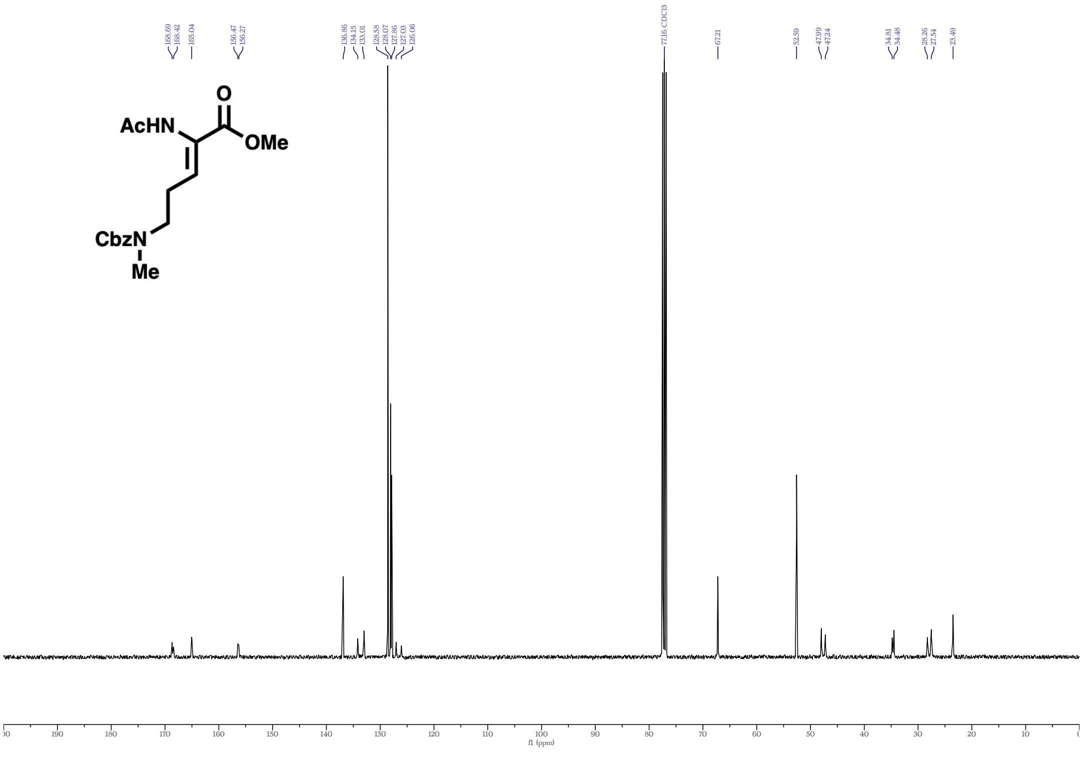

^{1}H NMR (400 MHz) and $^{13}C\{^{1}H\}$ NMR (100 MHz) Spectra of **46** in $CDCl_3$.

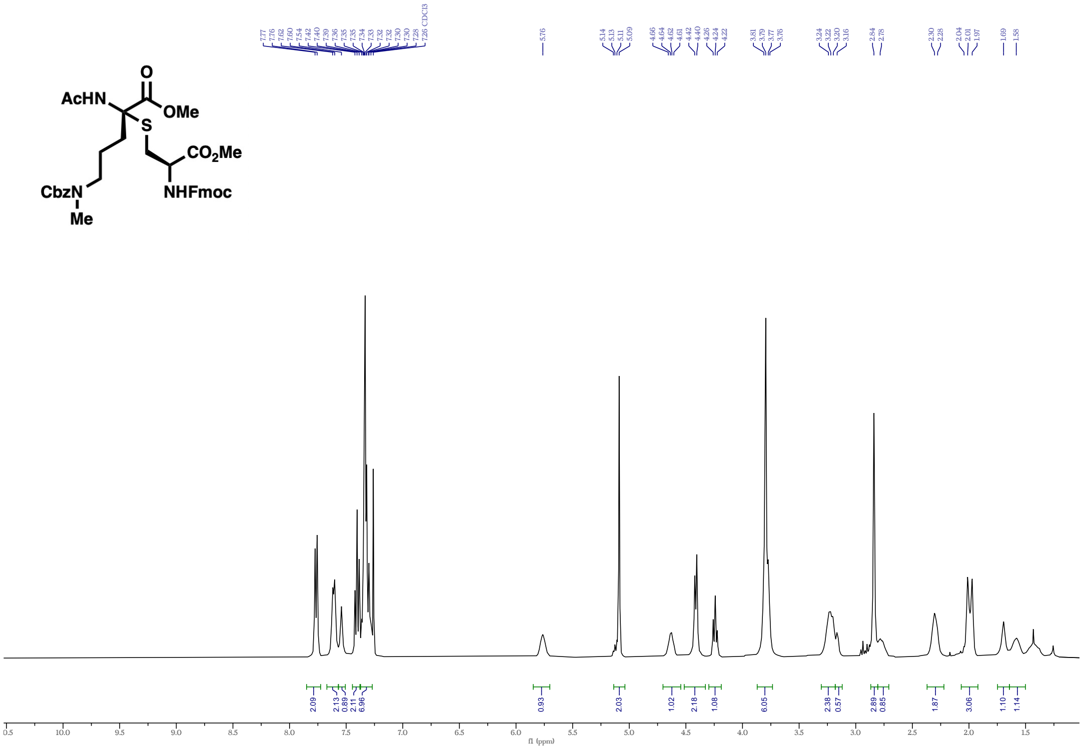

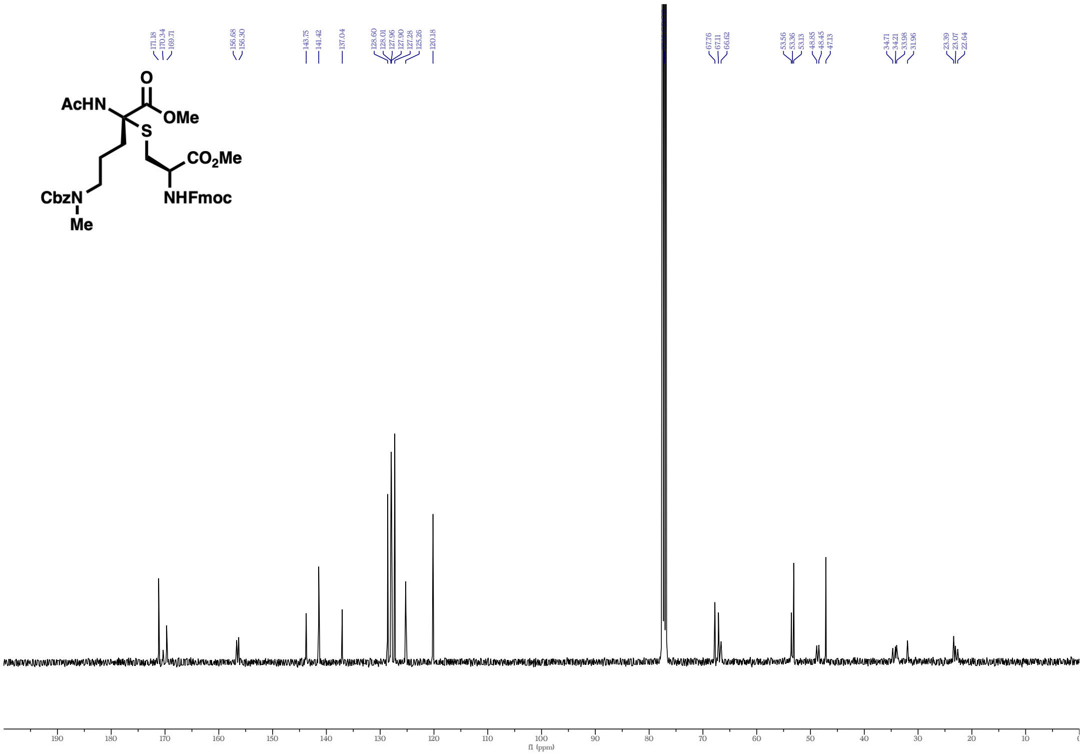

^{1}H NMR (400 MHz) and ^{13}C{^{1}H} NMR (100 MHz) Spectra of D-**49** in $CDCl_3$.

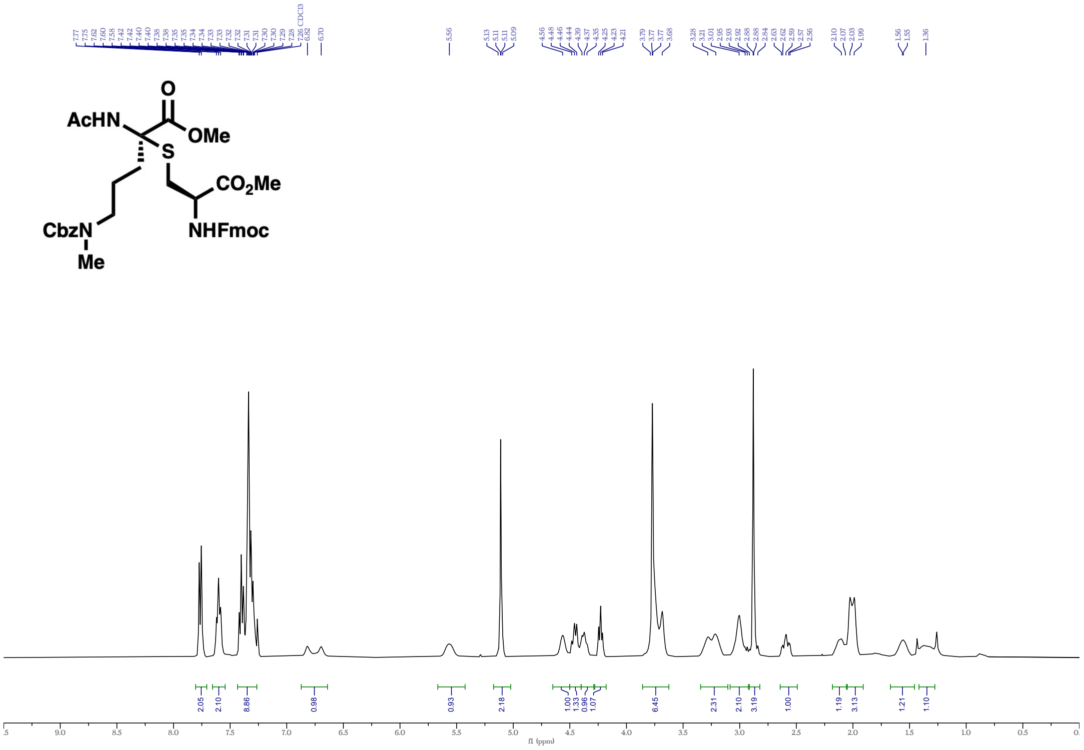

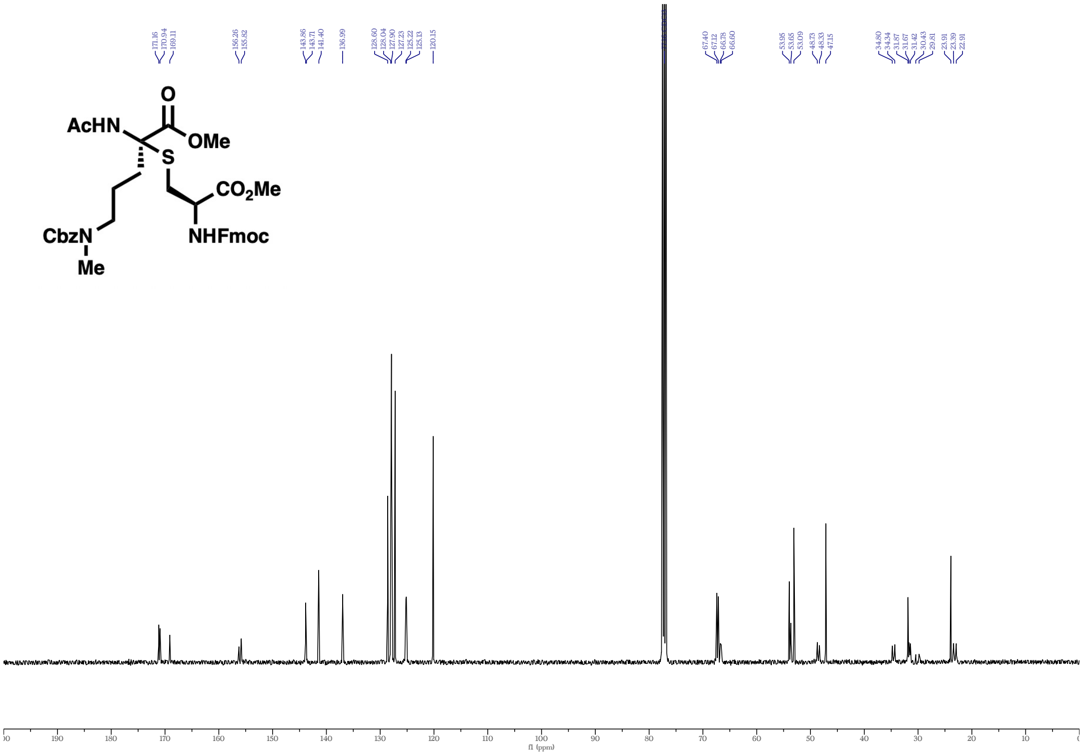

^{1}H NMR (400 MHz) and ^{13}C{^{1}H} (100 MHz) NMR Spectra of L-**49** in $CDCl_3$.

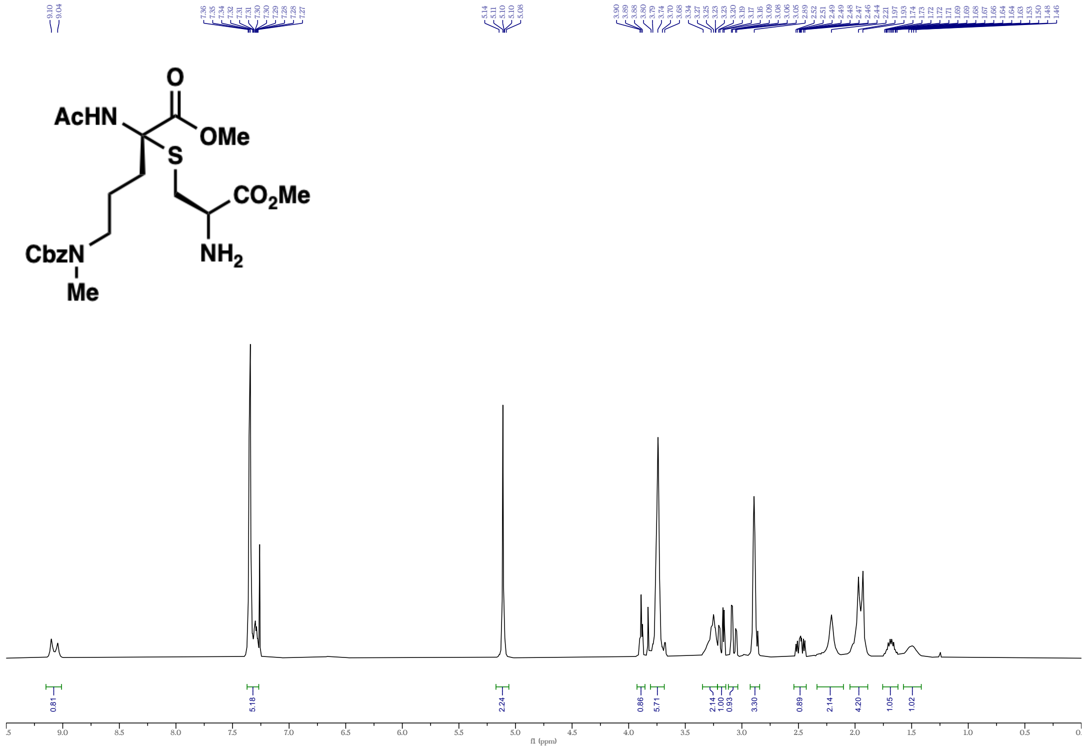

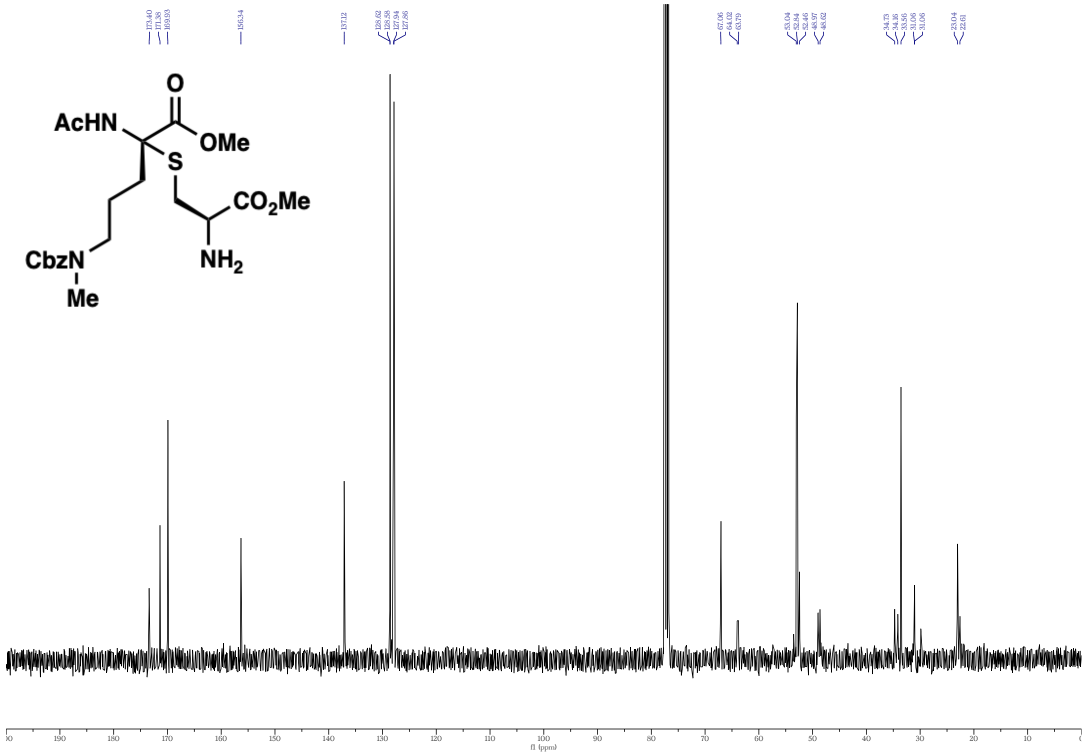

^{1}H NMR (400 MHz) and ^{13}C{^{1}H} NMR (100 MHz) Spectra of D-**50** in $CDCl_3$.

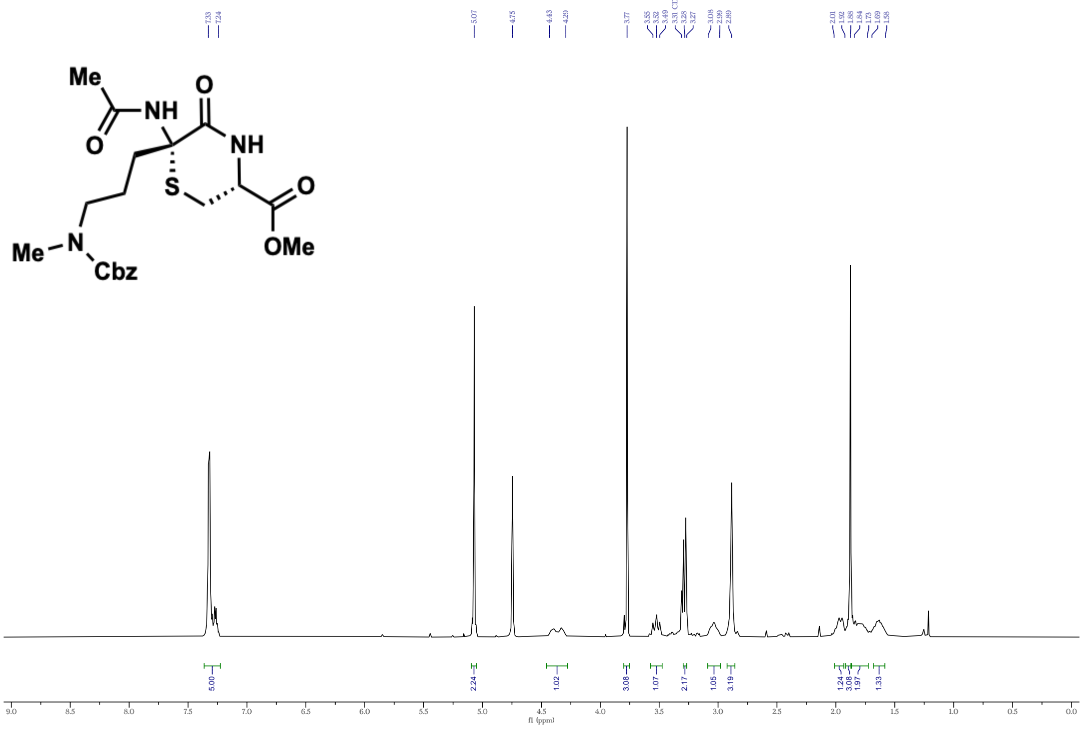

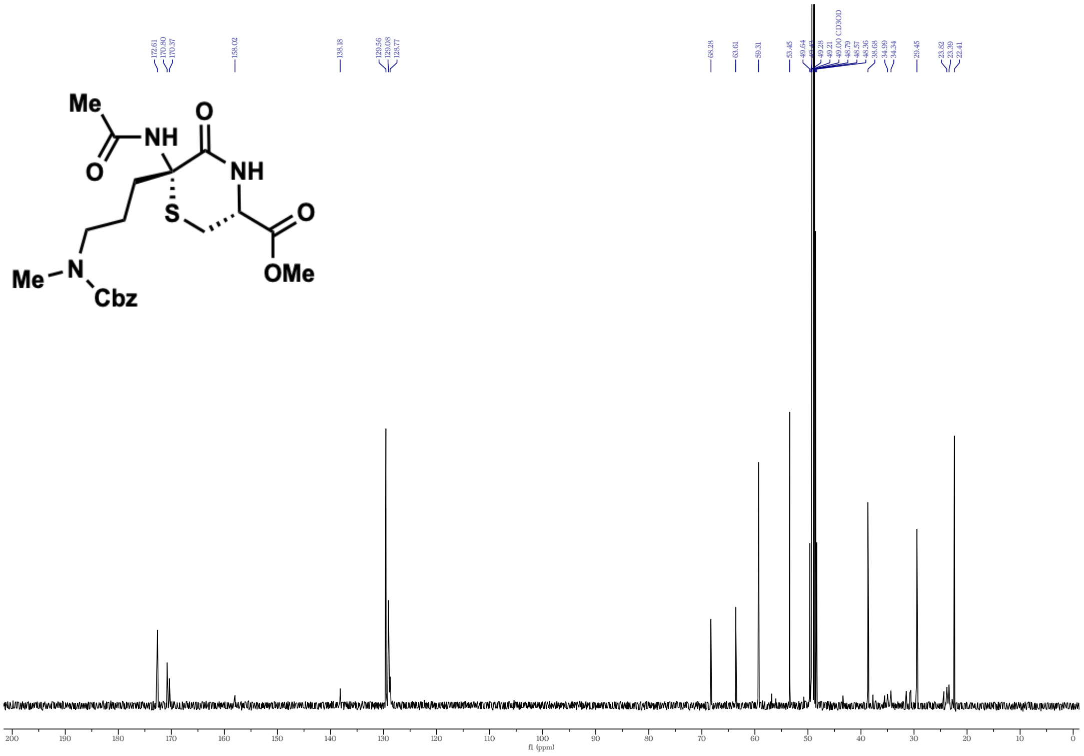

^{1}H NMR (400 MHz) and $^{13}C\{^{1}H\}$ NMR (100 MHz) Spectra of **51** in CD_3OD.

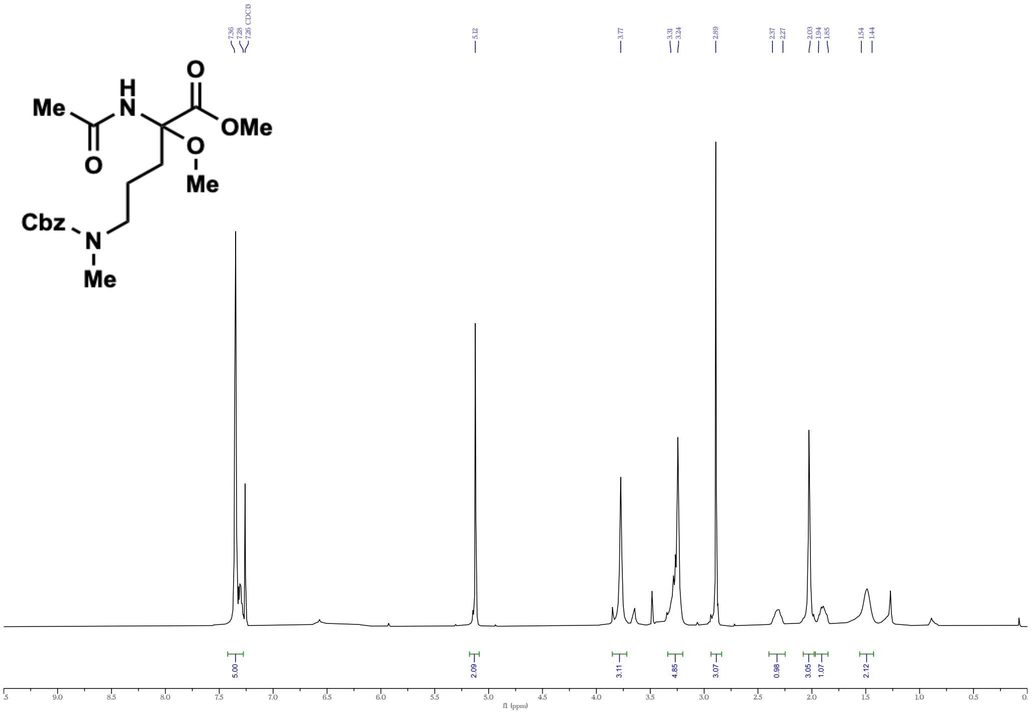

^{1}H NMR (400 MHz) Spectrum of **52** in $CDCl_3$ at 50°C.

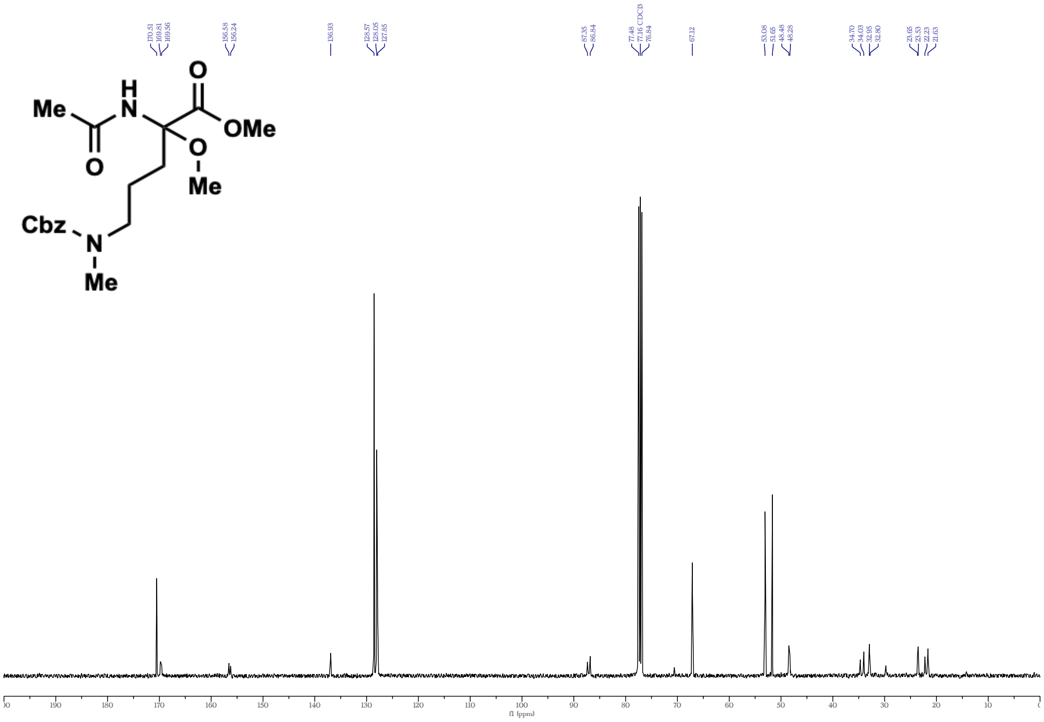

$^{13}C\{^{1}H\}$ NMR (100 MHz) Spectrum of **52** in $CDCl_3$.
