# Supplementary material for: Evolution of a Strategy for the Unified Synthesis of Enteropeptin Sactipeptides
Source: J Org Chem. 2026 Feb 23;91(9):3529–44. doi: 10.1021/acs.joc.5c03063 (PMC12973295; doi:10.1021/acs.joc.5c03063)
Supplement: Supplementary file 1 [file jo5c03063_si_001.zip › Compound 34 - TOCSY/4/pdata/1/email_yz-MKGOCPPS-TOCSY-CD3OD_4_1.pdf]

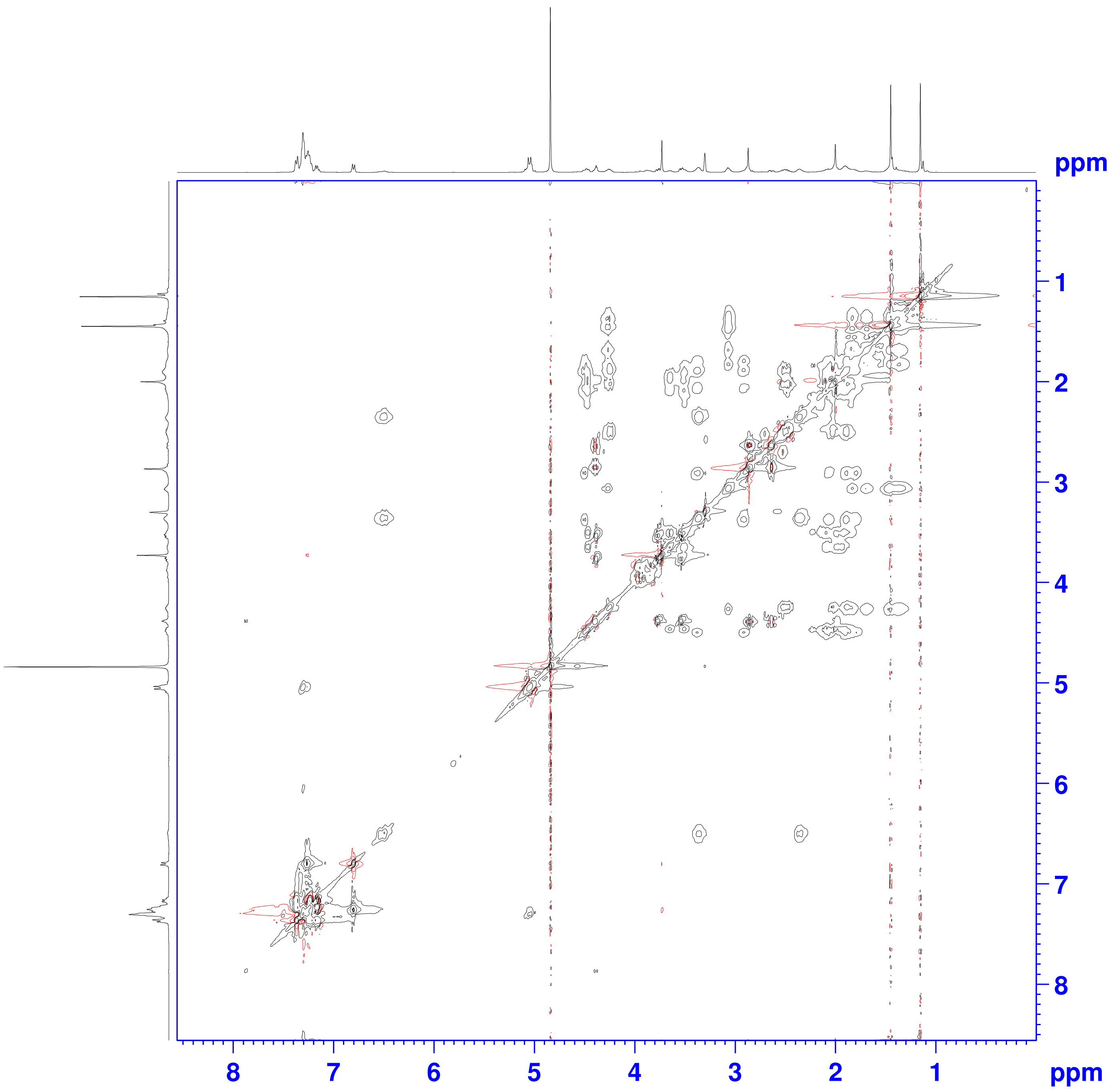

BRUKER

```
Current Data Parameters
NAME      yz-MKGOCPPS-TOCSY-CD3OD
EXPNO                    4
PROCNO                   1

F2 - Acquisition Parameters
Date_             20251026
Time                 15.30 h
INSTRUM             Avance
PROBHD    Z163739_0940 (
PULPROG           mlevphpp
TD                    2048
SOLVENT          CD3OD_SPE
NS                       5
DS                      16
SWH               3424.658 Hz
FIDRES            3.344392 Hz
AQ               0.2990080 sec
RG                      64
DW                 146.000 usec
DE                    6.50 usec
TE                   298.0 K
D0              0.00013691 sec
D1              1.95904005 sec
D9              0.08000000 sec
D11             0.03000000 sec
D12             0.00002000 sec
IN0             0.00029200 sec
L1                      36
TDav                     1
SFO1           400.1317244 MHz
NUC1                    1H
P1                    8.00 usec
P5                   20.01 usec
P6                   30.00 usec
P7                   60.00 usec
P17                2500.00 usec
PLW1           23.00000000 W
PLW10           1.63559997 W

======== F1 INDIRECT DIMENSION ========
td1       256
sw_F1     8.558825

F1 - Acquisition parameters
TD                     256
SFO1              400.1317 MHz
FIDRES           26.755136 Hz
SW                   8.559 ppm
FnMODE         States-TPPI

F2 - Processing parameters
SI                    1024
SF             400.1300118 MHz
WDW                  QSINE
SSB                      2
LB                       0 Hz
GB                       0
PC                    1.40

F1 - Processing parameters
SI                    1024
MC2            States-TPPI
SF             400.1300118 MHz
WDW                  QSINE
SSB                      2
LB                       0 Hz
GB                       0
```
