# Supplementary material for: Evolution of a Strategy for the Unified Synthesis of Enteropeptin Sactipeptides
Source: J Org Chem. 2026 Feb 23;91(9):3529–44. doi: 10.1021/acs.joc.5c03063 (PMC12973295; doi:10.1021/acs.joc.5c03063)
Supplement: Supplementary file 1 [file jo5c03063_si_001.zip › Compound 35 - COSY/3/pdata/1/email_yz-05500-MKGOCPPS-SH-COSY_3_1.pdf]

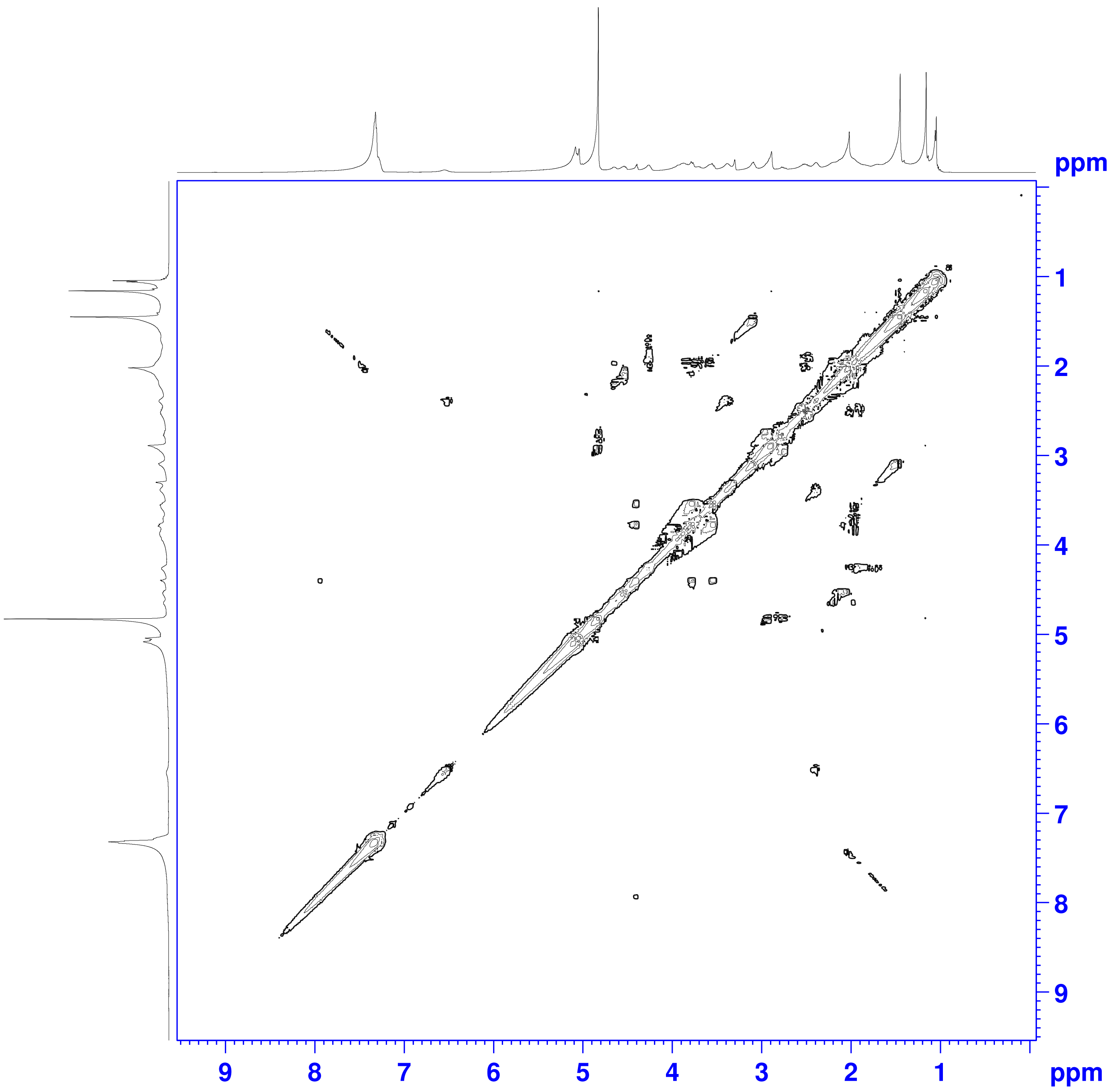

Current Data Parameters
NAME yz-05500-MKGOCPPS-SH-COSY
EXPNO 3
PROCNO 1

F2 - Acquisition Parameters
Date_ 20251030
Time 19.56 h
INSTRUM Avance
PROBHD Z163739_0940 (
PULPROG cosygpppqf
TD 2048
SOLVENT CD3OD_SPE
NS 4
DS 16
SWH 3846.154 Hz
FIDRES 3.756010 Hz
AQ 0.2662400 sec
RG 32
DW 130.000 usec
DE 6.50 usec
TE 298.0 K
D0 0.00000300 sec
D1 1.92627203 sec
D11 0.03000000 sec
D12 0.00002000 sec
D13 0.00000400 sec
D16 0.00020000 sec
IN0 0.00026000 sec
TDav 1
SFO1 400.1319073 MHz
NUC1 1H
P0 8.00 usec
P1 8.00 usec
P17 2500.00 usec
PLW1 23.00000000 W
PLW10 1.63559997 W
GPNAM[1] SMSQ10.100
GPZ1 10.00 %
P16 1000.00 usec

======== F1 INDIRECT DIMENSION ========
td1 128
sw_F1 9.612215

F1 - Acquisition parameters
TD 128
SFO1 400.1319 MHz
FIDRES 60.096153 Hz
SW 9.612 ppm
FnMODE QF

F2 - Processing parameters
SI 1024
SF 400.1300127 MHz
WDW QSINE
SSB 0
LB 0 Hz
GB 0
PC 1.40

F1 - Processing parameters
SI 1024
MC2 QF
SF 400.1300127 MHz
WDW QSINE
SSB 0
LB 0 Hz
GB 0
