# Supplementary material for: Evolution of a Strategy for the Unified Synthesis of Enteropeptin Sactipeptides
Source: J Org Chem. 2026 Feb 23;91(9):3529–44. doi: 10.1021/acs.joc.5c03063 (PMC12973295; doi:10.1021/acs.joc.5c03063)
Supplement: Supplementary file 1 [file jo5c03063_si_001.zip › Compound 48 - 13C/3/pdata/1/email_yz-AcNH-DhE-13C-CDCl3_3_1.pdf]

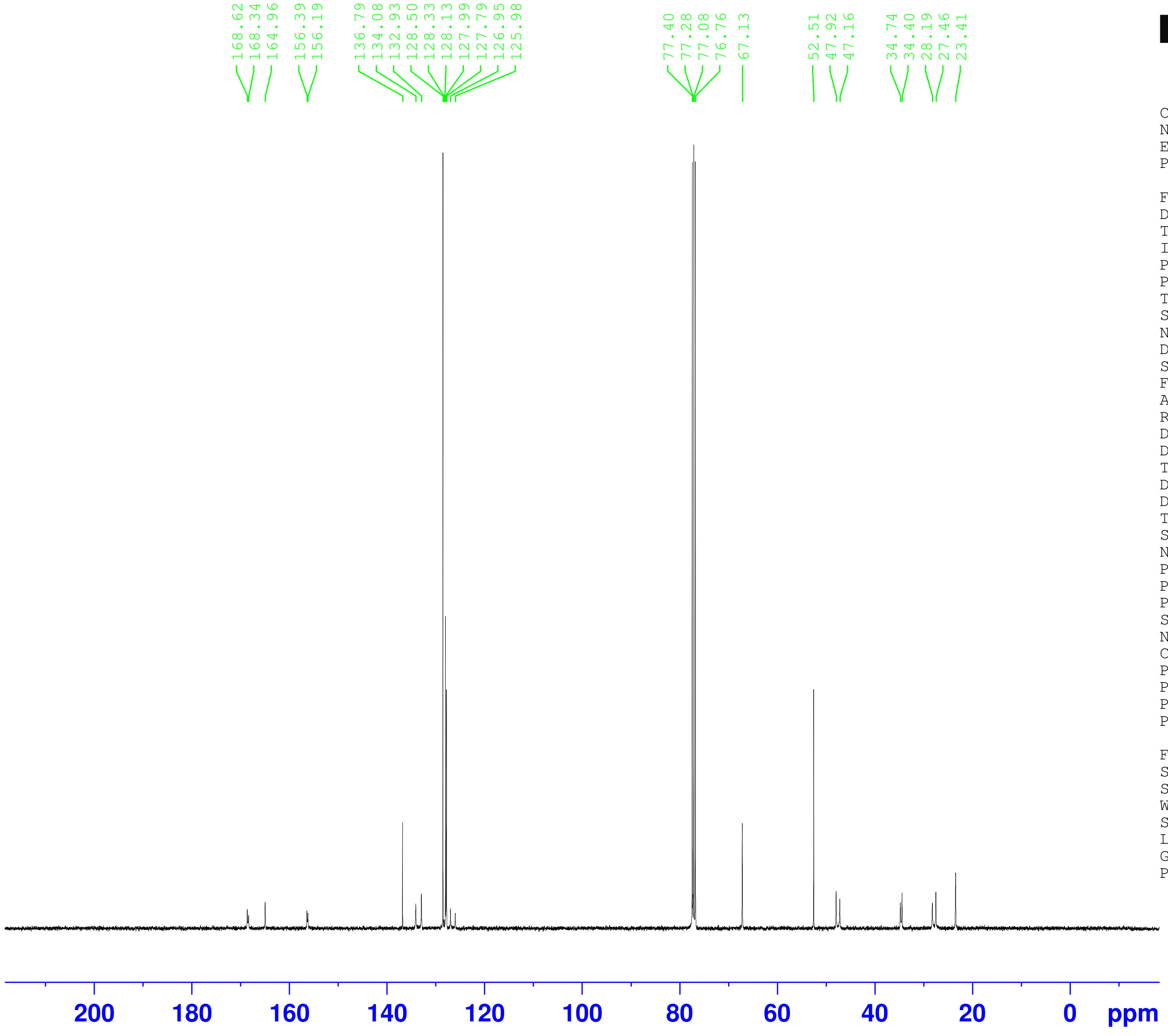


Current Data Parameters

| | |
|---|---|
| NAME | yz-AcNH-DhE-13C-CDCl3 |
| EXPNO | 3 |
| PROCNO | 1 |

F2 - Acquisition Parameters

| | |
|---|---|
| Date_ | 20251111 |
| Time | 23.02 h |
| INSTRUM | Avance |
| PROBHD | Z163739_0940 ( |
| PULPROG | zgpg30 |
| TD | 65536 |
| SOLVENT | CDCl3 |
| NS | 2048 |
| DS | 4 |
| SWH | 23809.524 Hz |
| FIDRES | 0.726609 Hz |
| AQ | 1.3762560 sec |
| RG | 101 |
| DW | 21.000 usec |
| DE | 6.50 usec |
| TE | 298.0 K |
| D1 | 2.00000000 sec |
| D11 | 0.03000000 sec |
| TD0 | 1 |
| SFO1 | 100.6228298 MHz |
| NUC1 | 13C |
| P0 | 2.67 usec |
| P1 | 8.00 usec |
| PLW1 | 87.00000000 W |
| SFO2 | 400.1316005 MHz |
| NUC2 | 1H |
| CPDPRG[2 | waltz65 |
| PCPD2 | 90.00 usec |
| PLW2 | 23.00000000 W |
| PLW12 | 0.18173000 W |
| PLW13 | 0.09140800 W |

F2 - Processing parameters

| | |
|---|---|
| SI | 32768 |
| SF | 100.6127685 MHz |
| WDW | EM |
| SSB | 0 |
| LB | 1.00 Hz |
| GB | 0 |
| PC | 1.40 |
