# Supplementary material for: Evolution of a Strategy for the Unified Synthesis of Enteropeptin Sactipeptides
Source: J Org Chem. 2026 Feb 23;91(9):3529–44. doi: 10.1021/acs.joc.5c03063 (PMC12973295; doi:10.1021/acs.joc.5c03063)
Supplement: Supplementary file 1 [file jo5c03063_si_001.zip › Compound 52 - 1H/pdata/1/SS-04-006-pdt-1H-CDCl3_2_1.pdf]

SS-04-006-pdt-1H-CDCl3

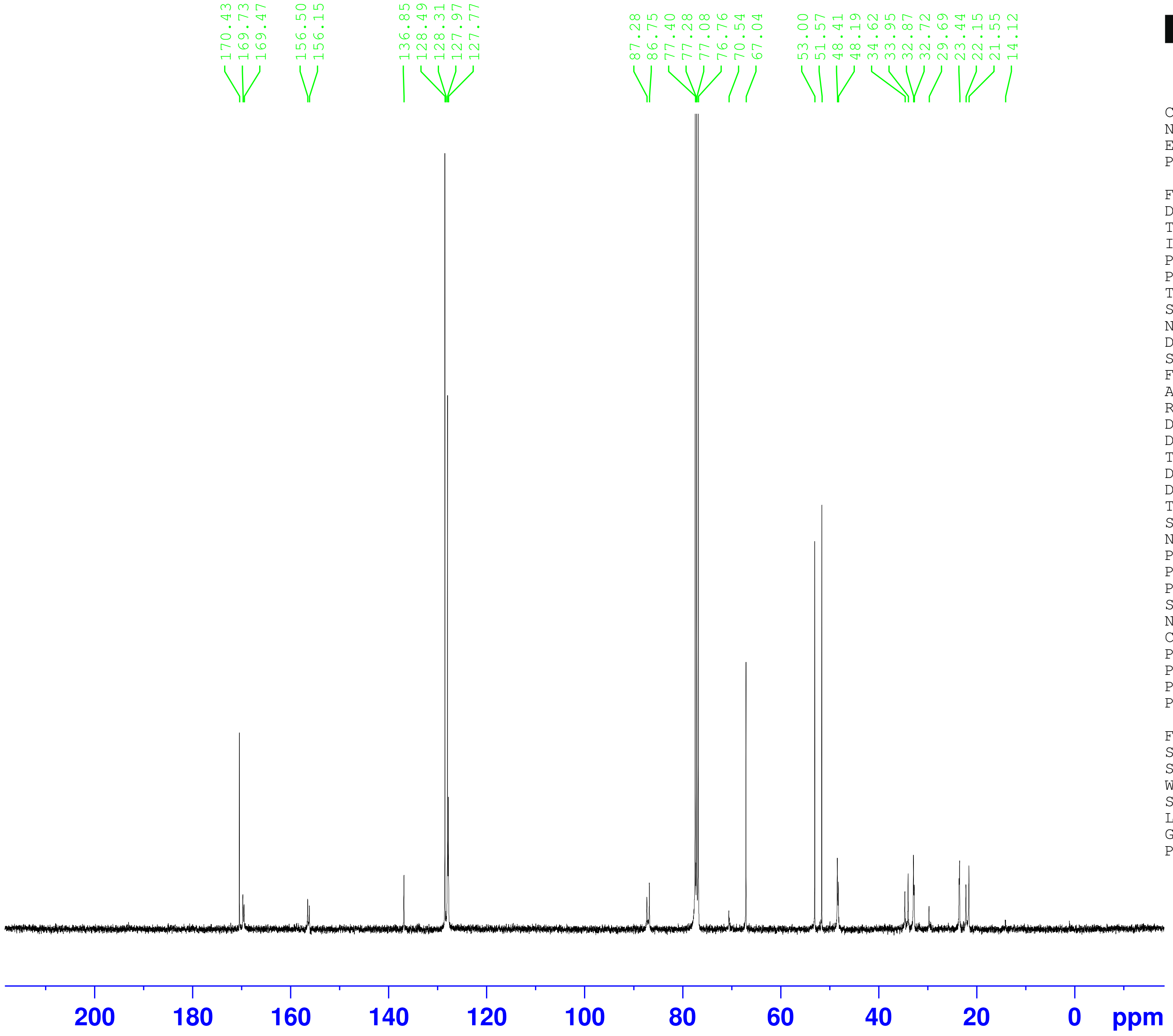


```
Current Data Parameters
NAME      SS-04-006-pdt-1H-CDCl3
EXPNO                          2
PROCNO                         1

F2 - Acquisition Parameters
Date_                   20251124
Time                      1.33 h
INSTRUM                   Avance
PROBHD      Z163739_0940 (
PULPROG                  zgpg30
TD                         65536
SOLVENT                    CDCl3
NS                          2048
DS                             4
SWH                   23809.524 Hz
FIDRES                 0.726609 Hz
AQ                    1.3762560 sec
RG                           101
DW                        21.000 usec
DE                          6.50 usec
TE                         298.0 K
D1                    2.00000000 sec
D11                   0.03000000 sec
TD0                            1
SFO1                 100.6228298 MHz
NUC1                         13C
P0                          2.67 usec
P1                          8.00 usec
PLW1                 87.00000000 W
SFO2                 400.1316005 MHz
NUC2                          1H
CPDPRG[2                 waltz65
PCPD2                      90.00 usec
PLW2                 23.00000000 W
PLW12                 0.18173000 W
PLW13                 0.09140800 W

F2 - Processing parameters
SI                         32768
SF                   100.6127685 MHz
WDW                           EM
SSB                            0
LB                          1.00 Hz
GB                             0
PC                          1.40
```
