# Supplementary material for: Evolution of a Strategy for the Unified Synthesis of Enteropeptin Sactipeptides
Source: J Org Chem. 2026 Feb 23;91(9):3529–44. doi: 10.1021/acs.joc.5c03063 (PMC12973295; doi:10.1021/acs.joc.5c03063)
Supplement: Supplementary file 1 [file jo5c03063_si_001.zip › Compound 52 - 13C/1/pdata/1/email_SS-04-006-VTtube-50C-1H-CDCl3_1_1.pdf]

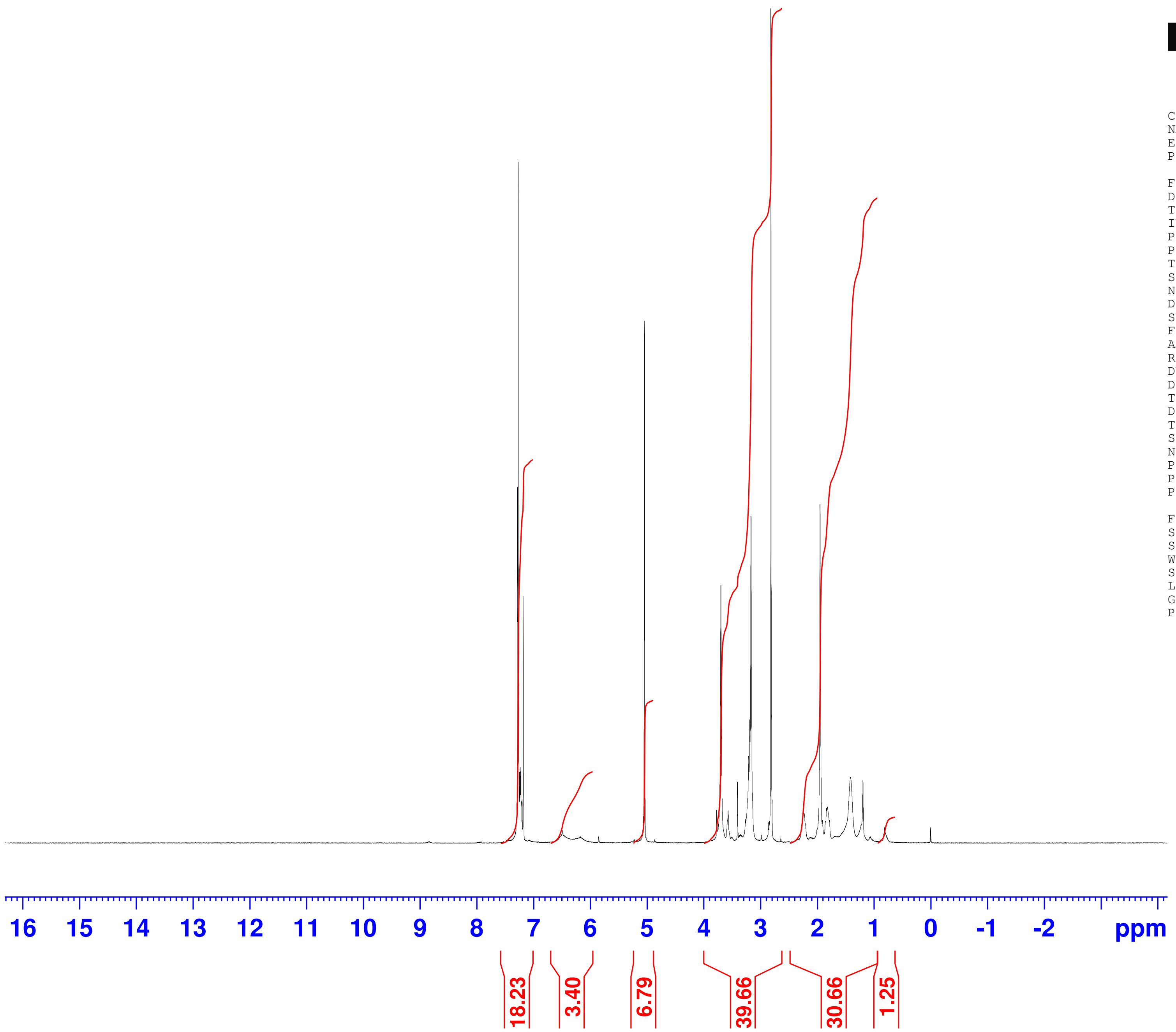

BRUKER

```
Current Data Parameters
NAME     SS-04-006-VTtube-50C-1H-CDCl3
EXPNO                    1
PROCNO                   1

F2 - Acquisition Parameters
Date_             20251125
Time                 16.33 h
INSTRUM             Avance
PROBHD   Z163739_0940 (
PULPROG               zg30
TD                   65536
SOLVENT              CDCl3
NS                      16
DS                       2
SWH               8196.721 Hz
FIDRES            0.250144 Hz
AQ               3.9976959 sec
RG                     101
DW                  61.000 usec
DE                   13.89 usec
TE                   323.0 K
D1              1.00000000 sec
TD0                      1
SFO1           400.1324708 MHz
NUC1                    1H
P0                    2.67 usec
P1                    8.00 usec
PLW1           23.00000000 W

F2 - Processing parameters
SI                   65536
SF             400.1300406 MHz
WDW                     EM
SSB                      0
LB                    0.30 Hz
GB                       0
PC                    1.00
```
