# Supplementary material for: Evolution of a Strategy for the Unified Synthesis of Enteropeptin Sactipeptides
Source: J Org Chem. 2026 Feb 23;91(9):3529–44. doi: 10.1021/acs.joc.5c03063 (PMC12973295; doi:10.1021/acs.joc.5c03063)
Supplement: Supplementary file 1 [file jo5c03063_si_001.zip › Compound L-49 - 13C/3/pdata/1/email_yz-AcNH-DhE-Sac-P2-13C-CDCl3_3_1.pdf]

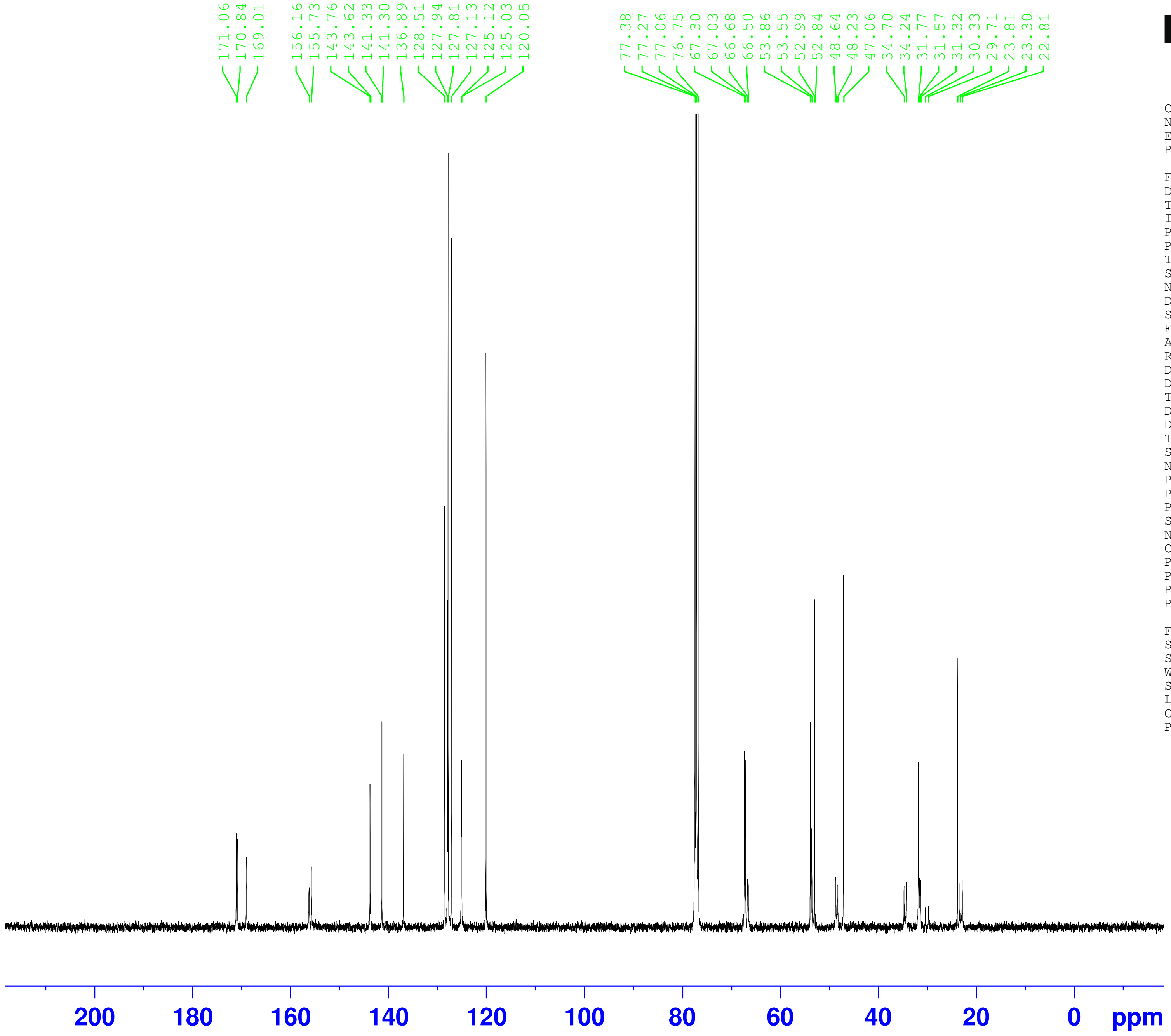

Current Data Parameters
NAME yz-AcNH-DhE-Sac-P2-13C-CDCl3
EXPNO 3
PROCNO 1

F2 - Acquisition Parameters
Date_ 20251113
Time 2.49 h
INSTRUM Avance
PROBHD Z163739_0940 (
PULPROG zgpg30
TD 65536
SOLVENT CDCl3
NS 4800
DS 4
SWH 23809.524 Hz
FIDRES 0.726609 Hz
AQ 1.3762560 sec
RG 101
DW 21.000 usec
DE 6.50 usec
TE 298.0 K
D1 2.00000000 sec
D11 0.03000000 sec
TD0 1
SFO1 100.6228298 MHz
NUC1 13C
P0 2.67 usec
P1 8.00 usec
PLW1 87.00000000 W
SFO2 400.1316005 MHz
NUC2 1H
CPDPRG[2 waltz65
PCPD2 90.00 usec
PLW2 23.00000000 W
PLW12 0.18173000 W
PLW13 0.09140800 W

F2 - Processing parameters
SI 32768
SF 100.6127685 MHz
WDW EM
SSB 0
LB 1.00 Hz
GB 0
PC 1.40
